# Mutations in *COMP* cause familial carpal tunnel syndrome

**DOI:** 10.1038/s41467-020-17378-z

**Published:** 2020-07-20

**Authors:** Chunyu Li, Ni Wang, Alejandro A. Schäffer, Xilin Liu, Zhuo Zhao, Gene Elliott, Lisa Garrett, Nga Ting Choi, Yueshu Wang, Yufa Wang, Cheng Wang, Jin Wang, Danny Chan, Peiqiang Su, Shusen Cui, Yingzi Yang, Bo Gao

**Affiliations:** 1 Department of Hand Surgery, China-Japan Union Hospital of Jilin University, Changchun, China; 2 School of Biomedical Sciences, Li Ka Shing Faculty of Medicine, The University of Hong Kong, Hong Kong, China; 3 National Center for Biotechnology Information and National Cancer Institute, National Institutes of Health, Bethesda, MD US; 4 National Human Genome Research Institute, National Institutes of Health, Bethesda, MD US; 5 Department of Orthopaedic Surgery, First Affiliated Hospital of Sun Yat-sen University, Guangzhou, China; 6 Department of Developmental Biology, Harvard School of Dental Medicine, Harvard Stem Cell Institute, Harvard University, Boston, MA US

**Keywords:** Mechanisms of disease, Disease genetics, Mutation, Genetics research, Disease genetics

## Abstract

Carpal tunnel syndrome (CTS) is the most common peripheral nerve entrapment syndrome, affecting a large proportion of the general population. Genetic susceptibility has been implicated in CTS, but the causative genes remain elusive. Here, we report the identification of two mutations in cartilage oligomeric matrix protein (COMP) that segregate with CTS in two large families with or without multiple epiphyseal dysplasia (MED). Both mutations impair the secretion of COMP by tenocytes, but the mutation associated with MED also perturbs its secretion in chondrocytes. Further functional characterization of the CTS-specific mutation reveals similar histological and molecular changes of tendons/ligaments in patients’ biopsies and the mouse models. The mutant COMP fails to oligomerize properly and is trapped in the ER, resulting in ER stress-induced unfolded protein response and cell death, leading to inflammation, progressive fibrosis and cell composition change in tendons/ligaments. The extracellular matrix (ECM) organization is also altered. Our studies uncover a previously unrecognized mechanism in CTS pathogenesis.

## Abstract (web summary)

Familial carpal tunnel syndrome (CTS) is common, but causal genes are not characterized. Here the authors report two CTS-related mutations in two large families that impair secretion of COMP in tenocytes, leading to ER stress-induced unfolded protein response, inflammation and fibrosis in patients and mouse models.

## Introduction

CTS, characterized by paresthesia, pain, and numbness in the hands due to excessive pressure on the median nerve, is a common disorder treated by hand surgeons^1,2^. CTS is often caused by swelling and thickening of soft connective tissues surrounding the median nerve within the carpal tunnel, rather than a problem with the nerve itself^3–9^. Enlarged and fibrotic soft connective tissues can result in compression or traction on the median nerve at the carpal tunnel. CTS affects 1–5% of the general population, and is a leading cause of work disability^10,11^. In the United States, CTS causes $1–2 billion in annual direct costs^12^; the number of CTS surgeries is continually increasing^13^. Without appropriate treatment, CTS can lead to nerve damage, permanent muscle atrophy, or loss of hand function. However, despite its prevalence and considerable psychological and economic impact, the molecular and pathogenic bases of CTS remain largely unknown.

While CTS is mostly sporadic and idiopathic, familial occurrence of CTS in 17–39% of cases suggests a genetic predisposition^14,15^. CTS is also a symptom in two syndromic diseases, transthyretin-related amyloidosis and Charcot–Marie–Tooth (CMT) disease, in which CTS is one of many manifestations of neuropathy caused by either defects in the nerve itself or amyloid deposits^16,17^. *TTR* and *SH3TC2* mutations have been identified in amyloidosis and CMT patients with CTS, respectively^18,19^. A few nonsyndromic CTS pedigrees with autosomal-dominant inheritance have been reported, but no causal genes or loci were identified^16,17,20–25^. Variants in some genes, including *BGN*, *ACAN*, *COL5A1*, and *IL6R*, have been associated with the risk of sporadic CTS^26–28^. Since environmental factors, such as repetitive hand use, contribute to CTS by preferentially affecting the dominant hand, the high percentage of bilateral CTS (about 60%)^29,30^ and twin studies (estimated 46% heritability)^31^ suggests that genetic factors significantly influence CTS susceptibility. A recent genome-wide association study (GWAS) of CTS identified a couple of CTS-associated variants within genes implicated in growth and ECM architecture, although the causative link has not been established^32^.

Here, we report identification of two causative mutations in *COMP* gene in two large families with a dominant inheritance pattern of bilateral CTS. Patients in Family 1 exhibit severe CTS symptoms only, but patients in Family 2 have both CTS and multiple epiphyseal dysplasia (MED). Functional studies in the CTS-specific patients’ tissues and in genetically modified animal models reveal the molecular and pathogenic mechanism of the COMP mutation. Our studies reveal critical roles of ECM proteins and cellular stress response in the pathogenesis of CTS.

## Results

### Patients and clinical examination

**Fig. 1:**
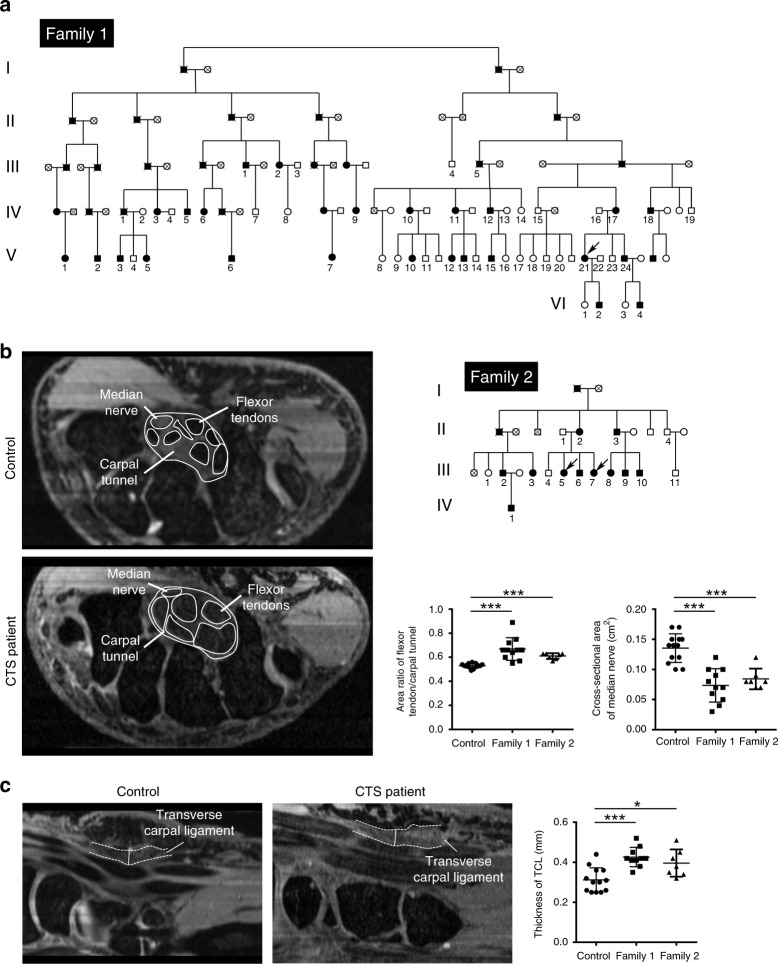
Pedigrees and clinical manifestations of familial carpal tunnel syndrome (CTS). **a** Two pedigrees of familial carpal tunnel syndrome. The pedigree diagram of Family 1 is simplified, also see Supplementary Fig. 1 for the full pedigree diagram. Squares and circles denote male and female family members, respectively. Solid and open symbols denote affected and unaffected family members, respectively. The individuals with numbers underneath indicate the family members recruited in this study. The arrows indicate the probands; Family 2 has two probands who were identified in a single search of hospital records. **b** Magnetic resonance imaging (MRI) results indicate enlarged digital flexor tendons and compressed median nerves in patients’ carpal tunnels (two-tailed *t* test, ***p = 2.98 × 10^−5^ (Family 1) and 2.07 × 10^−7^ (Family 2) for area ratio of flexor tendon/carpal tunnel; ***p = 6.21 × 10^−6^ (Family 1) and 8.94 × 10^−5^ (Family 2) for median nerve, error bars are ± SEM). **c** MRI results indicate thickened transverse carpal ligaments (TCLs) in patients’ carpal tunnels (two-tailed *t* test, ***p = 7.90 × 10^−5^ for Family 1 and *p = 0.011 for Family 2, error bars are ± SEM). The hook of the hamate bone was used as a reference position, and the thickness of the TCLs was measured at the carpometacarpal level. *n* = 13 in controls, *n* = 11 in Family 1, and *n* = 7 in Family 2. Source data are provided as a Source Data file.

We previously described a pedigree with isolated bilateral CTS and autosomal-dominant inheritance^33^. Further genealogical investigations identified a larger multiplex family (Family 1 in Fig. 1a and Supplementary Fig. 1). Initial CTS symptoms in most affected family members occurred at 20–30 years of age, earlier than the typical onset of CTS between 45 and 64^34^. Median nerve entrapment in affected family members caused pain, paranesthesia, and numbness in the thumb, middle, and index fingers that are innervated by the median nerve. Symptoms were exacerbated at night, by overuse or by exercise. Patients had typical CTS symptoms and positive test results, including Tinel’s sign, Phalen sign, two-point discrimination, and electrophysiological tests (NCS/EMG, nerve-conduction studies, and electromyography) (Supplementary Table 1). Thenar atrophy, digital trophic ulcers, and digital necrosis were observed in some family members. Hand surgeries revealed soft connective tissue swelling and thickening in the carpal tunnels, similar to, but more severe than what has been found in other sporadic CTS patients. The affected family members’ transverse carpal ligaments (TCLs) and flexor tendons were severely enlarged, restricting the space within the carpal tunnel, leading to median nerve compression (Supplementary Fig. 2a).

We also reviewed hospital records of (supposedly) sporadic CTS patients who underwent carpal tunnel release surgeries. Two sister patients drew our attention (III-5 and III-7 of Family 2 in Fig. 1a). Genealogical investigation revealed that their family included 11 living CTS patients. Compared with Family 1, the CTS symptoms of Family 2 are less severe. The age of onset is between 30 and 50 s (Supplementary Table 1). Family 2 patients had previously complained of joint pains and were further examined by orthopedists. The orthopedists suspected that Family 2 patients have MED, which is characterized by epiphyseal deformity with joint pain and stiffness. However, no joint or growth-plate defects were observed in CTS patients in Family 1.

Some affected members from each family, as well as familial and nonfamilial controls, were examined by magnetic resonance imaging (MRI), which revealed swelling of flexor tendons and smaller median nerves in patients’ carpal tunnels (Fig. 1b). Like we observed in the carpal tunnel release surgery, MRI showed that the patients’ TCLs were significantly thickened (Fig. 1c). Flexor pollicis longus tendons and Achilles tendons of Family 1 patients also tended to be bigger (Supplementary Fig. 2b, c), suggesting a systemic change in patients’ tendons/ligaments. Because MED patients may exhibit ligamentous laxity, we further assessed the mobility of their thumbs, little fingers, wrists, and ankles. While no joint hypermobility was observed in Family 2 patients, joint hypomobility was prominent in Family 1 patients (Supplementary Fig. 3).

### Histological analysis of patients’ biopsies

**Fig. 2:**
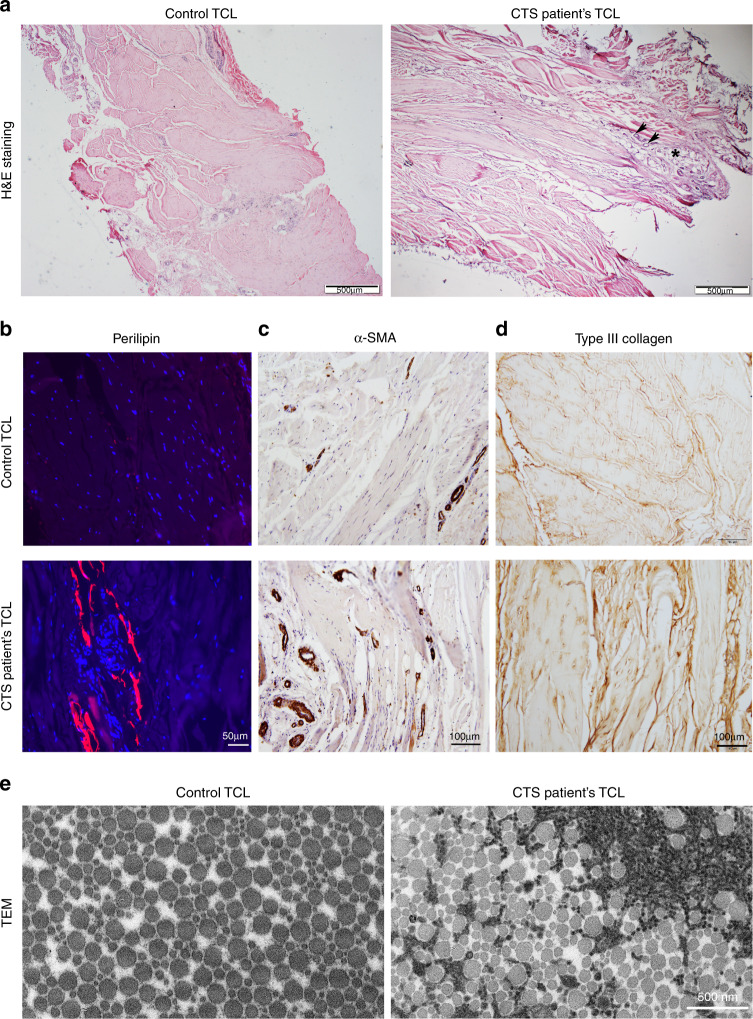
Histological analysis of CTS patients’ biopsies. Transverse carpal ligament (TCL) tissues collected from controls (*n* = 2) and Family 1 patients (*n* = 3) were analyzed. **a** H&E staining reveals collagen fragmentation, edema (asterisk), and vascular structures (arrows) in patients. **b**–**d** Immunostainings indicate lipomatosis (perilipin) and fibrosis (α-SMA and type III collagen) in patients’ TCLs. **e** Transmission electron microscopy (TEM) reveals a number of ectopic small fibrils and contrast difference in patients’ extracellular matrix (ECM). Source data are provided as a Source Data file.

To understand the mechanism underlying soft connective tissues’ swelling and thickening, we performed histological analysis on TCL tissues from Family 1 patients and unrelated controls. Consistent with MRI findings, the patients’ TCLs were markedly thicker than controls (Fig. 2a). In patients’ samples, we identified collagen fragmentation, edema (asterisk in Fig. 2a), neovascular structure (arrows in Fig. 2a), and lipomatosis (Fig. 2b), with mild amyloidosis and few skeletal muscles (Supplementary Fig. 4a). We also found a large amount of alpha-smooth muscle actin (α-SMA)-positive cells (Fig. 2c). As α-SMA also labels blood vessels, its co-staining with CD34, an endothelial cell marker, was used to count α-SMA^+^CD34^−^ myofibroblasts. This revealed an increased number of myofibroblasts and thickening of the vascular walls in CTS patients’ TCLs (Supplementary Fig. 4b). The upregulation of type III collagen and FSP1 in CTS patients also indicates strong fibrosis in TCLs (Fig. 2d and Supplementary Fig. 4c). These histological features in the familial CTS patients resembled features reported in sporadic, idiopathic CTS patients^35–39^. Transmission electron microscopy (TEM) identified ectopic small fibrils and contrast difference in ECM of patients’ TCLs (Fig. 2e), suggesting a change in ECM composition and organization. Immuno-TEM showed that these ectopic small fibrils contained several main components of ECM, such as type I collagen, type III collagen, and COMP. Type III collagen appeared more abundant in the ectopic small fibrils than in the normal fibrils (Supplementary Fig. 5). Similar changes in ECM and fibrosis were also observed in patients’ flexor tendons and subsynovial connective tissues (SSCT) (Supplementary Figs. 6a, b and 7a, b).

### Identification of COMP mutations

**Fig. 3:**
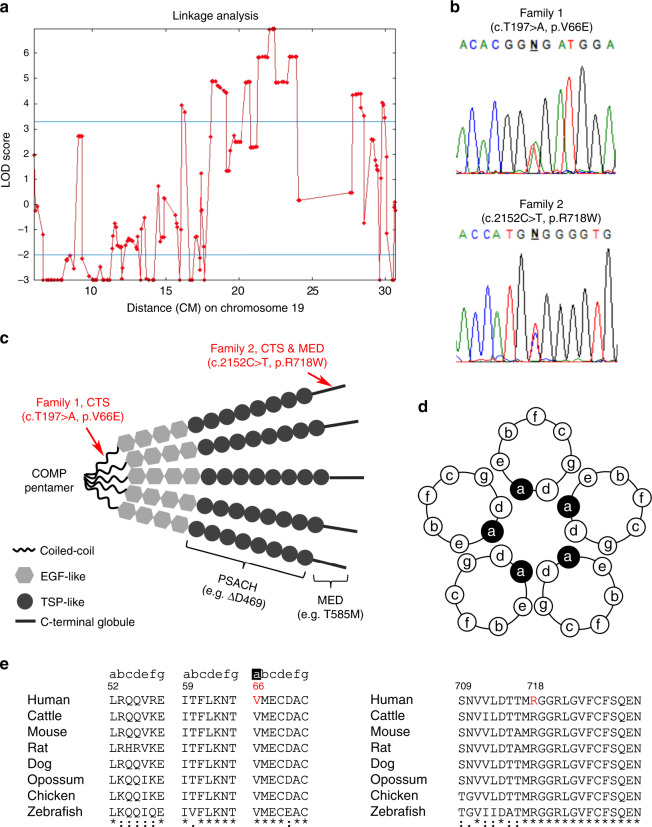
Identification of *COMP* mutations. **a** Linkage analysis of Family 1. Multimarker analysis of SNP genotyping results by Superlink-SNP reveals a locus on Chromosome 19p with the maximum LOD score more than 6. **b** Sanger sequencing chromatograms of the identified p.V66E and p.R718W heterozygous mutations in *COMP* in Family 1 and Family 2, respectively. **c** Schematic showing different domains of COMP protein and mutation spectrum of PSACH, MED, and CTS. The *COMP* mutation causing CTS in Family 1 locates in the N-terminal coiled-coil domain that mediates the formation of COMP homopentamer, while the COMP mutation in Family 2 is in the C-terminal globular domain, which was also reported causing MED. **d** Schematic showing how coiled-coil domain mediates COMP pentamerization, and **e** the alignment of coiled-coil domain and C-terminal globular domain of COMP amino acid sequences across multiple species. The coiled-coil sequence is characterized by a seven-residue repeat (denoted as “abcdefg”). Valine 66 (red) at “a” position is a highly conserved amino acid located in the hydrophobic pocket of COMP (**d**). Arginine 718 (red) is also highly conserved.

To identify the causative mutations in these families, we first included 31 Family 1 members (17 affected and 14 unaffected) in genetic linkage analysis using genome-wide SNPs. The analysis identified one locus, 19p12-13.11, on chromosome 19 with maximum multimarker logarithm of odds (LOD) score over 6 (Fig. 3a). Further microsatellite analysis and haplotype reconstruction in 39 Family 1 members confirmed the segregation of a haplotype in patients (Supplementary Table 2). We then sequenced the entire targeted locus in six affected individuals and six healthy consanguineous relatives. In all six patients, we identified a heterozygous missense mutation, c.197 T>A, in exon 3 of the *COMP* gene, which encodes cartilage oligomeric matrix protein (NM_000095.3; NP_000086.2). This variant caused a valine (V) to glutamate (E) (p.V66E) substitution in the N-terminal coiled-coil domain of the COMP protein (Fig. 3b–e). In Family 2, by whole-exome sequencing, we identified a heterozygous missense mutation, c.2152 C>T, in *COMP*, which caused an arginine (R) to tryptophan (W) (p.R718W) substitution in the C-terminal globular domain (Fig. 3b, c, e). Both mutations were validated by Sanger sequencing. We confirmed in all recruited family members (numbered in Fig. 1a) that the mutations segregated perfectly with disease among individuals who are old enough to be affected (age >20 in Family 1 and age >30 in Family 2). The two mutations were not present in existing human genome variation databases.

COMP is a noncollagenous ECM protein and plays a role in matrix assembly and organization through interactions with other ECM proteins, such as collagen I, II, IX, and matrilins^40^. COMP forms homopentamers mediated by its N-terminal coiled-coil domain (amino acids 22–86)^41^ and catalyzes polymerization of collagen fibrils^42^. Numerous mutations in *COMP* have been identified in patients with MED and pseudoachondroplasia (PSACH). PSACH is a severe congenital skeletal dysplasia characterized by short-limbed dwarfism and bone deformations^43,44^. All COMP mutations causing MED or PSACH are in either the TSP type III repeats (amino acids 268–528) or the C-terminal globular domain (amino acids 532–746)^45,46^, whereas the p.V66E mutation identified in Family 1 is located in the N-terminal homopentamer-forming domain (Fig. 3c). The p.R718W mutation identified in Family 2 is a recurrent COMP mutation associated with MED^47–50^, which corroborates our diagnostic suspicion of MED in Family 2. Whether other reported MED patients carrying p.R718W mutation also exhibited symptoms of CTS is unknown. *COMP* is expressed primarily in cartilage, tendon, and ligament^51^, supporting a pathogenic role for mutant COMP causing defects in tendon/ligament apart from cartilage. However, unlike the COMP mutations found in PSACH or MED, the p.V66E mutation does not appear to affect cartilage growth, as we have not observed any PSACH- or MED-like phenotype in Family 1. In contrast, the p.R718W mutation affects both tendon/ligament and cartilage in Family 2.

### COMP mutations impaired COMP secretion and complex formation

**Fig. 4:**
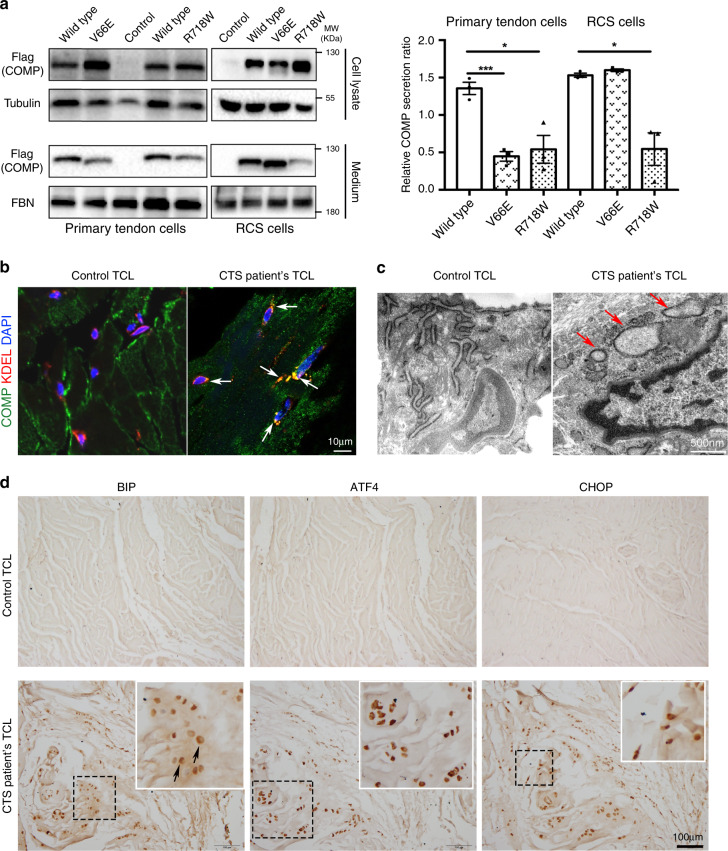
ER retention and activation of ER stress/UPR by mutant COMP. **a** Wild-type, V66E, and R718W mutant COMP were expressed in primary tendon cells or RCS cells. Cell lysates and culture medium were respectively collected. Immunoblotting results indicate poor secretion of V66E mutant COMP by primary tendon cells but not RCS cells. The secretion of R718W-COMP is impaired in both types of cells. RCS rat chondrosarcoma cells, FBN fibronectin. The secretion ratios of different COMP from three experiments are quantified and summarized in the right panel, two-tailed *t* test, ***p = 0.00095 (V66E) and *p = 0.0161 (R718W) for primary tendon cells, *p* = 0.107 (V66E) and *p = 0.0108 (R718W) for RCS cells, error bars are ± SEM. **b**–**d** Normal (*n* = 2) and Family 1 patients’ TCL tissues (*n* = 3) were analyzed. **b** Immunofluorescent staining indicates strong co-localization of COMP (green) and endoplasmic reticulum (ER, recognized by KDEL antibody, red) in patients’ TCL cells. DAPI stains cell nucleus (blue). **c** Transmission electron microscopy (TEM) shows distended and fragmented ER in CTS patients’ TCL cells (red arrows). **d** Immunohistochemical staining shows upregulation of BIP, ATF4, and CHOP in patients’ TCLs, indicating activation of ER stress/unfolded protein response (UPR) in CTS patients. Arrows point to the perinuclear staining of the ER-resident BIP. The boxed areas (black box) in the figures are magnified and shown in the insets (white box). Source data are provided as a Source Data file.

To determine how these mutations affect COMP function, wild-type, V66E, and R718W mutant *COMP* were expressed by adenoviruses in primary tendon cells isolated from young mice. As a control, we also transfected wild-type and mutant *COMP* into rat chondrosarcoma cells (RCS). We found that the secretion of both mutant forms of COMP by primary tendon cells was reduced compared with that of wild-type COMP, while the secretion by RCS cells differed (Fig. 4a). The V66E secretion was as efficient as the wild-type COMP in RCS cells, but the R718W secretion was reduced (Fig. 4a). These results indicate that the V66E and R718W mutations differentially disrupt COMP protein secretion in tenocytes and chondrocytes, which may explain the different phenotypes (CTS vs. CTS/MED). Since the V66E mutation is specific for CTS and it is the only COMP mutation in the coiled-coil domain, our following functional analysis focused on this mutation.

As valine 66 resides in the coiled-coil domain and is a predominant hydrophobic amino acid in the heptad repeat that stabilizes the pentamer structure (Fig. 3d, e), replacement of valine by a negatively charged glutamate is likely to disrupt the coiled-coil domain structure and interfere with pentamerization^52^. To test this possibility, we first examined COMP protein complex formation by native polyacrylamide gel electrophoresis assay (NPAGE). Indeed, a substantial fraction of the V66E-COMP was found in monomers in both primary tendon cell lysates and medium. No wild-type COMP was detected in monomeric form (Supplementary Fig. 8a). To further characterize the oligomerization states of COMP, we purified the coiled-coil domains of wild-type and V66E-COMP and examined cross-linked states of the proteins. Wild-type COMP was present in di-, tri-, tetra-, and pentamers, while V66E-COMP exhibited a prominent monomer band and an additional unknown complex, indicating impaired oligomerization of COMP caused by the V66E mutation (Supplementary Fig. 8b). Furthermore, a sucrose density-gradient ultracentrifugation assay that separates protein complexes by size difference was performed in primary tendon cells (Supplementary Fig. 8c). In the conditioned medium that contained secreted COMP protein, we found that the wild-type COMP protein appeared in heavier fractions than the V66E-COMP. In cell lysates, the V66E-COMP also spread more to the lighter fraction (Supplementary Fig. 8c). It appears that the heavier protein complex containing mutant COMP was not efficiently secreted into the medium (compare boxed areas in lysates and medium in Supplementary Fig. 8a, c). Cultured primary tendon cells formed ECM-like depositions, in which we detected co-localization of wild-type COMP with type I collagen (COLI) (arrows, Supplementary Fig. 8d). When V66E-COMP was expressed in primary tendon cells, COLI appeared to be normally secreted to form ECM-like depositions (arrow heads, Supplementary Fig. 8d), but V66E-COMP was barely found. Taken together, our results indicate that the proper oligomerization of COMP is important for its sufficient secretion in primary tendon cells and its integration into ECM protein complex.

The CTS patients in both pedigrees are heterozygous for COMP mutations; therefore, the effect of mutant COMP over wild-type COMP was determined by co-expressing them in a 1:1 ratio in primary tendon cells. We found that the mutant COMP impaired the secretion of wild-type COMP molecules (Supplementary Fig. 8e). This is not surprising because only 1 out of 32 possible pentamers will be completely normal. When the purified wild-type and V66E-COMP coiled-coil domain proteins were mixed, the percentage of monomer is still nearly as high as V66E-COMP alone (Supplementary Fig. 8b). These results revealed a possible molecular mechanism whereby mutant COMP interferes with normal matrix complex assembly mediated by wild-type COMP, leading to a reduced secretion of all COMP molecules.

### ER stress and UPR in patients’ biopsies

Reduced secretion of mutant COMP in cultured primary tendon cells prompted us to examine whether such reduction was due to increased endoplasmic reticulum (ER) retention of mutant COMP as reported for other mutant ECM proteins^53^. We found strong co-localization of mutant COMP with the ER marker KDEL in TCL cells of Family 1 patients, but such co-localization was hardly observed in control TCL cells (Fig. 4b). Consistently, the extracellular COMP appeared to be reduced in patients’ TCLs (Fig. 4b). ER accumulation of misfolded or unfolded proteins induces ER stress^54^, which triggers a cellular-adaptive mechanism known as the unfolded protein response (UPR). UPR alleviates stress and restores ER homeostasis by attenuating protein translation, producing more molecular chaperones or promoting ER-associated degradation (ERAD) of mutant proteins. Although selective UPR activation can be cytoprotective, chronic UPR activation by prolonged ER stress has deleterious effects such as cell death^53,54^. To determine whether mutant COMP increased ER stress, we analyzed the ultrastructure of human TCL cells by TEM, and found that the ER was distended and fragmented in CTS patients’ TCL cells (Fig. 4c). Expression of BIP, an indicator of ER stress, was also upregulated (Fig. 4d). In addition, we found that the UPR-PERK pathway (one of the three UPR-sensing pathways) was activated in TCL cells of CTS patients, as indicated by upregulation of transcriptional factor ATF4 and its downstream target CHOP, an ER stress-induced apoptosis gene^53,55^ (Fig. 4d). Similar evidence of ER stress was also identified in patients’ flexor tendons and surrounding SSCT (Supplementary Figs. 6c and 7c, d). Then, we found that the number of TCL cells marked by tenomodulin (Tnmd) was significantly reduced in patients, which likely resulted from markedly increased cell death (Supplementary Fig. 9a, b). Consistent with the role of cell death in triggering inflammation and progressive fibrosis^56^, we also identified inflammatory cells in patients’ TCLs (Supplementary Fig. 9c) as well as fibrosis (Fig. 2c, d and Supplementary Fig. 4b, c). Taken together, our findings indicate that mutant COMP is associated with increased ER stress and tendon/ligament cell death, which may trigger inflammation and fibrosis and impair ECM architecture.

### Characterization of a *COMP* knock-in mouse model

To further understand the functional impact of the mutant COMP on CTS in vivo, we generated two mouse mutant lines, *V65E-COMP* knock-in and *COMP*-knockout mice (Supplementary Fig. 10a, b). The p.V65E mouse mutation is equivalent to the p.V66E human mutation. While no COMP protein could be detected in the *COMP*-null mice, the protein levels of the V65E-COMP were reduced (Supplementary Fig. 10c), suggesting that the V65E-COMP protein may have been partially degraded through ERAD.

**Fig. 5:**
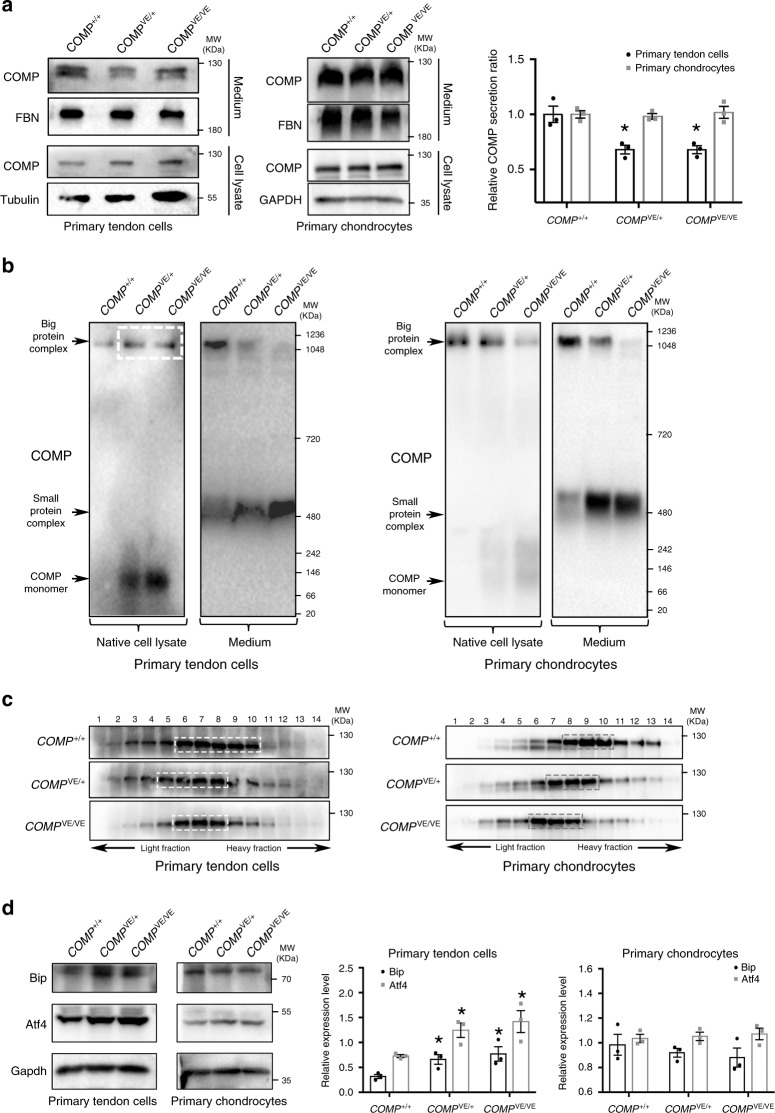
Impaired COMP complex formation and secretion in a *COMP* knock-in mouse model. Primary tendon cells and chondrocytes were isolated from 6- to 8-week-old wild-type and mutant mice and cultured in vitro. Cell lysates and culture medium were respectively collected. **a** Reduced secretion of endogenous mutant COMP by primary tendon cells but not primary chondrocytes. FBN fibronectin. The secretion ratios of different COMP from three experiments are quantified and summarized in the right panel, two-tailed *t* test, *p = 0.0187 (*COMP*^*VE/+*^) and 0.0174 (*COMP*^*VE/VE*^) for primary tendon cells, *p* = 0.688 (*COMP*^*VE/+*^) and 0.795 (*COMP*^*VE/VE*^) for primary chondrocytes, error bars are ± SEM. **b** Native gel electrophoresis of primary tendon cells and chondrocytes reveals significant amounts of monomer or small protein complex in *COMP*^*VE*^ mutants. The major difference between tenocytes and chondrocytes is the retention of a big protein complex (white box) and a monomer in the tendon cell lysates. **c** The sucrose density-gradient ultracentrifugation of culture medium of primary tendon cells and chondrocytes also indicates lower molecular weight of protein complex containing the mutant COMP. **d** Immunoblotting of Bip and Atf4 in primary tendon cells and chondrocytes indicates increased ER stress/UPR in tenocytes. The protein levels from three experiments are quantified in the right panel, two-tailed *t* test, *p = 0.030 (*COMP*^*VE/+*^) and 0.041 (*COMP*^*VE/VE*^) for Bip, *p = 0.0245 (*COMP*^*VE/+*^) and 0.0358 (*COMP*^*VE/VE*^) for Atf4, error bars are ± SEM. Source data are provided as a Source Data file.

We examined long bones of *COMP*^*VE/+*^*, COMP*^*VE/VE*^, and *COMP*^*−*/*−*^ mutant mice by X-ray. Their long bones appeared to be grossly normal (Supplementary Fig. 11a), which is consistent with published observations in another line of *COMP*-null mice^57^ and lack of long bone defects in our Family 1 patients. Although the protein level of COMP was also reduced in *COMP*^*VE/VE*^ growth plates, the secretion of the mutant COMP appeared normal (Supplementary Fig. 11b, c), which was later confirmed by the result of normal secretion of mutant COMP in primary chondrocytes isolated from *COMP*^*VE*^ mice (Fig. 5a). However, the secretion of mutant COMP in primary tendon cells of *COMP*^*VE*^ mice was reduced (Fig. 5a). We analyzed the COMP complex formation in isolated primary tendon cells and chondrocytes by NPAGE and sucrose density-gradient ultracentrifugation assays. NPAGE clearly exhibited COMP monomers in *COMP*^*VE*^ primary tendon cell lysates (Fig. 5b, left). Most of the wild-type COMP existed in a big protein complex in the culture medium, whereas the big protein complex containing mutant COMP was largely trapped in the cell (white box in cell lysate), and the secreted mutant COMP presented as a small protein complex in the medium (Fig. 5b, left). Similarly, a COMP monomer or small protein complex was observed in mutant primary chondrocytes; however, much less retention of the big protein complex or COMP monomer was observed in the chondrocyte lysates (Fig. 5b, right). Sucrose density-gradient ultracentrifugation assays also indicated that the molecular weight of protein complex containing mutant COMP was lower than that of wild type in both primary tendon cells and chondrocytes (Fig. 5c). Therefore, although the oligomerization of COMP was also affected in chondrocytes, the chondrocytes appeared to cope with this change better than tendon cells. The mutation compromised the ability of primary tendon cells to secrete the big COMP-containing protein complex and COMP monomers. Indeed, we detected ER retention of mutant COMP proteins and enlarged ER lumen in tendons of *COMP*^*VE*^ mice (Supplementary Figs. 12a and 14a). The ER stress and UPR markers Bip and Atf4 were upregulated in primary tendon cells but not in primary chondrocytes of *COMP*^*VE*^ mice (Fig. 5d).

**Fig. 6:**
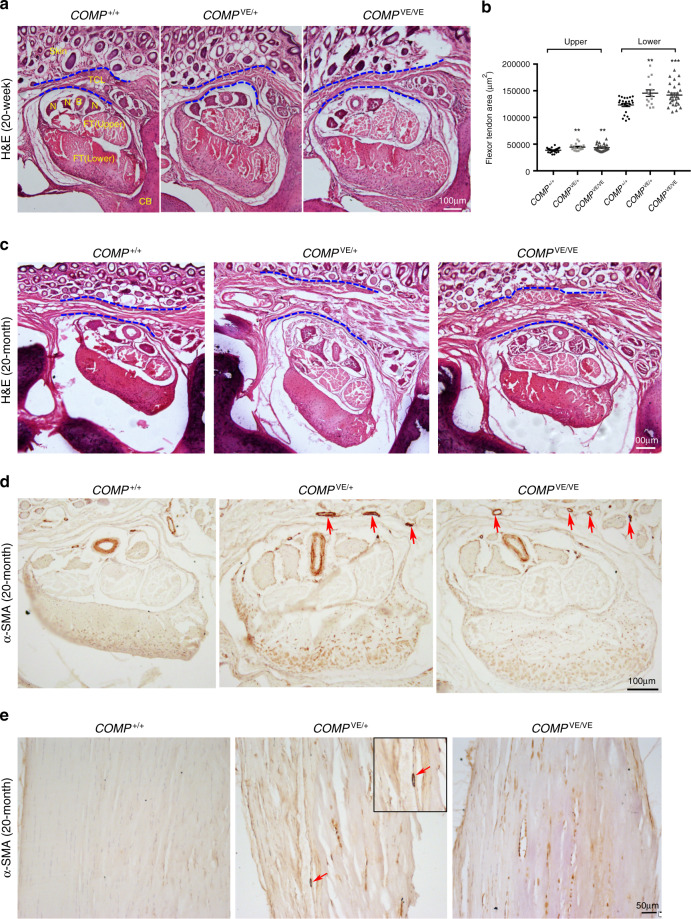
Phenotypic analysis of the CTS mouse model. The *COMP* knock-in mouse model carries the mouse mutation corresponding to human p.V66E (*n* ≥ 3 per genotype for each following analysis). **a** The H&E staining of 20-week-old mouse carpal tunnel shows comparable TCL, which is outlined by blue dotted lines, in wild-type and *COMP* mutants. The flexor tendons tend to be bigger in *COMP* mutants. **b** The statistical results of the cross-sectional area of flexor tendons (upper and lower parts, respectively). In total, 23 sections from three wild-type mice, 15 sections from four *COMP*^*VE/+*^ mice, and 28 sections from four *COMP*^*VE/VE*^ mice were analyzed, two-tailed *t* test, upper **p = 0.002, 0.0034; lower **p = 0.0011, ***p = 0.0007, error bars are ± SEM. TCL transverse carpal ligament, N median nerve, B blood vessel, FT flexor tendon, CB carpal bone. **c** H&E staining of 20-month-old mouse carpal tunnel shows enlarged TCL in *COMP* mutants, which is outlined by blue dotted lines. α-SMA staining in 20-month-old mouse carpal tunnels (**d**) and Achilles tendons (**e**). The fibrosis is increased in *COMP*^*VE*^ mutants. Vascular structures are observed in mutants (red arrows). Source data are provided as a Source Data file.

In mouse carpal tunnels, we also found upregulation of Bip and Atf4 in mutant TCL and flexor tendons (Supplementary Fig. 12b, c), similar to what we found in patients. Mutant mice consistently exhibited increased cell death (Supplementary Fig. 13a), and a few inflammatory cells were detected (Supplementary Fig. 13b). Although we observed similar molecular and cellular changes in *COMP*^*VE*^ mice, it is expected to take a long time for mice to develop a histological phenotype because at least 20–30 years are required for Family 1 patients to show symptoms, and caged mice have limited physical activities. In the 20-week-old *COMP*^*VE/+*^ and *COMP*^*VE/VE*^ mice, we observed slight increases of α-SMA expression (Supplementary Fig. 13c) and normal TCL, while the flexor tendons tended to be bigger (Fig. 6a, b). But in the 20-month-old mutant mice, the thickening of TCL and fibrosis in the carpal tunnel were more evident (Fig. 6c, d). We also observed inflammation and progressive fibrosis in mutant Achilles tendons (Fig. 6e and Supplementary Fig. 14b, c), suggesting that the COMP mutation caused a systemic change in tendon/ligament similar to what we observed in human patients. The tissues of old mutant mice were more fragile than wild-type when being sectioned (Fig. 6e), suggesting a possible change in the ECM. We further analyzed the ECM ultrastructure of mouse Achilles tendons by TEM, and found that the diameter distribution of collagen fibrils was altered in *COMP*^*VE/+*^ and *COMP*^*VE/VE*^ mice (Supplementary Fig. 15). There was an increase in the thinner collagen fibrils in the mutants, and this became more prominent in older mice with appearance of many ectopic small fibrils (red arrows in Supplementary Fig. 15), like the TEM analysis results of patients (Fig. 2e). These data show that the mouse model of the human mutation exhibited similar molecular, cellular, and ECM changes, and the phenotypes were progressively more severe. Such changes were not previously reported in *COMP*-null mice^57^, which we confirm, indicating a dominant effect of the mutation.

### Compromised tendon-healing potential in CTS mouse model

**Fig. 7:**
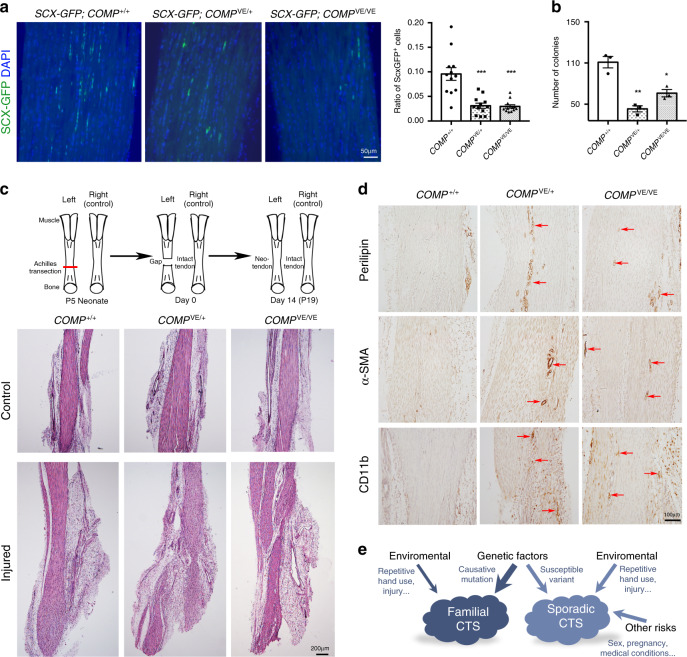
Compromised tendon-healing potential in the CTS mouse model. **a** The number of Scx-positive cells (green) in Achilles tendon is significantly decreased in 30-week-old *COMP*-mutant mice (*n* = 3 per genotype, 12 slides per genotype were analyzed, two-tailed *t* test, ***p = 0.0001 (*COMP*^*VE/+*^) and 6.22 × 10^−5^ (*COMP*^*VE/VE*^), error bars are ± SEM). **b** Primary tendon cells were isolated from 4-week-old Achilles tendons and cultured in vitro. Colony assay of isolated tendon cells shows fewer colonies formed by *COMP*-mutant mice (*n* = 3 per genotype, two-tailed *t* test, *p = 0.0107, **p = 0.002, error bars are ± SEM). See Supplementary Fig. 16a for representative pictures. **c** Schematic and results of Achilles tendon transection injury experiments on neonatal mice (*n* ≥ 5 per genotype). Left Achilles tendon was transected at P5 (postnatal 5 days) and the right side was kept intact as a control. Tendon tissues were collected at P19 for analysis. H&E staining (lower panel) shows disorganization and poor recovery of injured Achilles tendons in mutant mice. **d** Immunohistochemical staining of injured Achilles tendons at P19 indicates increases of adipocytes (perilipin), fibrosis (α-SMA), and inflammation (CD11b) in mutant mice. **e** Schematic showing the contribution of different factors to the development of CTS, either familial or sporadic. The width of the arrow reflects the approximate extent of the contribution. Source data are provided as a Source Data file.

As tendon stem/progenitor cells (TSPCs) are crucial for repair and regeneration of damaged tendon tissues^58–60^, we first investigated the potential TSPCs in animal models and found that the number of *Scleraxis* (*Scx*)-positive cells was reduced in the *COMP*^*VE/+*^ and *COMP*^*VE/VE*^ mutant Achilles tendons (Fig. 7a). We characterized clonogenicity of Achilles tendon cells isolated from wild-type and mutant mice, and found that the number of colonies formed by the *COMP*^*VE/+*^ and *COMP*^*VE/VE*^ mice was lower than the wild-type ones (Fig. 7b, Supplementary Fig. 16a). But in *COMP*-null mice, a comparable number of colonies was formed (Supplementary Fig. 17a), suggesting that COMP is not an essential niche factor for maintaining TSPCs. More likely, the dominant effects caused by the COMP mutation lead to reduction of TSPCs. Interestingly, we further found that the expression of fibromodulin (Fmod), an ECM niche component in maintaining TSPCs^59^, was downregulated in *COMP*^*VE/+*^ and *COMP*^*VE/VE*^ mice (Supplementary Fig. 16b, c). Since the ultrastructure of collagen fibrils was altered in the mutants, the COMP mutation likely resulted in an overall change of ECM environment, which may contribute to the defects of TSPCs and the phenotype.

We reasoned that the ability of *COMP*-mutant mice to repair tendon damage might be compromised. To test this, we employed a model of tendon regeneration using neonatal mice, in which transected Achilles tendons could heal via formation of a neotendon and restore Achilles tendon function without scarring^61^ (Fig. 7c). This tendon-specific differentiation process in injured neonates contrasts to the ineffective healing process in injured adults^61^. Wild-type neonates recovered from tendon injury subsequently exhibited well-organized neotendons and nearly no adipocytes or α-SMA-positive cells in the tendon proper (Fig. 7c, d). However, similar injuries on *COMP*^*VE/+*^ and *COMP*^*VE/VE*^ mice resulted in disorganized neotendon formation and appearance of adipocytes and α-SMA-positive cells (Fig. 7c, d). Unlike the wild-type neonates, in which nearly no inflammatory cells could be found after healing, *COMP*^*VE/+*^ and *COMP*^*VE/VE*^ neonates still showed obvious inflammation (Fig. 7d). Because the tendon healing of *COMP*^−/−^ neonates was comparable to that of wild-type ones (Supplementary Fig. 17b, c), these results suggest that the COMP mutation is dominant-negative and compromises the regenerative capability of tendons.

## Discussion

We identified two missense mutations in *COMP* as the basis of familial CTS, a progressive, painful condition caused by nerve compression in the wrist. The p.V66E mutation in the N-terminal coiled-coil domain is specific for CTS, unlike mutations previously associated with PSACH or MED (Fig. 3c). Patients carrying the p.V66E mutation (Family 1) exhibit no PSACH- or MED-like phenotype. The p.R718W mutation in Family 2 causes both CTS and MED. Our data suggest that cell-type-specific regulation of mutant COMP secretion explains tissue-specific effects of COMP mutations affecting distinct domains. The p.V66E substitution imparts a pathogenic change by disrupting COMP pentamerization; mutant COMP protein complexes are poorly secreted by tendon/ligament cells while the chondrocyte secretion is unchanged. The secretion of R718W-COMP is affected in both tenocytes and chondrocytes. The mechanism regulating cell-type-specific secretion of V66E-COMP is unknown, probably because the interacting proteins of COMP in different cell types are different or simply due to distinct expression of specialized ER chaperones in different cell types. Misfolded or unfolded ECM proteins are often secreted poorly and retained within the ER, inducing ER stress-mediated UPR. This mechanism has been well established in several chondrodysplasias^53^. While PSACH-causing mutant COMP is known to be accumulated within chondrocytes^46,62^, the CTS-specific mutant COMP (p.V66E) is only accumulated in tendon/ligament cells but not chondrocytes. Both accumulations induce UPR and subsequent cell death. Our studies of the p.V66E mutation demonstrate ER stress as a causal mechanism in a tendon/ligament-related disease.

Different mutations in the same gene can result in different phenotypes, and the general term “COMPopathies” was suggested to encompass the clinical variability of MED and PSACH^62^. Our findings add a distinct manifestation to the COMPopathies spectrum; PSACH and CTS are on the two ends, and MED is an intermediate phenotype. We carefully excluded MED or PSACH in our Family 1 patients. There are no previous reports of CTS associated with MED or PSACH. The co-existence of CTS and MED in Family 2 indicates that CTS might be associated with MED or PSACH. Previously identified MED or PSACH patients might also have CTS symptoms that have escaped proper diagnosis due to mild manifestation or late age of CTS onset. The Family 2 onset of CTS varies between 30 and 50 s, but MED and PSACH are childhood-onset diseases. Importantly, CTS cannot be diagnosed from radiographs used to assess MED/PSACH; a hand specialist is required for CTS diagnosis. We examined three additional PSACH patients whose disease is caused by a published recurrent PSACH-COMP mutation p.469insD^63^. Our clinical reexaminations showed that two of them exhibited mild CTS at ages of 49 and 56 and one did not, possibly due to his younger age of 30 (Supplementary Table 1). To identify a statistically significant association and to clarify the variability in age of CTS onset would require detailed investigations of more adult MED and PSACH patients.

A recent GWAS study of sporadic CTS identified variants within genes involved in skeletal growth and ECM, and suggested a contribution of ECM environment to CTS susceptibility^32^. However, the causal relationship and the underlying mechanism remain unknown. Studies of rare Mendelian forms of common diseases have proven invaluable in identifying disease genes, and in elucidating pathogenic mechanisms by identifying molecular pathways underlying complex traits^64^. Here, the identification of causative mutations in COMP in familial patients revealed roles of ECM proteins in CTS pathogenesis, supporting the GWAS findings. Our functional studies in patients’ tissues and animal models suggest that the induced cellular stress response and change in ECM organization may be critical in CTS pathogenesis. The ectopic ECM depositions (those darkly stained materials in TEM) found in our familial patients are likely a mixture of ectopic collagen fibrils (e.g., type III collagen) secreted by fibrotic cells and misfolded COMP deposits left behind by apoptotic cells. Although many misfolded COMP molecules are trapped in the ER, a limited amount of misfolded COMP can still be secreted and may also contribute to the formation of ectopic ECM. It will be interesting to determine in the future whether there is an enrichment of ECM gene mutations or an alteration of ECM organization in other CTS patients, either familial or sporadic.

As key tissues responsible for body movement by transmitting force from muscle to bone or bone to bone, tendons and ligaments often accumulate exercise- and age-related cellular and ECM microdamage, which can normally be repaired by mature tendon/ligament cells and/or stem/progenitor cells^59,65,66^. If tendons/ligaments are overused, tendinosis, a progressive degeneration process with little inflammation, ensues. If, instead, acute injury happens, inflammation of tendons/ligaments will cause the condition of tendinitis. While it is well known that chronic inflammation induces fibrosis^67^, tendinosis can also promote fibrosis and thickening of the tendon without histologic evidence of inflammation^68^. Since typical CTS onset is after age 45, tendinosis may progressively cause CTS in the general population. This is consistent with limited inflammation observed in many CTS patients’ tissues^8,35^. If inflammation is induced, the fibrosis may be accelerated, and CTS may present earlier or in a more severe manner. In our Family 1 CTS patients, the earlier age of onset is likely a consequence of the mutation-induced cellular stress response. The COMP mutation induced ER stress/UPR and cell death, which trigger inflammation and progressive fibrosis^56^. Due to the decrease in TSPCs, the tendon/ligament repair was insufficient, and the ECM architecture was disrupted. UPR and altered ECM niche may also contribute to cell fate change by “reprogramming” stem/progenitor cells^54,59,69^, leading to an accumulation of fibrotic and adipogenic cells, as well as formation of neovascular structures. Eventually, fibrotic connective tissues in the human carpal tunnel become thickened and swollen, thus compressing the median nerve in causing CTS.

The age of onset varies from less than 20 to more than 40 even within Family 1, yet all patients carry the same mutation. This may reflect differential contributions of other genetic polymorphisms and/or environmental factors. For example, hand overuse or acute injury may accelerate CTS onset. Indeed, in our CTS mouse model, acute injury accelerated and enhanced the phenotype. The change of tendons/ligaments in CTS patients was systemic as we also observed bigger flexor and Achilles tendons in Family 1 patients and similar changes in flexor and Achilles tendons of our CTS mouse model. CTS in wrists may represent an early manifestation of tendons/ligament thickening and swelling, while similar changes in other body parts may not cause remarkable symptoms if no nerves are compressed. In CTS/MED patients of Family 2, the tendon/ligament changes are less significant. This might be due to less hand usage consequent to their MED-associated joint pain, and may explain the later CTS onset in Family 2. The progressive mechanism of CTS explains how a congenital condition manifests in adulthood. The adult age of onset allows time for therapeutic intervention in young carriers of genetic mutations or individuals with other conditions that activate the same signaling events. Our study suggests that intrinsic ER stress and potentially, additional cellular stresses such as hypoxia stress, oxidative stress, and mechanical force^70^ (induced by either genetic mutations or environmental conditions), may be a recurrent pathogenic mechanism for CTS. Our findings and the recent GWAS study suggest that, in addition to the environmental and known risk factors, genetic mutations and variants also influence CTS in the general population (Fig. 7e).

In summary, this study revealed a pathogenic mechanism underlying CTS, and highlighted the importance of genetic mutations in ECM genes in causing CTS. Mutant COMP leads to abnormal activities in causing cell death and disrupting the ECM niche in tendons and ligaments, which diminishes their capacity to repair tendon/ligament damage. Without sufficient repair activity and with continuous inflammation, fibrotic and other types of cells accumulate, and ECM composition is changed, resulting in swollen and thickened soft connective tissues, which compress the neighboring structures. The carpal tunnel is a narrow and closed space, so the compression of the median nerve becomes pronounced there and causes CTS. Our findings suggest that cellular stress response and alteration of matrix deposition and assembly could cause predisposition to CTS in the general population. Sporadic and idiopathic CTS, particularly with early onset and/or bilateral symptoms, may result from combined effects of genetic susceptibility and environmental factors (Fig. 7e). Interestingly, a quantitative analysis of published scientific evidence regarding the etiology of CTS found that the major risk factors for CTS are more likely to be genetic and biological than environmental or occupational^71^. Therefore, our findings provide a foundation for further investigation of the genetic and molecular bases of CTS.

## Methods

### Study participants

Two families with bilateral CTS were recruited for this study from China-Japan Union Hospital of Jilin University. The probands of both families were initially identified when they underwent carpal tunnel release surgeries. In Family 1, we studied 27 affected members from two branches of the same family, as well as 25 unaffected members (labeled with numbers in Fig. 1a and Supplementary Fig. 1). In Family 2, we studied 11 affected and 5 unaffected members (labeled with numbers in Fig. 1a). All CTS patients were diagnosed by experienced hand surgeons; some of the patients and controls were further examined by both hand surgeons and orthopedists with more detailed measurements (Fig. 1b, c, Supplementary Figs. 2, 3 and Supplementary Table 1). Three PSACH patients were examined by hand surgeons in the First Affiliated Hospital of Sun Yat-sen University. Written informed consents from all the participants in the study were obtained. The study was approved by the ethics committees of the China-Japan Union Hospital of Jilin University, China, the National Human Genome Research Institute, National Institutes of Health (NIH), USA, and the University of Hong Kong, Hong Kong SAR, China.

### Genotyping and analysis of linkage

DNA samples from 52 members of Family 1 and 16 members of Family 2 were available for genetic studies. Genome-wide analysis of linkage on 17 affected and 14 unaffected members of Family 1 was performed using genotypes from the CytoSNP chip (Illumina) that has 246,691 SNP markers. After linkage was established to a segment of chromosome 19p12-p13.11, 18 concentrated microsatellite markers (Supplementary Table 2) were genotyped on the same 31 individuals and an additional eight individuals. Individuals younger than 20 were considered to have unknown (0) affection status. LOD score analysis was done mostly with Superlink-SNP^72^. Analysis of small sets of markers was done with command-line Superlink^73^ and FASTLINK^74–76^. The single-marker analysis of Superlink-SNP filters the input marker set to at most 25,000 markers. For multimarker analyses of SNPs, the SNPs selected by Superlink-SNP were used. The disease was modeled as autosomal-dominant, and the disease allele frequency was set to 0.01.

### Sequencing

Six affected and six unaffected members of Family 1 were first screened for mutations by means of targeted sequencing. The targeted interval was captured with the use of a NimbleGen custom sequence capture array and then sequenced with an Illumina HiSeq2000 sequencer. The resulting sequence data were processed by a custom analysis program, MPG (Most Probable Genotype), using a probabilistic Bayesian algorithm^77^, at NISC (NIH Intramural Sequencing Center). Varsifter, a java-based genotype viewer^78^, was used to filter single-nucleotide and indel (insertion and deletion) variants on the basis of effects of variants on amino acid conservation and probability of protein damage, pedigree structure, and published databases. The common variants reported in reference genomes were excluded. The following databases were used: dbSNP144, the 1000 Genome Project, the National Heart, Lung, Blood Institute (NHLBI) exome variant server (EVS) database, the Exome Aggregation Consortium (ExAC) dataset, and the Genome Aggregation Database (gnomAD). The mutation of Family 2 was directly detected by standard whole-exome sequencing, and the same procedure of sequencing data analysis was followed. Both mutations were verified by Sanger sequencing in all participants.

### Magnetic resonance imaging (MRI)

MRI was performed on a 3.0T MRI scanner (Siemens Skyra VE11) and analyzed by 3D MPR (multiplanar reconstruction). To locate the comparable axial images of controls’ and CTS patients’ carpal tunnels in MRI datasets, the hook of the hamate bone was used as a reference position. The horizontal cross-sectional areas of carpal tunnels, flexor tendons, and median nerves were analyzed and measured by the MR scanner platform software (SIEMENS-VE11) and RadiAnt DICOM Viewer (SIEMENS-VE40B). Sagittal image of TCL was also identified based on the hook of the hamate bone, and the thickness of TCLs was measured at the carpometacarpal level. The axial image at the vertex of the medial malleolus was used to measure the horizontal cross-sectional area of the Achilles tendon.

### Joint-flexibility measurement

The joint flexibility was measured using standard ROM (range of motion) method. To obtain the number of degrees from the starting position of a segment to its position at the end of its full range of the movement, a standard goniometer was placed across the joint, and visual estimation was done by two experienced clinical evaluators. The participants were asked to perform the following movements: passive apposition of the thumb to the flexor aspects of the forearm, passive dorsiflexion of the fifth finger, flexion and extension of the wrist, and flexion and dorsiflexion of the ankle.

### Plasmids, adenoviruses, and antibodies

A plasmid containing the human COMP full-length coding sequence was a gift from Jack Lawler (Addgene plasmid #13002). A plasmid containing the His-tagged rat COMP coiled-coil domain sequence (COMPcc) was a gift from Jin Kim Montclare. The human COMP full-length coding sequence was cloned into the pIRES-hrGFP vector (Agilent, hrGFP had been removed), and a Flag or HA tag was fused to its C terminus. Mutations (p.V66E and p.R718W) were introduced by site-directed mutagenesis. Based on these constructs, adenoviral vectors expressing wild type or mutant COMP were custom-made by Applied Biological Materials (ABM) Inc. Adenoviruses were produced and amplified in HEK293 cells and then subjected to infecting primary tendon cells. Antibodies used in this project are described in each section of “Methods”. Recombinant mouse Tgfβ2 protein was purchased from R&D (7346-B2-005).

### Culture of primary tendon cells and chondrocytes

Mouse Achilles tendons were harvested from 4- to 8-week-old mice. Following the protocol previously published^59^, the tendon sheath and the surrounding paratenon were stripped off, and tendon tissues were cut into small pieces and digested with collagenase type I, dispase, and elastase in phosphate-buffered saline (PBS). Mouse costal cartilage was isolated from 4-week-old mice and digested with collagenase type II and dispase II in HBSS. Single-cell suspensions were cultured in Dulbecco’s modified eagle medium supplemented with 10% fetal bovine serum (FBS), and then subjected to adenoviral infection of COMP (if required) and cultured for 6–9 days before collecting medium and cell lysates for Western blot analysis or processing for immunofluorescent staining. To maintain the identity of tendon cells, 20 ng/μl mTgfβ2 was added into the medium every 3 days.

### Native gel electrophoresis

Non-SDS detergent digitonin was used to lyse cell and solubilize proteins. The proteins in medium were concentrated by Amicon Ultra-15 Centrifugal Filter Unit with Ultracel-30 membrane (Millipore). The native gel electrophoresis was performed using NativePAGE Bis-Tris 3–12% Gels (ThermoFisher Scientific) with NativeMark Unstained Protein Standard (ThermoFisher Scientific) as a protein-size marker.

### Density-gradient sedimentation

The concentrated medium or native cell lysates were transferred on top of a premade 15–40% graded sucrose buffer (30 mM Tris (pH 7.4), 140 mM NaCl, and protease inhibitors) followed by ultracentrifugation at 40,000 rpm (151,000 × *g*) for 16 h at 4 °C (Beckman Coulter Optima L-90K using SW55Ti rotor). Fractions were collected from the top to bottom of the centrifuge tube and analyzed by western blot.

### Protein purification and cross-linking assay

Wild-type and mutant (V66E) COMPcc plasmids were transformed into XL1 blue cells. The previously used procedures of protein expression and purification were followed^79^. In all, 1/100 dilution of the starter cultures were grown in 1 L LB medium (1 mM ampicillin) at 220 rpm (120 × *g*) at 37 °C. When OD_600_ is ~1.0, 1 mM isopropyl β-d-thiogalactopyranoside (IPTG) was added, and protein expression was induced at 37 °C for 3 h. Cells were pelleted, resuspended with equilibration buffer (0.1 M sodium phosphate monobasic, 10 mM Tris, and 8 M urea, pH 8.0), and then sonicated, after which supernatant-containing target proteins were purified using Ni-NTA agarose affinity resin on the column (Qiagen). Proteins were eluted using 0.1 M sodium phosphate monobasic, 10 mM Tris, and 8 M urea, pH 5.12. Purified proteins were refolded using dialysis buffer I (100 mM phosphate buffer, pH 8.0, 6 M urea) at 4 °C for 3 h, dialysis buffer II (100 mM phosphate buffer, pH 8.0, 4 M urea) at 4 °C for another 3 h, and finally 100 mM phosphate buffer, pH 8.0, at 4 °C for 3 × 3 h. In all, 160 μg of proteins of wild-type alone, mutant alone, and a mixture of 50% wild-type and 50% mutant was used for the chemical cross-linking assay^79^. Proteins were incubated with 0.75 mM BS3 (bis(sulfosuccinimidyl) suberate, ThermoFisher Scientific) at the room temperature for 1 h, and quenched with 20 mM Tris (pH 7.5) at the room temperature for 15 min. BS3 contains an amine-reactive *N*-hydroxysulfosuccinimide ester at each end of an 8-carbon spacer arm, which can react with primary amines to form stable amide bonds. BS3 is used to “Fix” protein interaction to allow identification of interactions such as in vitro oligomerization of COMP. Equal sample amounts were loaded onto a 12% SDS-PAGE gel with nonreducing loading buffer for electrophoresis.

### Western blot assay

Cultured cells, mouse tendon, or cartilage tissues were homogenized and lysed in the lysis buffer (20 mM Tris-HCl [pH 7.4], 150 mM NaCl, and 1% Nonidet P-40) containing protease inhibitors on ice. Protein samples were loaded onto 10% Tris–Glycine gel and transferred to polyvinylidene difluoride membranes, which were then blocked with 5% skim milk and incubated with anti-HA (1:2000, 11867423001, Roche), anti-Flag (1:5000, F1804, Sigma), anti-COMP (1:2000, GTX14515, GeneTex), anti-FBN (1:2000, Ab2413, Abcam), anti-Fmod (1:2000, LF150, provided by Larry Fisher, NIH), anti-ATF4 (1:500, sc-200, Santa Cruz), anti-BIP (1:1000, ADI-SPA-826, Enzo Life Sciences), anti-alpha-tubulin (1:10000, ab7291, Abcam), or anti-GAPDH (1:2000, G8795, Sigma) antibodies. HRP-conjugated rat, rabbit, or mouse secondary antibodies (1:10000, Sigma and GE Healthcare) and Immobilon Western Chemiluminescent Horseradish Peroxidase (HRP) Substrate (Millipore) were used to detect Western blot signals. Uncropped version of immunoblots is shown in Supplementary Information.

### Mouse models

The *Scx-GFP* mouse was provided by Ronen Schweitzer (Oregon Health & Science University)^60^. The Valine at position 66 in human COMP corresponds to a Valine at position 65 in wild-type mouse COMP. The *COMP*-mutant mice carrying the V65E mutation and the *COMP*-null mice were generated by the CRISPR/Cas9-mediated genome editing. To introduce the mouse V65E mutation, a single-guide RNA (sgRNA) and the synthesized single-strand oligo containing the missense mutation T194A were injected into the pronuclei of mouse zygotes with Cas9 mRNA (TriLink BioTechnologies). The founder mice were identified by DNA sequencing and the *COMP* genotypes were confirmed by cDNA sequencing. In total, 13/48 pups were positive, including three carrying homozygous mutation. To knock out *COMP*, two sgRNAs were injected to target Exon1 and Exon2, respectively. The deletions of 3′ end of Exon1 to 3′ end of Exon2 in founder mice were identified by PCR genotyping. In total, 11/31 pups were positive. Western blot was used to verify the *COMP* knockout (Supplementary Fig. 10). The founder mice (most of them were chimeras) were mated to wild-type mice to generate heterozygous mutant mice, which were further intercrossed to produce homozygous mutant mice. The sgRNA for *COMP* V65E mutant: TGCTTGCGGTGAGCATAGAC. The two sgRNAs for *COMP* null: CTGGCTATCCTGCGGGCGAC and GAGAGAGCTGTTGCGACAGC. THE SINGLE STRAND OLIGO: ACCGAGAGGAGGCGCATGGGAAGGGGTCCCGCTGACTTCCTGCCCGGCAGGTCAAGGAGATCACCTTCCTGAAGAATACGGAGATGGAATGTGATGCTTGCGGTGAGCATAGACCCGACCCAAGGGCAGGAGGAAGGGTGGGGGCACAGAGCGAAACGGAGGAAAAGGGCT. Genotyping method: mCOMP-WT-F (GATCACCTTCCTGAAGAATACGGT), mCOMP-V65E-F (GATCACCTTCCTGAAGAATACGGA), and mCOMP-R (CAGCCCTGTCGTTGGTCTTC) were used to genotype the *Comp* V65E allele. mCOMP-F1 (CTCTCCTCAGACCCCACCATATA), mCOMP-F2 (CTTAGTAGGGGACATGGTTGGAC), and mCOMP-R1 (CCCTATCTCTGACTCTGTGCATC) were used to genotype the *COMP*-null allele. Mice were maintained according to the approved protocol of the Committee on the Use of Live Animals in Teaching and Research (CULATR), The University of Hong Kong.

### Histological and immunohistological analyses

Human TCL, flexor tendons, and SSCT collected from three Family 1 patients during the surgeries were fixed in ice-cold 4% formaldehyde solution and embedded in paraffin. The control samples were collected from two age-matched non-CTS patients with limb amputation. Full mouse upper limbs were skinned and fixed in ice-cold 4% formaldehyde solution, decalcified in 0.5 M EDTA, pH 8.0, for 1 week, embedded in paraffin, and sectioned. Mouse Achilles tendons were fixed in ice-cold 4% formaldehyde solution in PBS solution for immunohistochemistry and immunofluorescent staining, embedded in either paraffin or optimal cutting temperature (OCT) compound. Hemotoxylin and eosin were used for H&E staining, and Congo red solution and hematoxylin were used to stain amyloid deposits. For immunohistochemical analysis, slides were deparaffinized and rehydrated; endogenous peroxidase activity was quenched in 3% H_2_O_2_, followed by antigen retrieval with 10 mM citrate buffer (pH 6.0) for 15 min. Hyaluronidase was applied if additional antigen retrieval is required. Samples were blocked in 3% BSA in PBS for 1 h at the room temperature and incubated with primary antibodies, including alpha-SMA (1:200, ab5694, Abcam), CD11b (1:3000, ab133357, Abcam), CD68 (1:400, ab955, Abcam), neutrophil elastase (1:1000, ab68672, Abcam), BIP (1:100, ADI-SPA-826, Enzo Life Sciences), ATF4 (1:100, Sigma), CHOP (1:50, sc-575, Santa Cruz), Perilipin (1:300, 9349 S, Cell Signaling), type III collagen (1:500, ab7778, Abcam), FSP1 (1:200, ab41532, Abcam), 12/101 (1:50, DSHB), COMP (1:500, GTX14515, GeneTex), and Fmod (1:300, LF150, provided by Larry Fisher, NIH) overnight at 4 °C. Sections were then incubated with HRP-conjugated secondary antibodies (anti-rabbit, 1:200, P0260; anti-mouse, 1:200, P0161, Dako) for 1 h at the room temperature and counterstained with hematoxylin.

### Immunofluorescent staining

For immunofluorescent staining, cells fixed in chamber slides or sectioned tissues were permeabilized with 0.5% Triton X-100 in PBS for 5 min, blocked in 3% bovine serum albumin in 0.1% Triton X-100/PBS for 1 h, and incubated with primary antibodies overnight at 4 °C. Primary antibodies used were the following: HA (1:500, 11867423001, Roche), COMP (1:500, GTX14515, GeneTex), type I Collagen (1:500, ab34710, Abcam), cleaved caspase 3 (1:100, 9661 L, Cell Signaling), KDEL (1:200, ab12223, Abcam), Perilipin (1:300, 9349 S, Cell Signaling), alpha-SMA (1:200, C6198, Sigma), CD34 (1:200, ab81289, Abcam), and Tnmd (1:100, ab203676, Abcam). Secondary antibodies coupled to Alexa Fluor 488 or 568 (1:500, Life Technologies) were incubated at the room temperature for 1 h. Images were acquired by a Nikon fluorescent microscope or a Zeiss LSM710 confocal microscope.

### X-ray

After mice were sacrificed, the whole-leg X-ray irradiation of both wild-type and mutant mice was performed in a Varian linear accelerator 6 MV X-ray machine.

### Transmission electron microscopy (TEM)

Human TCLs, flexor tendons, or SSCT tissues collected from surgeries and mouse Achilles tendons were immediately fixed in 2.5% glutaraldehyde in 0.1 M sodium cacodylate buffer for 2 h at 4 °C. The tissues were washed three times in 0.1 M sodium cacodylate buffer and postfixed in 1% OsO_4_ in 0.1 M cacodylate buffer for 2 h. The samples were then dehydrated in increasing concentrations of ethanol and propylene oxide, infiltrated in 1:1 solution of epoxy resin:propylene oxide for 1.5 h at 37 °C, and embedded in epoxy resin overnight at 60 °C. Thin 100-nm sections were cut with a diamond knife (Diatome), placed on copper microscope grids, and then stained with silver citrate solution. Control and patient samples were always processed at the same time, and TEM experiment was repeated four times to avoid potential stain-penetration problem. Sections were scanned by a Philips CM100 transmission electron microscope. Diameters of more than 1500 fibrils were manually measured for each genotype, and the distribution was analyzed for statistical significance using Mann–Whitney *U* tests and plotted by Prism.

### IMMUNO-TEM

Patients’ TCLs were dissected and fixed in 4% PFA in 0.1 M PBS. Tissues were dehydrated in graded ethanol (30%, 50%, 70%, and 90%, and absolute ethanol for 30 min each) and infiltrated in LR White/ethanol mixture (1:1) overnight at the room temperature. Samples were further infiltrated in pure LR White and embedded in plastic capsules with fresh LR white overnight at 60 °C. The ultrathin sections (100 nm) were prepared and transferred to nickel grids. Sections were treated with 10% H_2_O_2_, blocked with 3% bovine serum albumin (BSA) in PBS, and incubated with primary antibodies (anti-type I collagen, 1:10, ab34710; anti-type III collagen, 1:10, ab7778; anti-COMP, 1:10, GTX14515) overnight at 4 °C. After washing with 0.2% BSA/PBS, sections were incubated with “biotinylated” secondary anti-Rabbit antibody (1:50, E0432, Dako) and treated with 10-nm STP-gold (Ab81369, Abcam) in 1% BSA/PBS. They were counterstained with uranyl acetate and lead citrate. Images were captured by Philips CM100 transmission electron microscope. One limitation of our immuno-TEM experiment is that we were not able to obtain additional control samples (that need to be age-matched and fresh) to compare the amount of different ECM proteins between patients and controls.

### Colony assay

Following the previously published procedure^59^, primary tendon cells were isolated from 4-week-old *COMP*-mutant and their littermate control mice and cultured in α-MEM (20% FBS and 100 mM 2-mercaptoethanol, Gibco) at 5% CO_2_, 37 °C. In total, 70–90% confluent cells were resuspended, and exactly 1000 cells were seeded into a T-25 flask. They were continually cultured for 7 days prior to removal of culture medium. Fix/staining solution (0.05% crystal violet) was added into the flasks at the room temperature. After 20 min of staining, the flasks were washed with running water followed by air-drying. Colonies were quantified by Image J.

### Injury assay

Following the previously published procedure^61^, Achilles tendon injury experiments were performed on *COMP*-mutant and their littermate control neonatal pups (P5). The left posterior limb was cleaned by ethanol, and a small skin incision was made to expose the Achilles tendon. The left Achilles tendons were cut transversely in the middle; then the skin was cleaned and closed. The right side was kept intact as controls. After 14 days of recovery, mice were sacrificed and analyzed at P19.

### Statistics and reproducibility

The Mann–Whitney *U* tests were done in R with two-sided *p* values to measure the distribution difference of collagen fibril diameters. Two-tailed Student’s *t* tests were performed in Prism to determine if two sets of data are significantly different from each other. Statistical tests are stated in the figure legends. *P* values < 0.05 were considered as statistically significant. Exact *p* values are provided in figure legends. Continuous variables were expressed as the mean ± SEM as indicated in the figure legends. Unless otherwise stated, the experiments were performed at least three times with similar results, and all representative images reflect a minimum of three biological replicates. Each gel/blot is quantified three times, and the average values from multiple gels/blot are used for statistical analysis.

### Reporting summary

Further information on research design is available in the Nature Research Reporting Summary linked to this article.

## Supplementary information

Supplementary Information

Reporting Summary

## Data Availability

Correspondence and requests for materials related to this study should be sent to gaobo@hku.hk. All data supporting the findings of this study are available within the paper and its supplementary information files. Genotyping and sequencing data are provided as a Source Data file and are available for access upon reasonable request. URLs for online resources referenced in the paper can be found at dbSNP144, http:/www.ncbi.nlm.nih.gov/projects/SNP/; the 1000 Genome Project, http://www.1000genomes.org/; the National Heart, Lung, Blood Institute (NHLBI) exome variant server (EVS) database, http://evs.gs.washington.edu/EVS/; the Exome Aggregation Consortium (ExAC) dataset, http://exac.broadinstitute.org/; the Genome Aggregation Database (gnomAD), http://gnomad.broadinstitute.org. The source data underlying Figs. 1b, c, 2a–e, 4a–d, 5a, d, 6a–e, and 7a–d and Supplementary Figs 2b, c, 3a–f, 4a–c, 5, 6a–c, 7a–d, 8b, e, 9a–c, 10c, 11a–c, 12a–c, 13a–c, 14a–c, 15, 16b, c and 17a–c are provided as a Source Data file. All relevant data are available from the authors on reasonable request. A reporting summary for this article is available as supplementary information file. Source data are provided with this paper.

## Source data

Source Data
