# Author Correction: Mutations in *COMP* cause familial carpal tunnel syndrome

**DOI:** 10.1038/s41467-020-17845-7

**Published:** 2020-08-03

**Authors:** Chunyu Li, Ni Wang, Alejandro A. Schäffer, Xilin Liu, Zhuo Zhao, Gene Elliott, Lisa Garrett, Nga Ting Choi, Yueshu Wang, Yufa Wang, Cheng Wang, Jin Wang, Danny Chan, Peiqiang Su, Shusen Cui, Yingzi Yang, Bo Gao

**Affiliations:** 1 Department of Hand Surgery, China-Japan Union Hospital of Jilin University, Changchun, China; 2 School of Biomedical Sciences, Li Ka Shing Faculty of Medicine, The University of Hong Kong, Hong Kong, China; 3 National Center for Biotechnology Information and National Cancer Institute, National Institutes of Health, Bethesda, MD US; 4 National Human Genome Research Institute, National Institutes of Health, Bethesda, MD US; 5 Department of Orthopaedic Surgery, First Affiliated Hospital of Sun Yat-sen University, Guangzhou, China; 6 Department of Developmental Biology, Harvard School of Dental Medicine, Harvard Stem Cell Institute, Harvard University, Boston, MA US

**Keywords:** Mechanisms of disease, Disease genetics, Mutation, Genetics research, Disease genetics

Correction to: *Nature Communications*10.1038/s41467-020-17378-z, published online 20 July 2020.

The original version of this Article contained an error in the author affiliations.

The affiliation of Bo Gao with National Human Genome Research Institute, National Institutes of Health, Bethesda, MD, USA was inadvertently omitted.

This has now been corrected in both the PDF and HTML versions of the Article.

