# Supplementary material for: Mutations in COMP cause familial carpal tunnel syndrome
Source: Nat Commun. 2020 Jul 20;11:3642. doi: 10.1038/s41467-020-17378-z (PMC7371736; doi:10.1038/s41467-020-17378-z)

Mutations in *COMP* cause familial carpal tunnel syndrome

Li *et al*.

Supplementary Figure S1-S17

Supplementary Table S1, S2

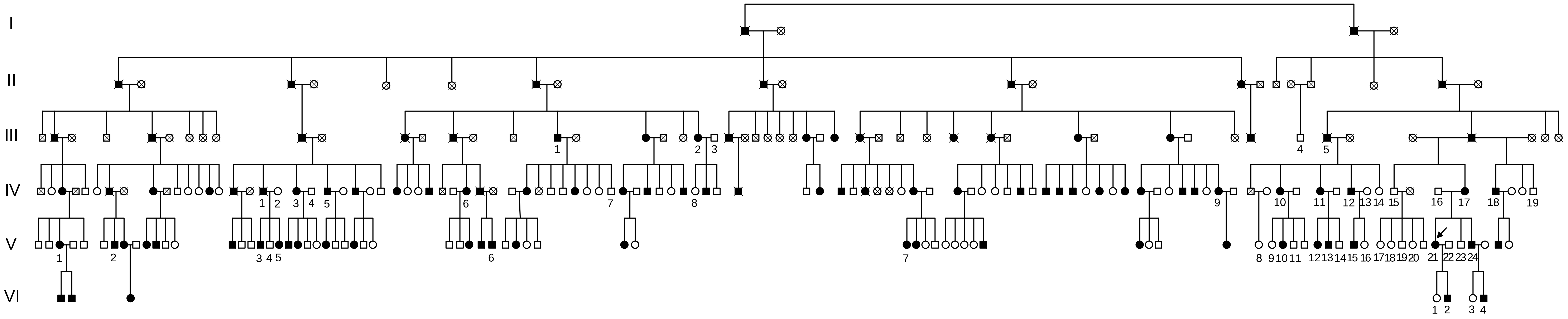

Supplementary Figure 1. **Full pedigree map of CTS Family 1**. The individuals with numbers underneath indicate the family members recruited and analyzed in this study

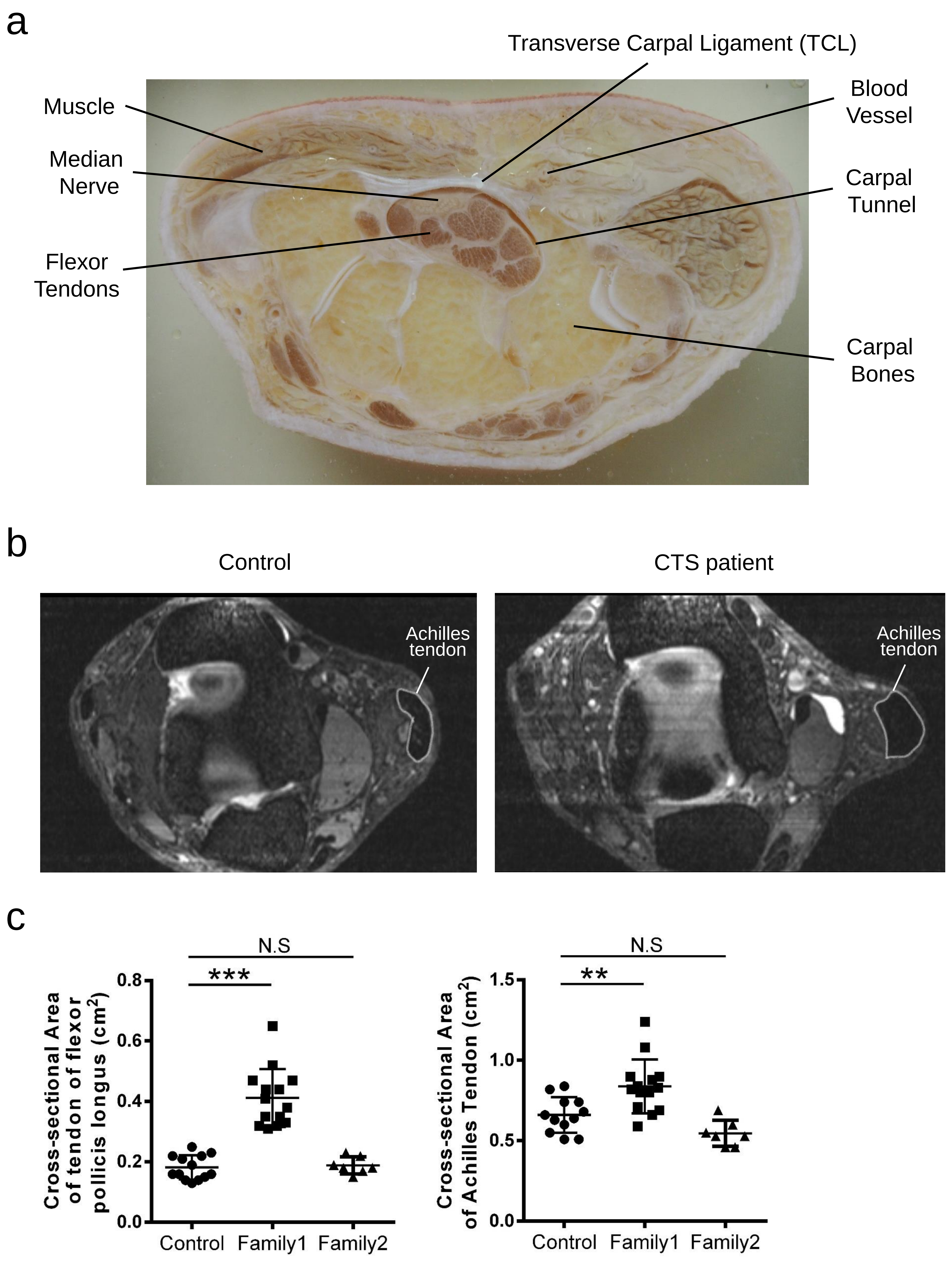

Supplementary Figure 2. Structure of human carpal tunnel and MRI images of familial CTS patients. **a**, cross-sectional structure of the normal human carpal tunnel. **b**, representative MRI images of controls' and CTS patients' Achilles tendons. **c**, MRI results indicate bigger flexor pollicis longus and Achilles tendons in Family 1. Two-tailed *t*-test, **p=0.004, ***p=2.29x10^{-8}. N.S., no Significance, error bars are ± SEM. n=13 in controls, n=14 in Family 1, n=7 in Family 2. Source data are provided as a Source Data file.

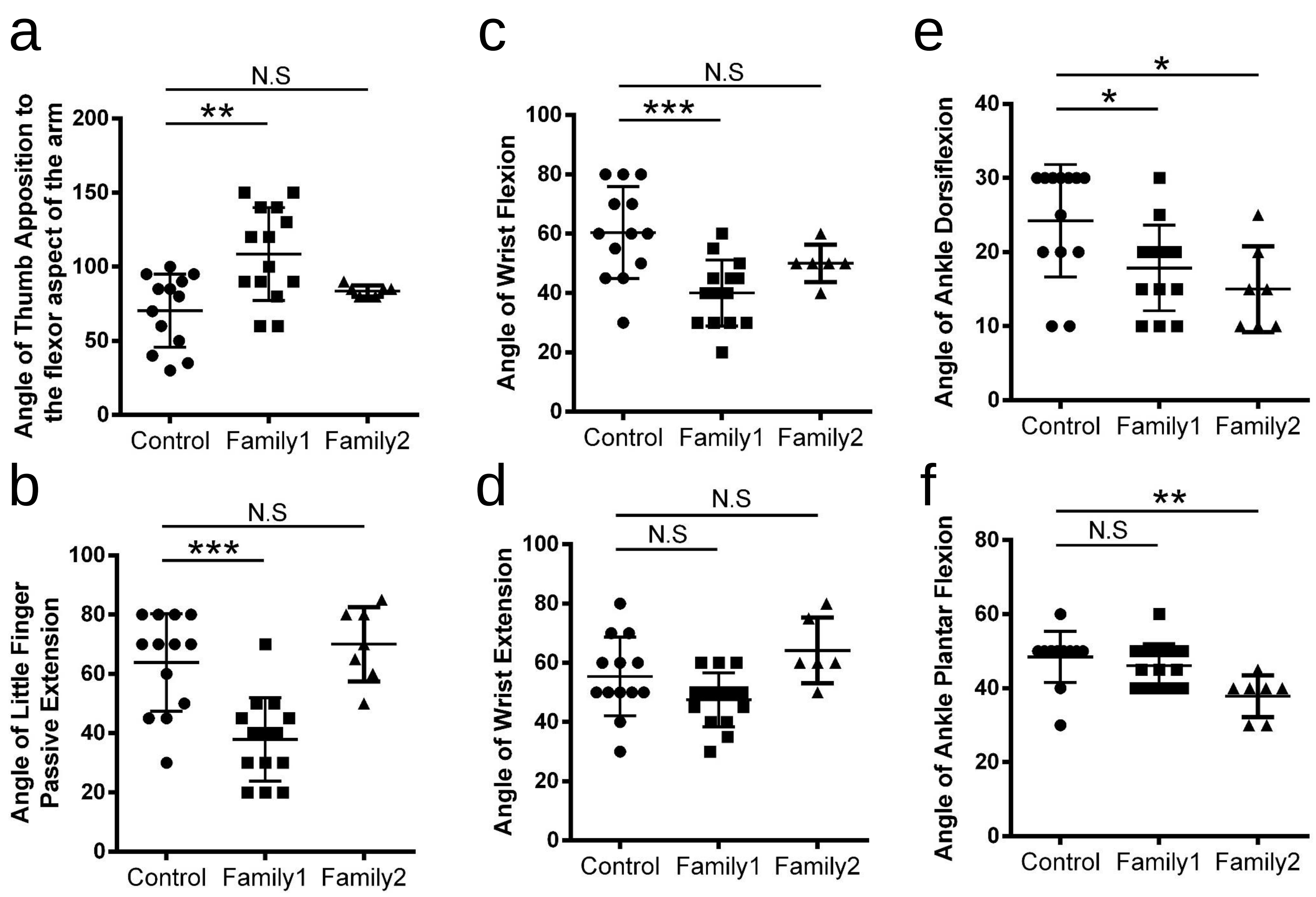

Supplementary Figure 3. Joint flexibility test of CTS patients. Results of joint flexibility test suggest reduced mobility in Family 1 patients' thumbs, little fingers, wrists and ankles, while the mobility of Family 2 patients' ankles are affected. Two-tailed *t*-test, **p=0.002 (a), ***p=0.0001 (b), ***p=0.0005 (c), *p=0.021 (e, Family 1) and 0.012 (e, Family 2), **p=0.002 (f). N.S., no significance, error bars are ± SEM. n=13 in controls, n=14 in Family 1, n=7 in Family 2. Source data are provided as a Source Data file.

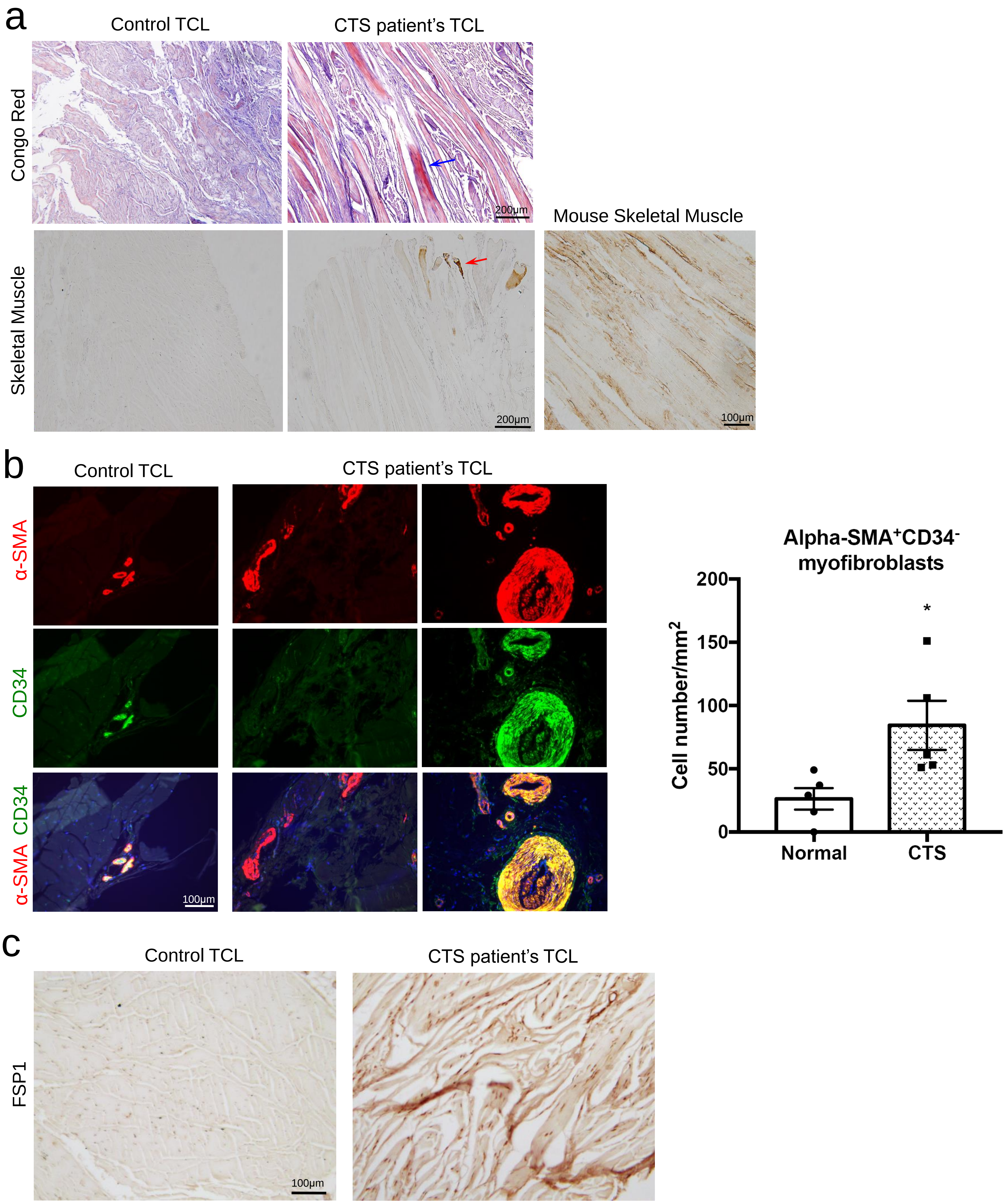

Supplementary Figure 4. Phenotypic analysis of CTS patients' transverse carpal ligaments (TCLs). TCLs were collected from controls' (n=2) and Family 1 patients' (n=3) carpal tunnels. **a**, Congo red staining and immunohistochemical staining of a skeletal muscle specific marker (12/101) show mild amyloidosis (blue arrow) and very few muscle tissues (red arrow) in the analyzed CTS patients' TCLs. A mouse skeletal muscle sample was used as positive control. **b**, immunofluorescent co-staining of α-SMA and CD34 identifies thickened blood vessel walls in CTS patients' TCLs, and statistical counting of α-SMA^{+}CD34^{-} myofibroblasts reveals an increase of fibrosis in CTS patients (five samples each were analyzed, two-tailed *t*-test, *p=0.025, error bars are ± SEM). **c**, Immunohistochemical staining of FSP1 also indicates fibrosis in CTS patients. Source data are provided as a Source Data file.

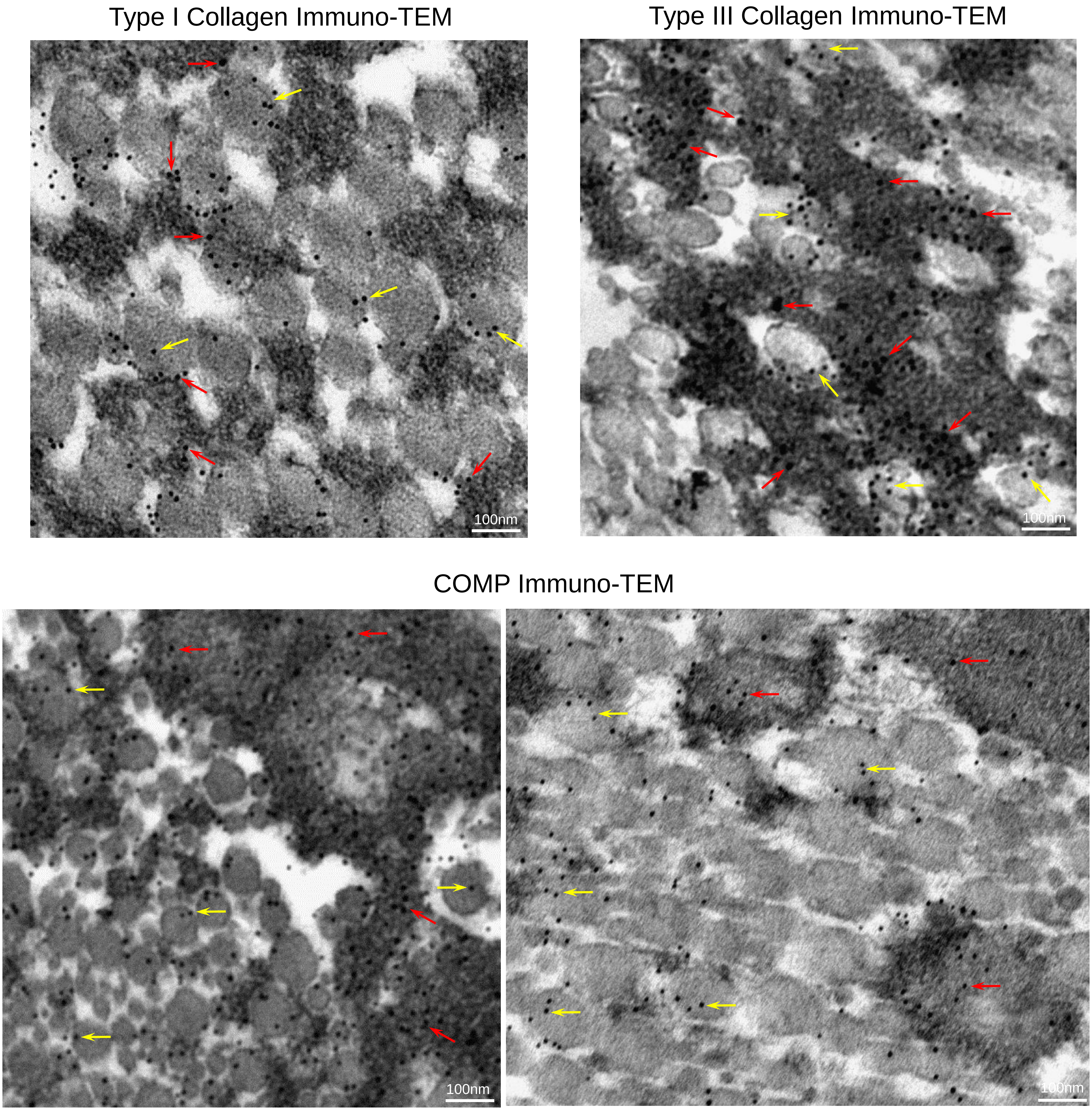

Supplementary Figure 5. Immuno-TEM of CTS patients' transverse carpal ligaments (TCLs). Immuno-TEM detected type I collagen, type III collage, and COMP in both normal (yellow arrows) and ectopic (red arrows) extracellular matrix (ECM) of Family 1 patient's TCL (n=2). The dark small dots are 10nm gold particles labeled on secondary antibodies. Source data are provided as a Source Data file.

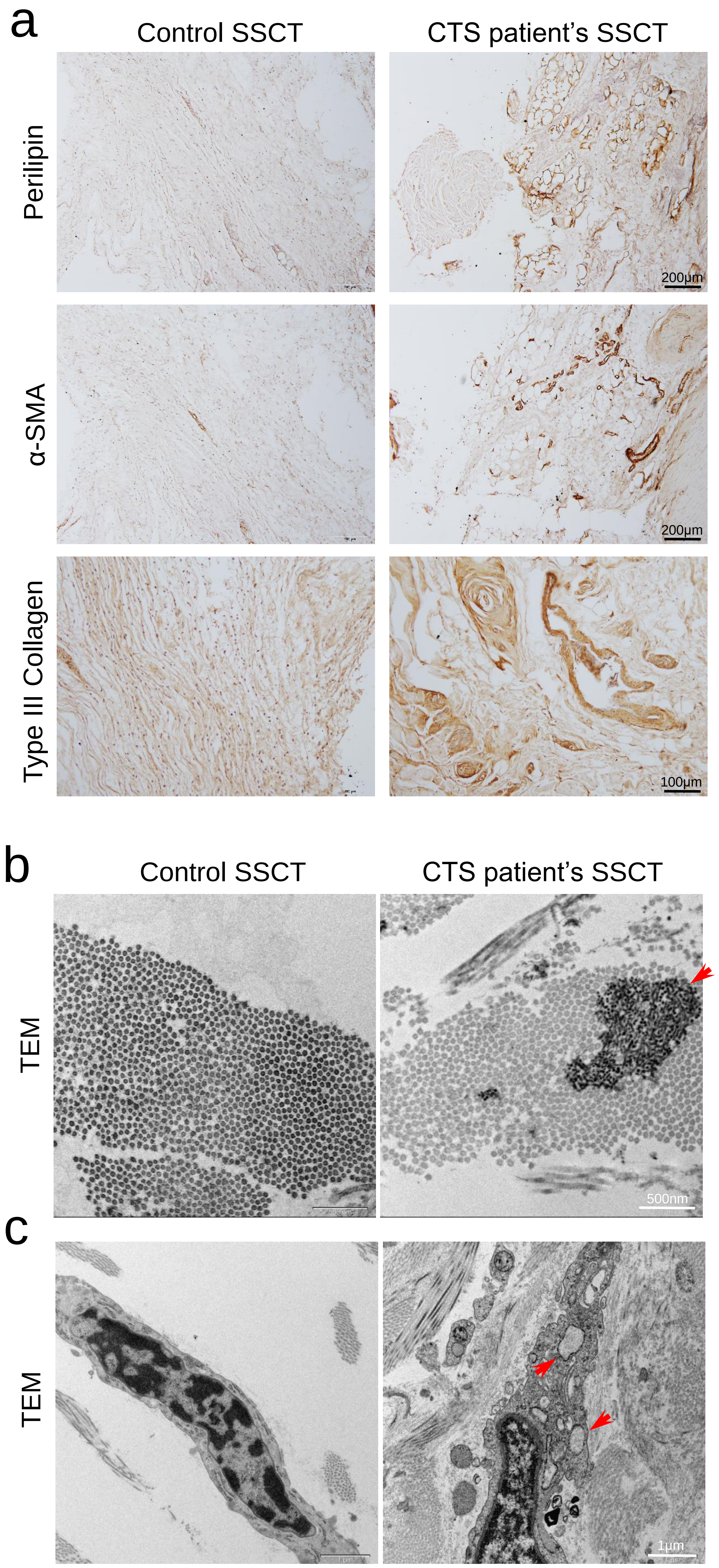

Supplementary Figure 6. Analysis of CTS patients' subsynovial connective tissues (SSCT). SSCT were collected from controls' (n=2) and CTS patients' carpal tunnels (n=2). **a**, Immunohistochemical staining of perilipin, α-SMA and type III collagen shows increases of adipocytes and fibrosis in patient's SSCT. **b**, **c**, transmission electron microscopy (TEM) identifies a number of tiny fibrils (red arrowhead in b) and enlarged ER (red arrowheads in c) in patient's SSCT. Source data are provided as a Source Data file.

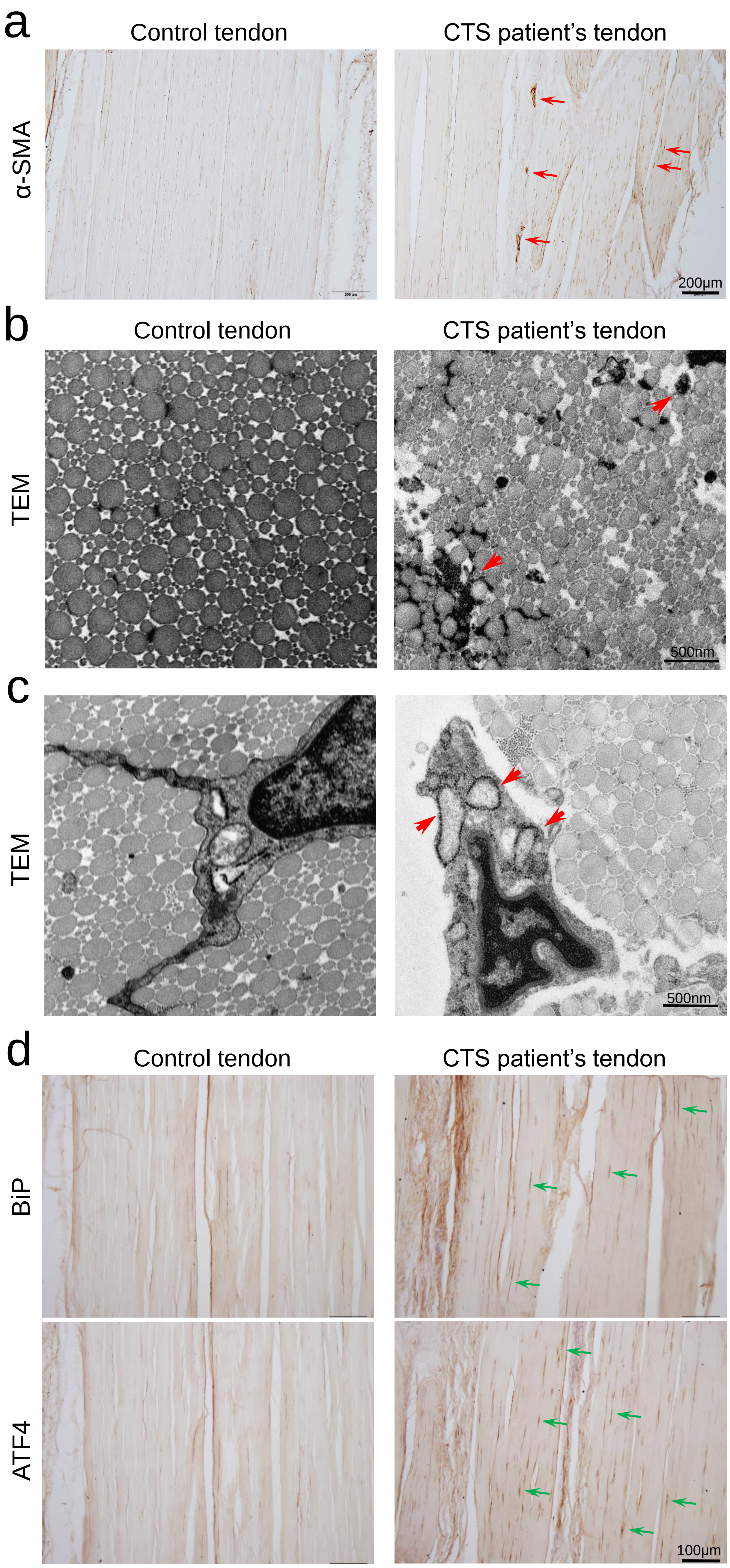

Supplementary Figure 7. Analysis of CTS patients' digital flexor tendons. Digital flexor tendons were collected from controls (n=2) and CTS patients (n=2). **a**, α-SMA staining shows increased fibrosis in patient's flexor tendons (red arrows). **b**, **c**, transmission electron microscopy (TEM) identifies a number of tiny fibrils (red arrowheads in b) and enlarged ER (red arrowheads in c) in patient's digital flexor tendons. **d**, Immunohistochemical staining indicates upregulation of BIP and ATF4 in patient's tendons. Source data are provided as a Source Data file.

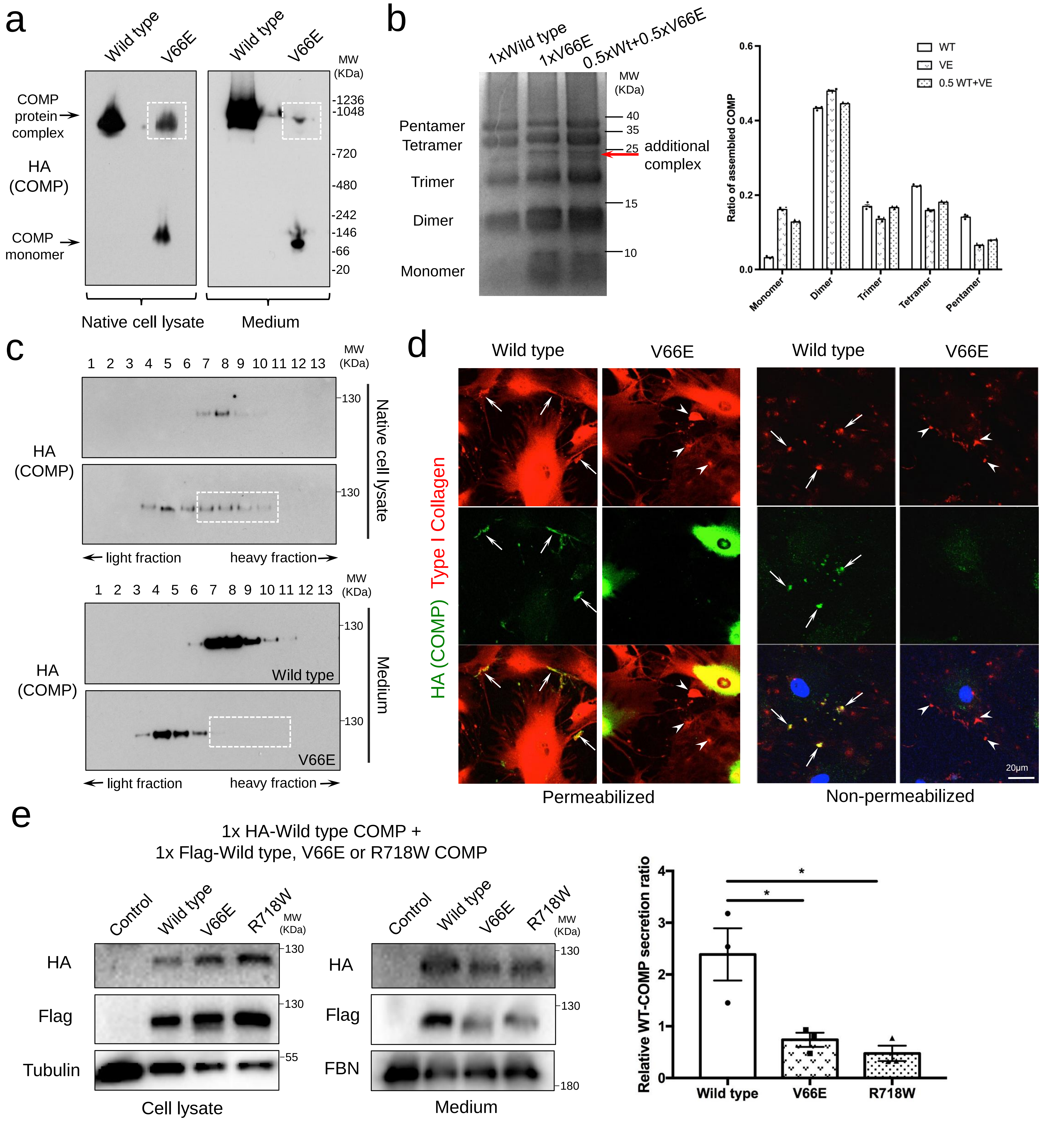

Supplementary Figure 8. Functional characterization of the COMP mutation. Wild type and V66E mutant COMP were expressed in primary tendon cells. Cell lysates and culture medium were respectively collected. **a**, native gel electrophoresis of primary tendon cell lysates or medium reveals that a large amount of V66E mutant COMP exist in monomers. **b**, coiled-coil domain of wild type and V66E-COMP were purified and cross-linked to form oligomer either separately or in a mixed condition. Compared to the wild type group, the groups containing mutant COMP show a large amount of COMP monomer and an additional complex (red arrow). The ratios of different forms of assembled COMP in each group from three experiments are quantified in the right panel, error bars are ± SEM. **c**, the sucrose density gradient ultracentrifugation experiment indicates that protein complex containing wild type or V66E mutant COMP have different molecular weight in primary tendon cells. The V66E mutant COMP preferentially exist in the light fractions. The boxed regions in (a) and (c) suggest impaired secretion of large protein complex containing mutant COMP. **d**, immunofluorescent staining of COMP (green) and type I collagen (red) in primary tendon cells either permeabilized or non-permeabilized. Unlike the wild type COMP, the V66E mutant COMP is not included in the extracellular deposits containing type I collagen. **e**, dominant negative effect of the mutant COMP on wild type COMP in primary tenocytes. The mutant COMP (Flag-tagged) impairs the secretion of the wild type COMP (HA-tagged). FBN, fibronectin. The secretion ratios of HA-COMP with different forms of Flag-COMP from three experiments are quantified and summarized in the right panel, two-tailed *t*-test, *p=0.034 (V66E) and 0.022 (R718W), error bars are ± SEM. Source data are provided as a Source Data file.

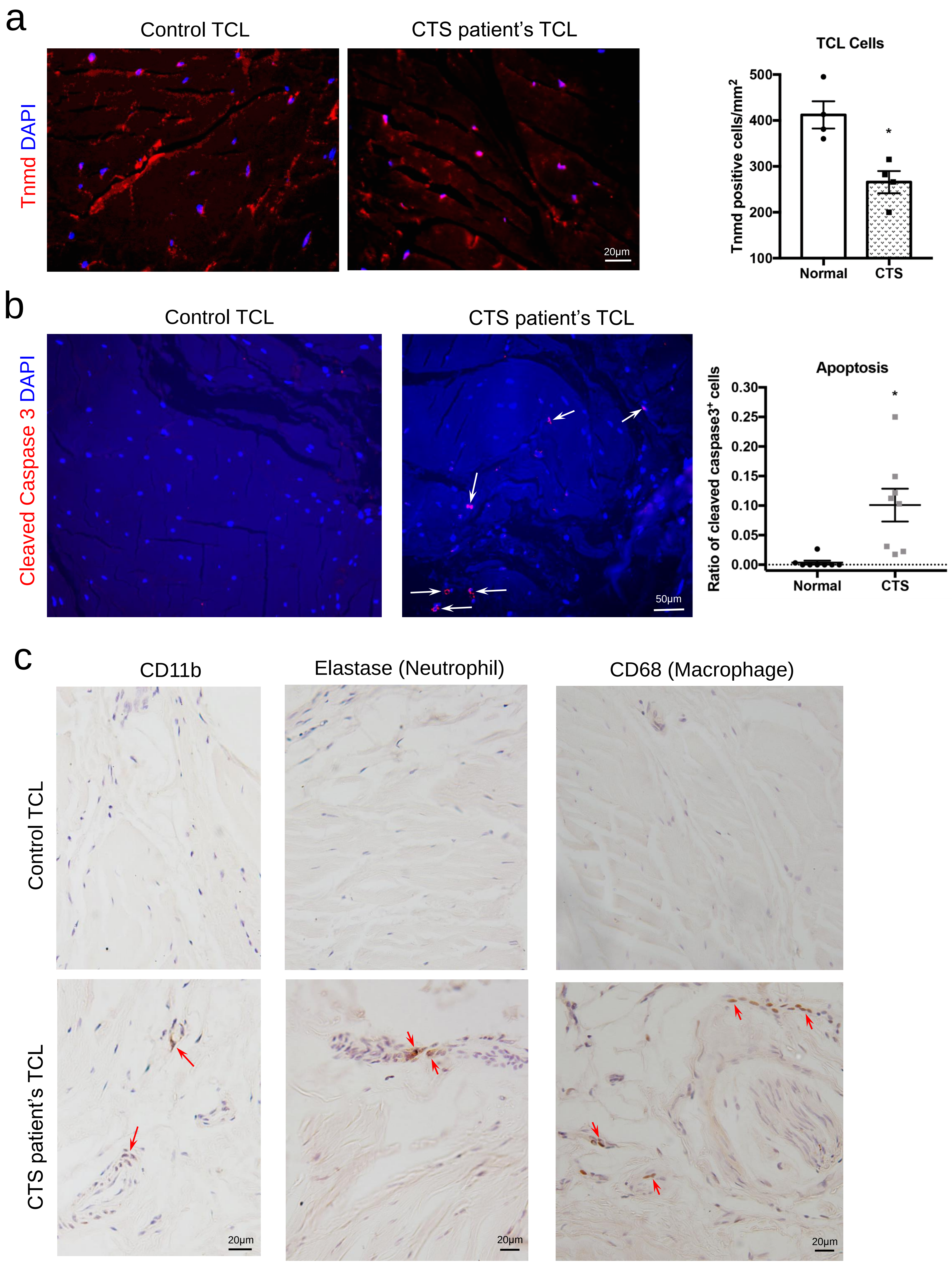

Supplementary Figure 9. Cell death and inflammation in CTS patients' TCLs. TCLs were collected from controls' (n=2) and Family 1 patients' (n=3) carpal tunnels. **a**, reduced number of tenomodulin-labeled ligament cells in patients' TCLs. Two-tailed *t*-test, *p=0.009, error bars are ± SEM. Four samples each were analyzed. **b**, cleaved caspase 3 staining indicates an increase of apoptosis in patients' TCLs. Two-tailed *t*-test, *p=0.004, error bars are ± SEM. Eight samples each were analyzed. **c**, immunostaining of CD11b, elastase (neutrophil) and CD68 (macrophage) identifies signs of inflammation in patients' TCLs. Source data are provided as a Source Data file.

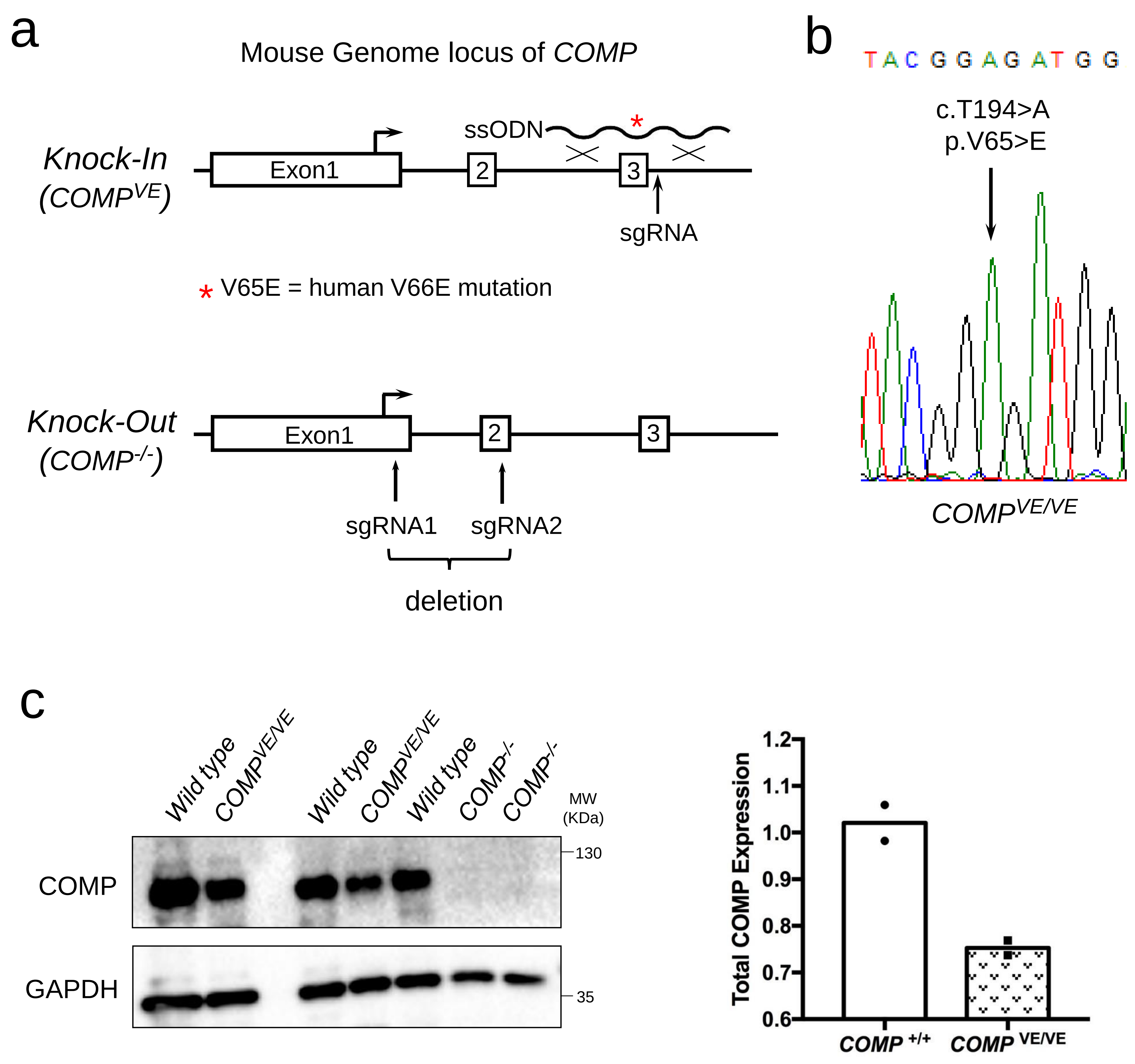

Supplementary Figure 10. Generation of *COMP* mutant mice. a, two *COMP* mutant mouse models were generated by CRISPR/Cas9-mediated knock-in and knock-out in mouse zygotes. The mouse V65E mutation (equivalent to human V66E mutation) was introduced into the endogenous *COMP* locus by ssODN-mediated recombination. For *COMP* null, two sgRNA were used to target exon1 and exon2 to generate a deletion. ssODN, single-stranded oligodeoxynucleotides; sgRNA, single-guide RNA. **b**, the p.V65E homozygous mutation in cDNA of the *COMP*$^{VE/VE}$ mouse. **c**, the western blot analysis of mouse tails confirms the loss of COMP protein in *COMP* null mice. The level of V65E mutant COMP proteins is reduced. The COMP protein level is quantified from two experiments and summarized in the right panel. Source data are provided as a Source Data file.

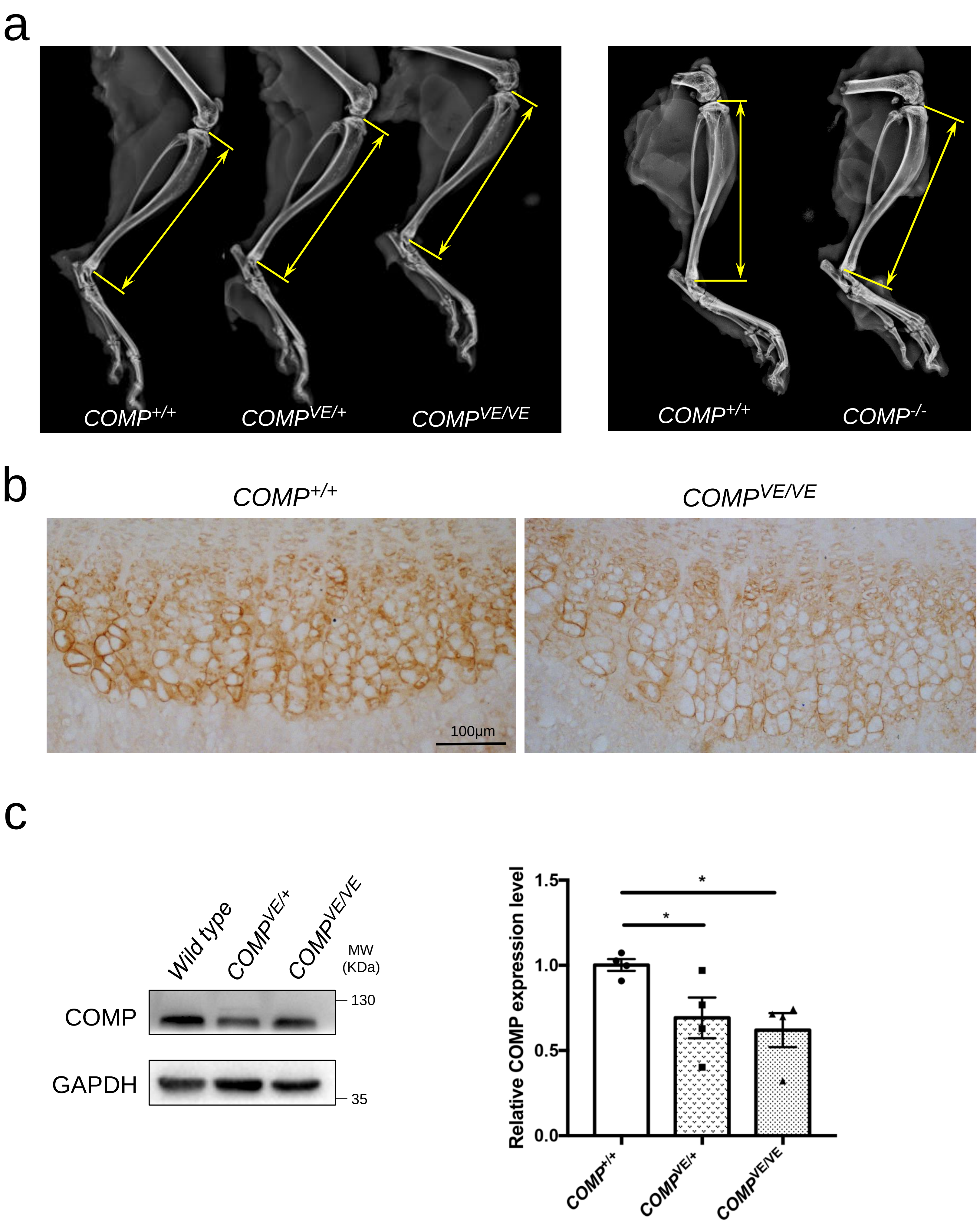

Supplementary Figure 11. Analysis of long bones of *COMP* mutant mice. a, X-ray radiography of 20-week old wild type, *COMP*^{VE} and *COMP* null mutant mouse hindlimbs shows no obvious long bone phenotype. The lengths of tibia are comparable in different genotypes. Each genotype has at least three mice analyzed. **b**, COMP immunostaining in 10-day old wild type and *COMP*$^{VE/VE}$ mouse growth plates. The secretion of mutant COMP appears normal, but total protein level is reduced, which is consistent with the immunoblotting results of mouse chondrocytes (**c**). The COMP protein level is quantified from four experiments and summarized in the right panel, two-tailed *t*-test, *p=0.047 and 0.011, error bars are ± SEM. Source data are provided as a Source Data file.

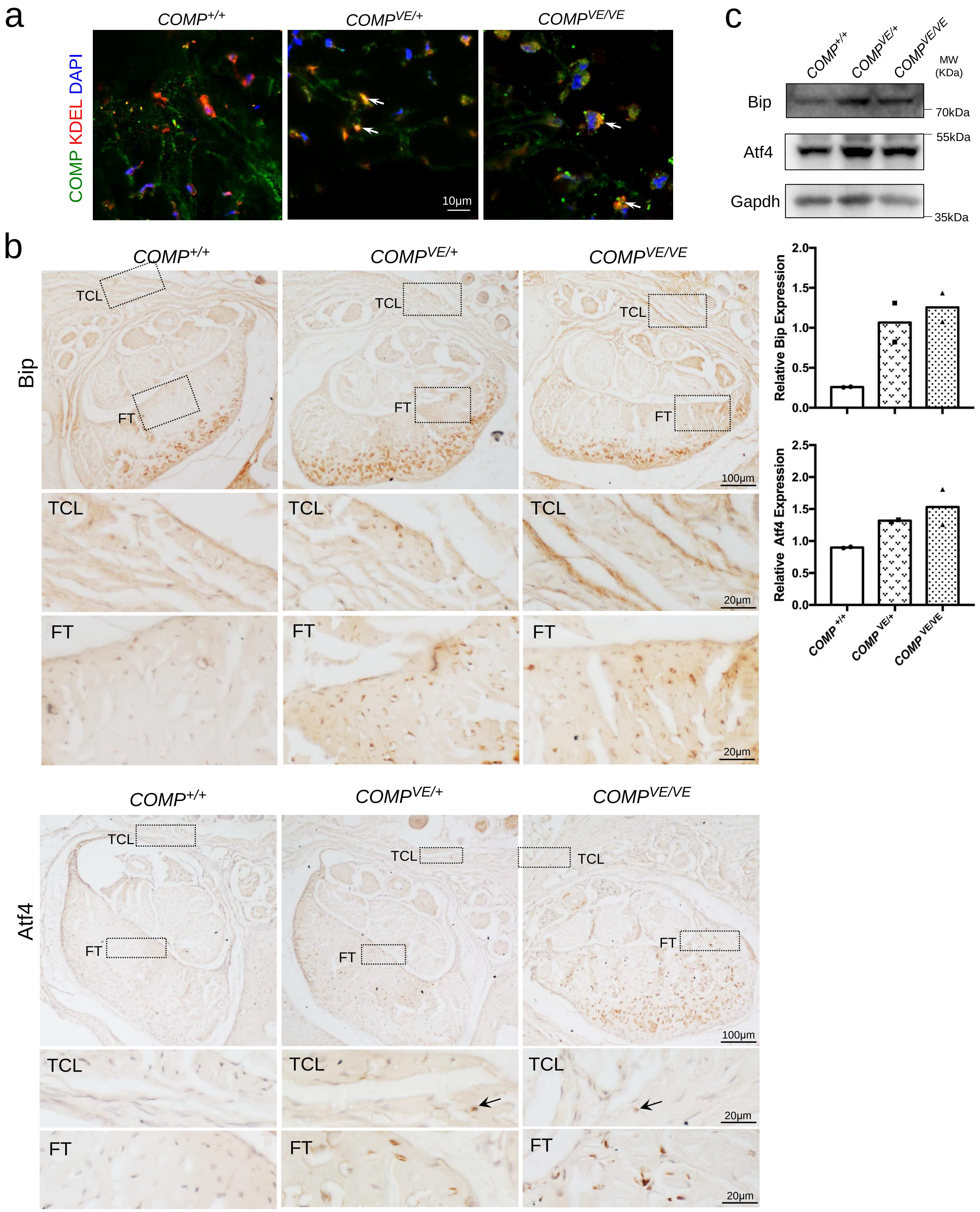

Supplementary Figure 12. ER stress and UPR activation in *COMP*^{VE} mutant. **a**, co-localization of COMP (green) and ER (recognized by KDEL antibody, red) in 20-week old mutant mouse flexor tendon cells. DAPI stains cell nucleus (blue). **b**, immunohistochemical staining of 20-week old mouse carpal tunnels indicates upregulation of Bip and Atf4 expression in *COMP* mutants. Signals in transverse carpal ligaments (TCL) and flexor tendons (FT) are magnified and shown in the below panels. **c**, immunoblotting of *COMP* mutant tendons shows upregulation of Bip and Atf4. The western blot results from two experiments are quantified and summarized in the below panels. Source data are provided as a Source Data file.

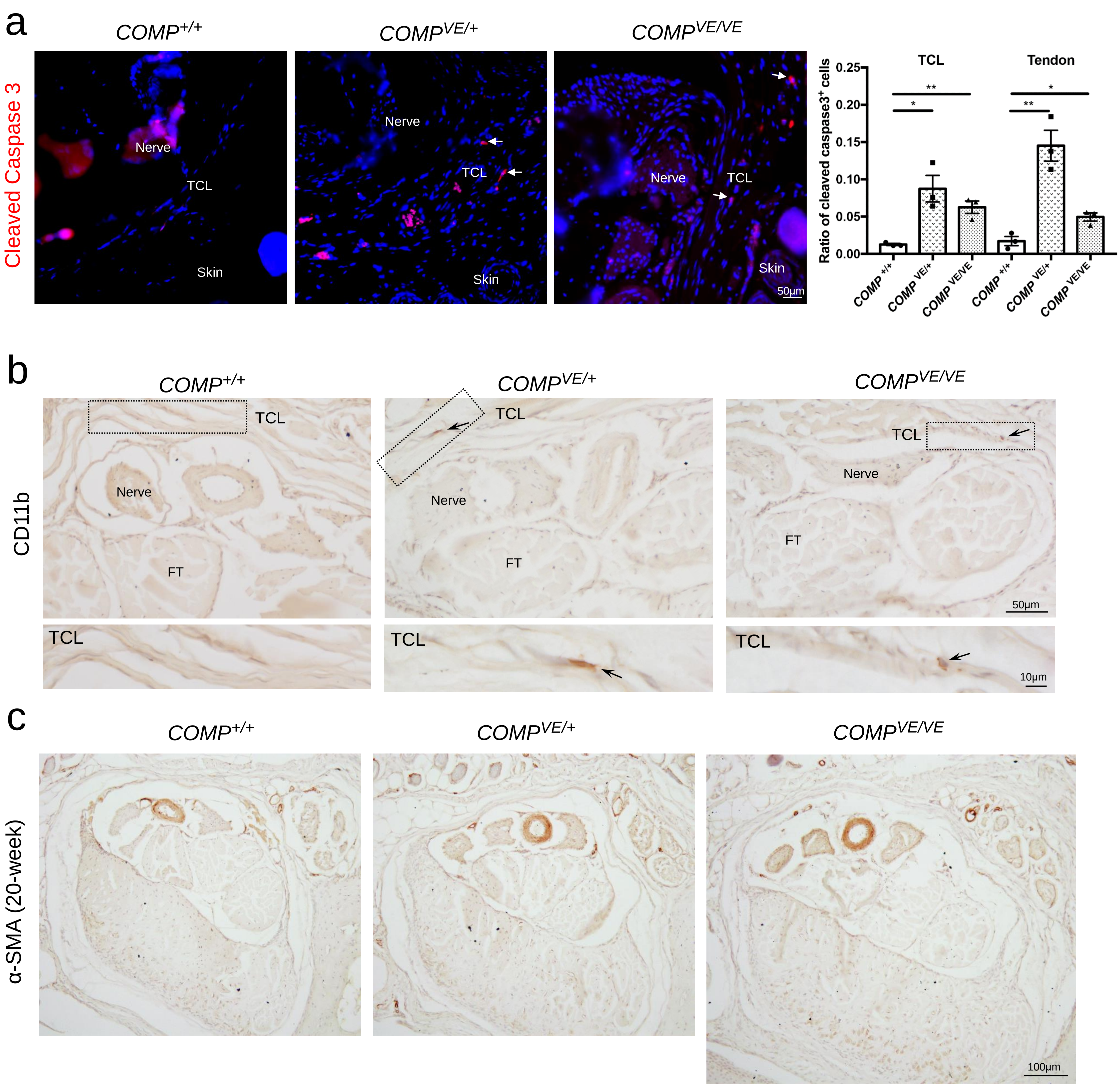

Supplementary Figure 13. Cell death and inflammation in *COMP*^{VE} mutant. **a**, cleaved caspase 3 staining indicates increase of apoptosis in 20-week old *COMP* mutants. Two-tailed *t*-test, TCL, *p=0.014, **p=0.004; Tendon, *p=0.017, **p=0.004, error bars are ± SEM. Three samples for each were analyzed. **b**, immunostaining of CD11b identifies slight inflammation in 20-week old mutant carpal tunnels. Signals in transverse carpal ligament (TCL) are magnified and shown in the below panels. **c**, α-SMA staining in 20-week old mouse carpal tunnels. n=3 per genotype for each experiment. Source data are provided as a Source Data file.

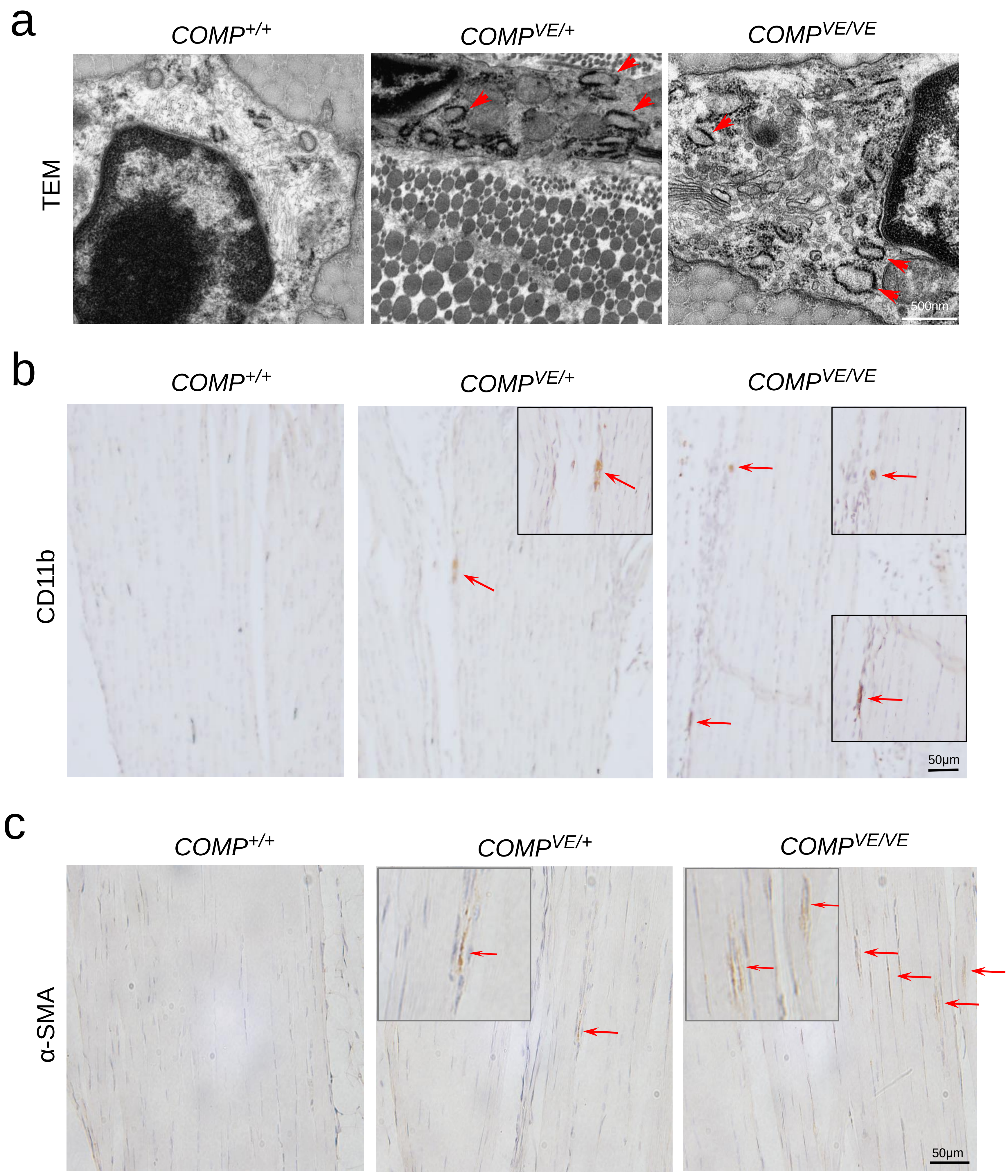

Supplementary Figure 14. Analysis of *COMP^{VE}* Achilles tendons. **a**, transmission electron microscopy (TEM) shows distended ER in 20-week old *COMP^{VE}* mutant mice (red arrows). **b**, sign of inflammation (CD11b) in 20-week old *COMP^{VE}* mutant Achilles tendons. **c**, immunohistochemical staining of α-SMA indicates slight fibrosis in 20-week old *COMP^{VE}* mutant Achilles tendons. Signals (red arrows) are magnified and shown in the insets. n=3 per genotype for each experiment. Source data are provided as a Source Data file.

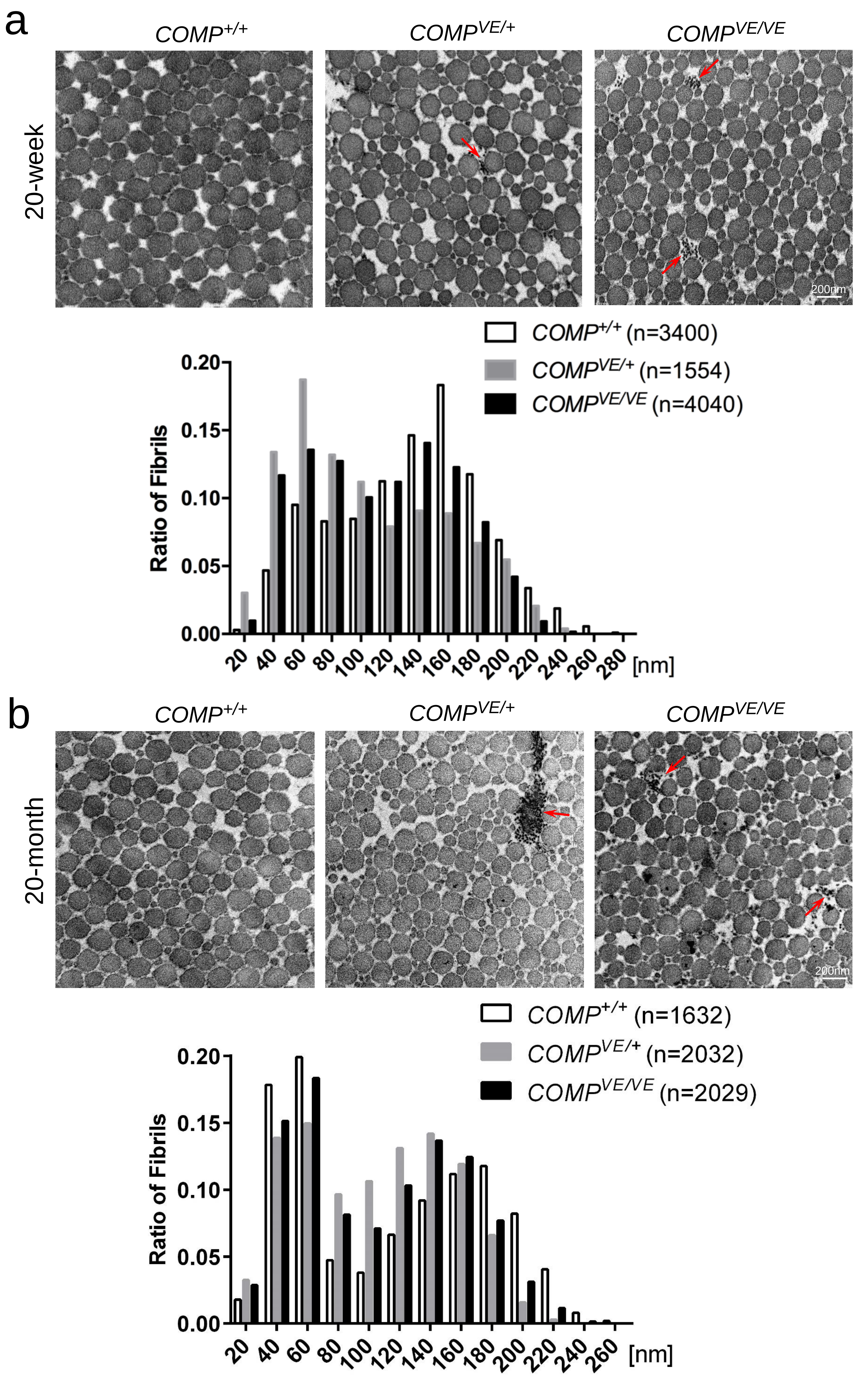

Supplementary Figure 15. ECM ultrastructure of *COMP^{VE}* mice. a, **b**, transmission electron microscopy (TEM) reveals the organization of ECM in 20-week (a) or 20-month (b) old wild type and *COMP^{VE}* Achilles tendons (n=3 per genotype). The diameters of collagen fibrils (except those ectopic tiny fibrils in the mutant, red arrows) in three animals of each group were measured (n=1500~4000 for 20-week, n=1600~2000 for 20-month). The distribution difference of collagen fibril diameters was calculated by two-tailed Mann–Whitney *U* test, p=1.09x10^{-115} (*COMP$^{VE/+}$* vs *COMP$^{+/+}$*) and 1.26x10^{-98} (*COMP$^{VE/VE}$* vs *COMP$^{+/+}$*) for 20-week old mice; p=5.55x10^{-9} (*COMP$^{VE/+}$* vs *COMP$^{+/+}$*) and 2.39x10^{-6} (*COMP$^{VE/VE}$* vs *COMP$^{+/+}$*) for 20-month old mice. Ectopic tiny fibrils are more prominent in older mice. Source data are provided as a Source Data file.

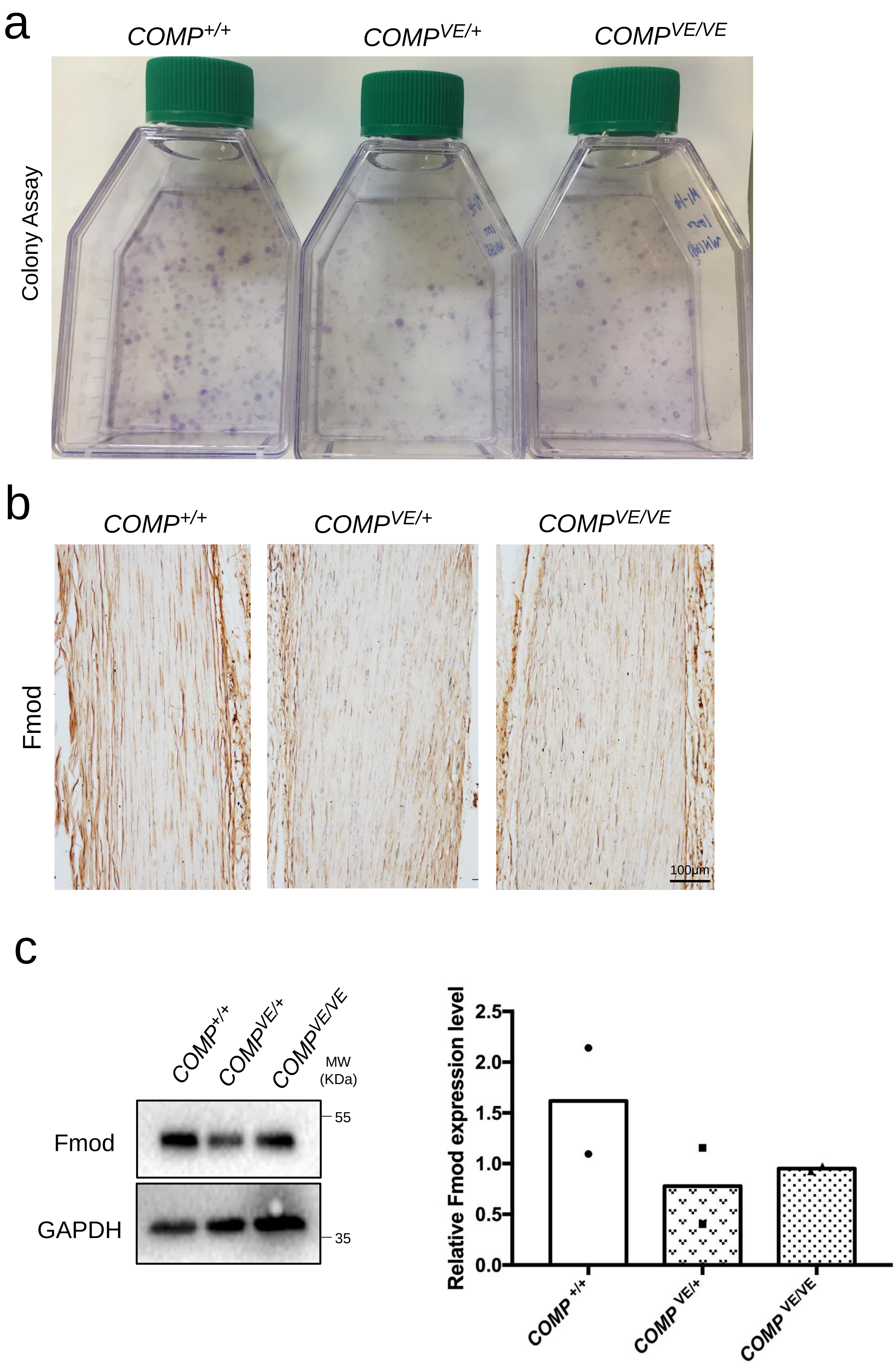

Supplementary Figure 16. Tendon stem/progenitor cells (TSPCs) potential in *COMP*^{VE} mice. a, primary tendon cells were isolated from 4-week old Achilles tendons and cultured *in vitro*. Colony assay of isolated tendon cells shows fewer colonies formed by *COMP* mutant mice, suggesting a lower TSPC potential caused by the *COMP* mutation. See Figure 7b for statistical results. **b**, **c**, immunohistochemistry (b) and western blot (c) show down-regulation of fibromodulin (Fmod), a critical niche factor maintaining TSPCs, in 20-week old *COMP*^{VE} mutant Achilles tendons. The protein levels of Fmod from two experiments are quantified in the right panel. Source data are provided as a Source Data file.

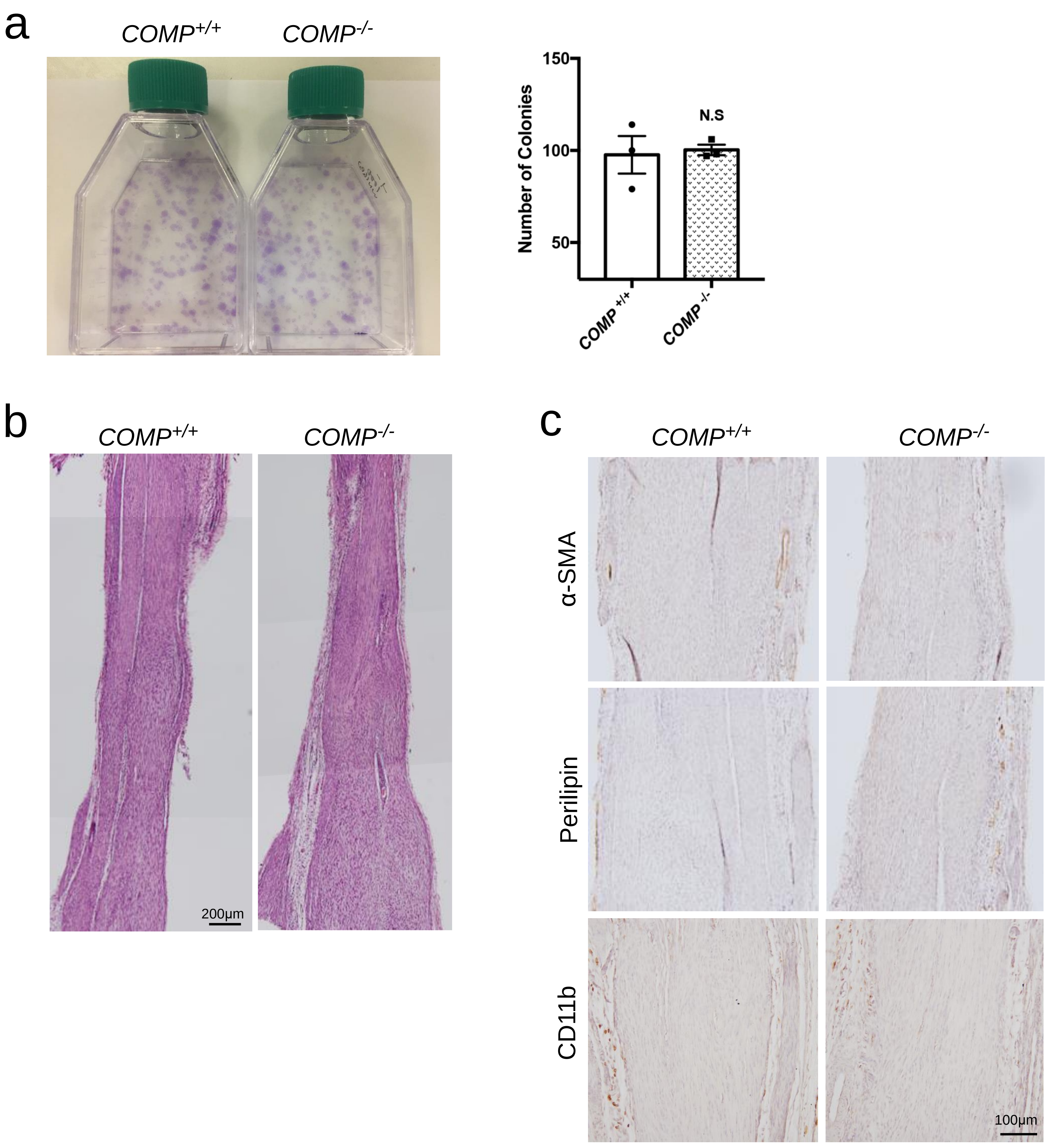

Supplementary Figure 17. Analysis of COMP *null* mice. **a**, Colony assay of primary tendon cells isolated from 4-week old Achilles tendons. Similar number of TSPC colonies formed by wild type and *COMP*$^{-/-}$ mice (two-tailed *t*-test, p=0.813. N.S., no significance, error bars are ± SEM). The experiment was repeated three times. **b**, healing of injured Achilles tendons in neonatal *COMP*$^{-/-}$ mice is comparable to that in wild type mice (n=3 per genotype). **c**, no obvious signs of fibrosis (α-SMA), adipogenesis (Perilipin) or inflammation (CD11b) are observed in both wild type and *COMP*$^{-/-}$ Achilles tendons 14 days after injury (n=3). Source data are provided as a Source Data file.

Family	Patient	Disease	Gender	Age	Age of onset	Height (cm)	Nighttime Pain	Numbness	Thenar Atrophy	Tinel's Sign	Phalen's Test	Electrophysiologic test of median nerve		Tenosynovitis
												DML (ms)	NCV (ms)	
1	IV-6	CTS	F	62	40+	147	+	+	+	+	+	NR (L); NR (R)	NR (L); NR (R)	N/A
1	IV-11	CTS	F	45	28	160	+	+	+	+	+	NR (L); NR (R)	NR (L); NR (R)	+
1	V-1	CTS	F	58	30	159	+	+	+	+	+	NR (L); NR (R)	NR (L); NR (R)	N/A
1	V-7	CTS	F	38	35	160	+	+	+	+	+	9.13 (L); 14.1 (R)	NR (L); NR (R)	+
1	V-10	CTS	F	43	30	161	+	+	+	+	+	NR (L); NR (R)	NR (L); NR (R)	+
1	V-12	CTS	F	47	27	161	+	+	+	+	+	NR (L); NR (R)	NR (L); NR (R)	+
1	V-21	CTS	F	50	18	155	+	+	+	+	+	4.31(L)*; 4.58 (R)*	35.2 (L)*; 36.3 (R)*	+
1	IV-18	CTS	M	51	40+	172	+	+ (L)	+ (R)	+	+	7.23 (L); NR (R)	NR (L); NR (R)	+
1	V-2	CTS	M	49	30+	171	+	+	+	+	+	NR (L); NR (R)	NR (L); NR (R)	+
1	V-6	CTS	M	31	26	161	+	+	+	+	+	6.15 (L)*; 8.17 (R)*	29.9 (L)*; NR (R)*	+
1	V-13	CTS	M	45	23	171	+	+	+	+	+	NR (L); NR (R)	NR (L); NR (R)	N/A
1	V-15	CTS	M	42	33	170	+	+	+	+	+	9.44 (L); 13.2 (R)*	NR (L); NR (R)*	—
1	V-24	CTS	M	45	21	162	+	+	+	+	+	NR (L); NR (R)	NR (L); NR (R)	+
1	VI-4	CTS	M	25	16	166	—	+	—	+	+	3.48 (L); 3.5 (R)	63.7 (L); 60 (R)	N/A
2	III-3	CTS & MED	F	51	36	156	+	+	+	—	—	3.8 (L); 3.39 (R)	44 (L); 40 (R)	—
2	III-5	CTS & MED	F	51	40	160	—	—	+	+	—	8.39 (L); 4.79 (R)	NR (L); 35 (R)	+
2	III-7	CTS & MED	F	48	37	160	+	+	+	+	+	6.04 (L); NR (R)	33.7 (L); NR (R)	+
2	III-8	CTS & MED	F	47	38	156	+	+	+	+	+	5.1 (L); 4.22 (R)	32 (L); 39 (R)	—
2	II-3	CTS & MED	M	71	50+	150	+	+	+	+	+	4.48 (L); 3.91 (R)	41 (L); 35 (R)	—
2	III-2	CTS & MED	M	55	50	165	—	+	—	+	—	6.41 (L); 5.36 (R)	27 (L); 28 (R)	+
2	III-10	CTS & MED	M	41	30+	158	—	+	—	+	—	4.01 (L); 4.38 (R)	47 (L); 47 (R)	—
1	IV-10	Unaffected	F	54	—	160	+	—	—	—	—	4.53 (L); 5.16 (R)	46 (L); 43 (R)	—
1	V-8	Unaffected	F	45	—	164	—	—	—	—	—	4.33 (L); 3.83 (R)	48.3 (L); 48.7 (R)	—
1	V-20	Unaffected	F	43	—	151	—	—	—	—	—	3.33 (L); 4.0 (R)	45.8 (L); 42.9 (R)	—
1	IV-15	Unaffected	M	69	—	155	—	—	—	—	—	4.04 (L); 3.15 (R)	55.1 (L); 59.4 (R)	—
1	V-19	Unaffected	M	46	—	162	—	—	—	—	—	4.22 (L); 4.04 (R)	56.6 (L); 49.4 (R)	—
1	V-23	Unaffected	M	47	—	163	—	—	—	—	—	3.17 (L); 3.19 (R)	61.9 (L); 58.4 (R)	—
2	II-4	Unaffected	M	66	—	166	—	—	—	—	—	4.43 (L); 4.84 (R)	44 (L); 43 (R)	—
2	III-1	Unaffected	F	58	—	156	—	+	—	—	—	5.0 (L); 5.31 (R)	40 (L); 40 (R)	—
2	III-4	Unaffected	M	57	—	172	—	—	—	+	—	4.58 (L); 4.17 (R)	N/A	—
Non-family	Control 1	Unaffected	F	46	—	162	—	—	—	—	—	3.72 (R)	63.2 (R)	—
Non-family	Control 2	Unaffected	M	33	—	162	—	—	—	—	—	3.33 (R)	58.3 (R)	—
Non-family	Control 3	Unaffected	M	28	—	164	—	—	—	—	—	3.01 (R)	58.1 (R)	—
Non-family	Control 4	Unaffected	M	30	—	173	—	—	—	—	—	3.54 (R)	58.0(R)	—
3	1	PSACH	F	58	56	145	—	+	+	+	—	3.39 (L) 3.79 (R)	39.7 (L) 41.3 (R)	+
3	2	PSACH	M	51	49	150	—	+	—	+	—	5.29 (L) 4.33 (R)	35.3 (L) 32.2 (R)	—
3	3	PSACH	F	30	—	150	—	—	—	+	—	3.04 (L) 2.88 (R)	57.8 (L) 53.2 (R)	—

Supplementary Table 1. Patient information. NR: No Response; L: Left; R: Right. * Data collected after surgery

Position (hg19)	Marker	Linkage	Haplotype
17,308,106	D19s593	No	-
17,576,566	D19s579	No	-
17,917,874	D19s915	No	-
18,343,060	D19s212	Yes	243
18,473,781	D19s898	Yes	-
18,753,110	D19s895	Yes	162
19,161,981	D19s566	Yes	187
19,383,373	19)19383373	Yes	259
19,735,426	19)19735426	Yes	300
20,065,054	D19s407	Yes	147
20,360,262	D19s546	Yes	346
20,916,455	D19s911	Yes	252
21,194,295	19)21194295	Yes	312
21,361,456	D19s925	Yes	293
21,682,673	D19s215	Yes	283
22,049,921	D19s401	Yes	379
22,495,123	D19s568	Yes	282
23,622,669	D19s910	Yes	273

Supplementary Table 2. Fine mapping with microsatellite markers. Further genotyping with microsatellite markers confirmed the linkage of the chromosome 19p12-13.11. A disease-associated microsatellite haplotype is identified.

Uncropped blots of Figure 4a and Figure 5a

Figure.4a

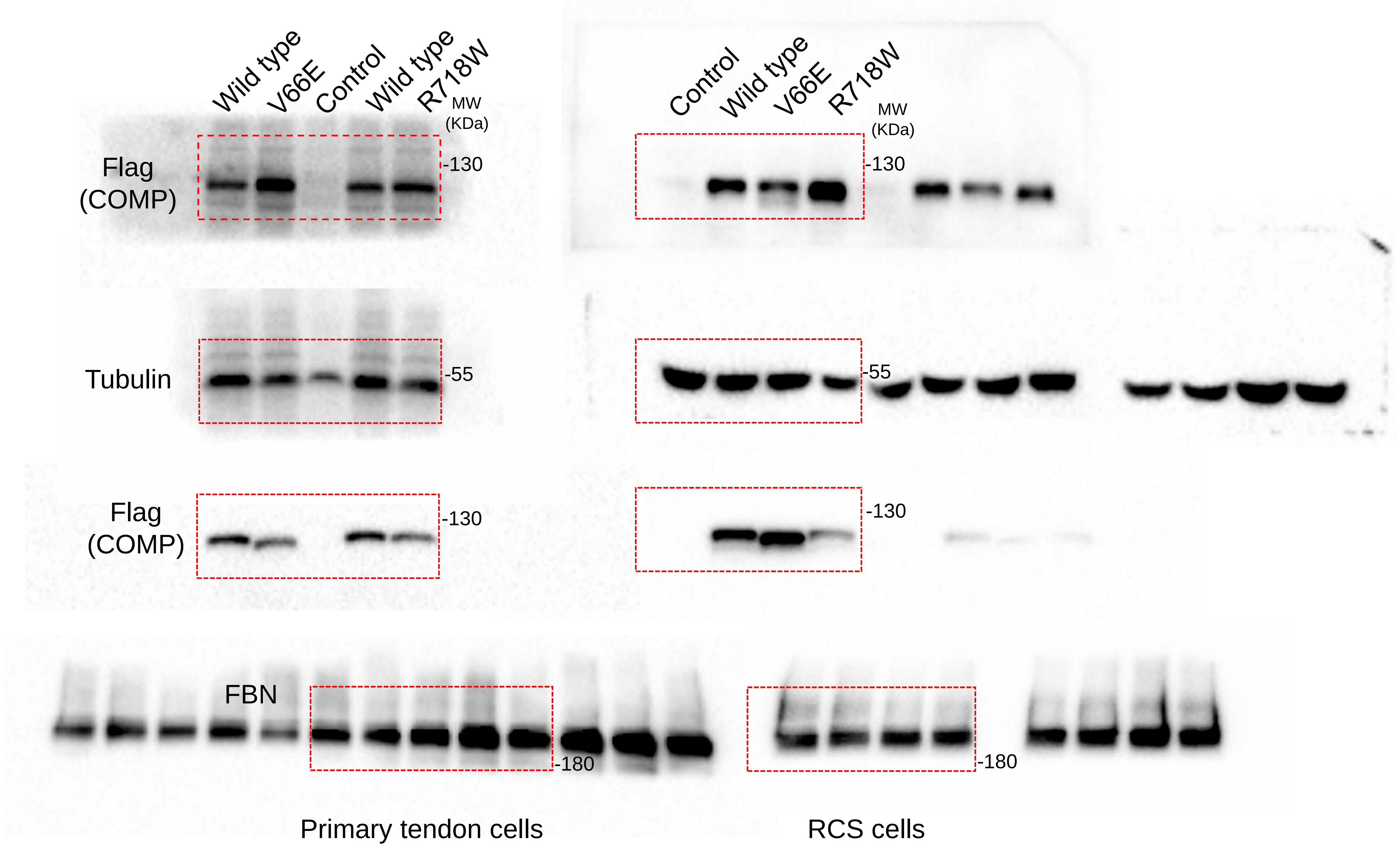

Figure. 5a

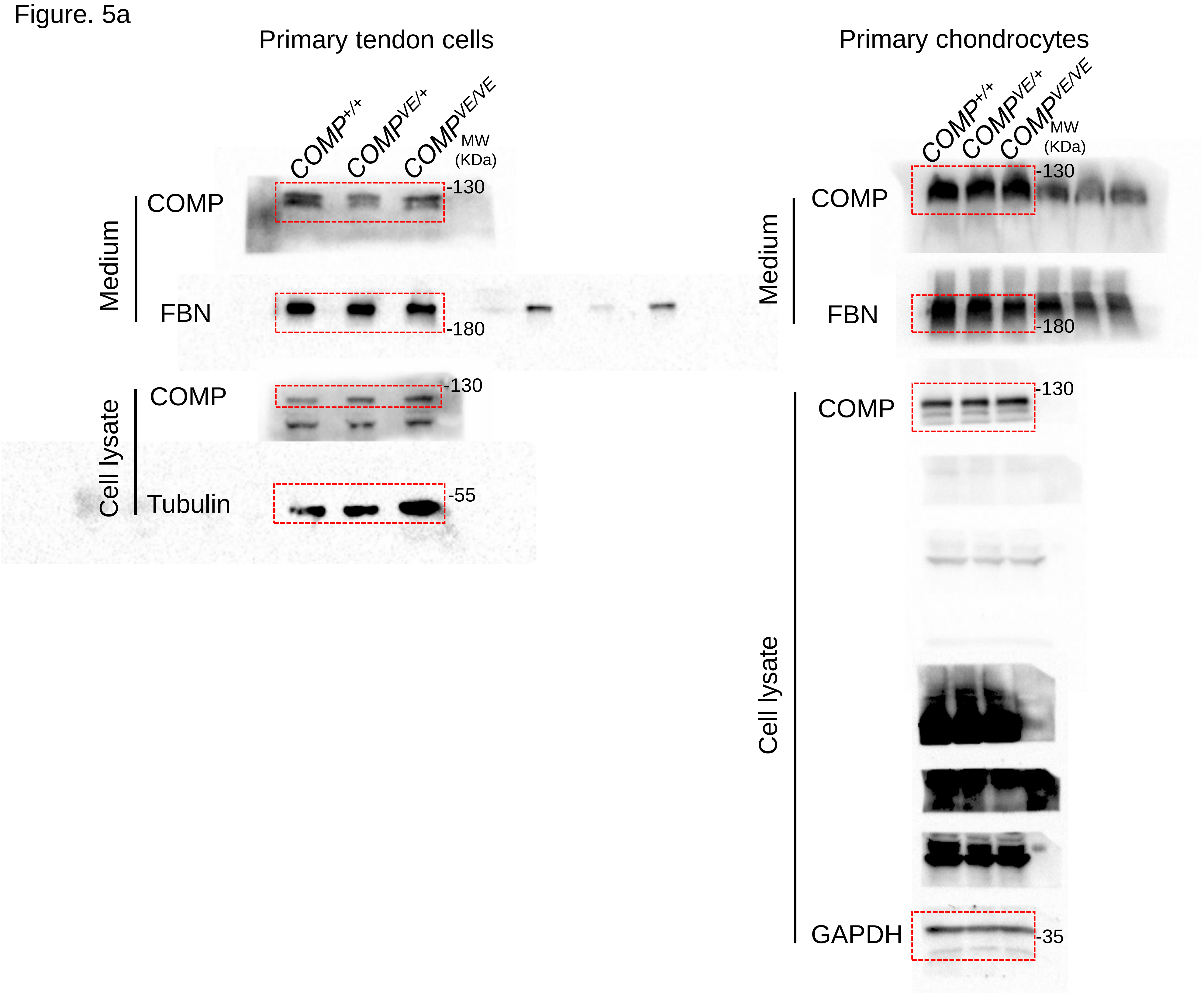

Uncropped blots of Figure 5b-d

Figure.5b

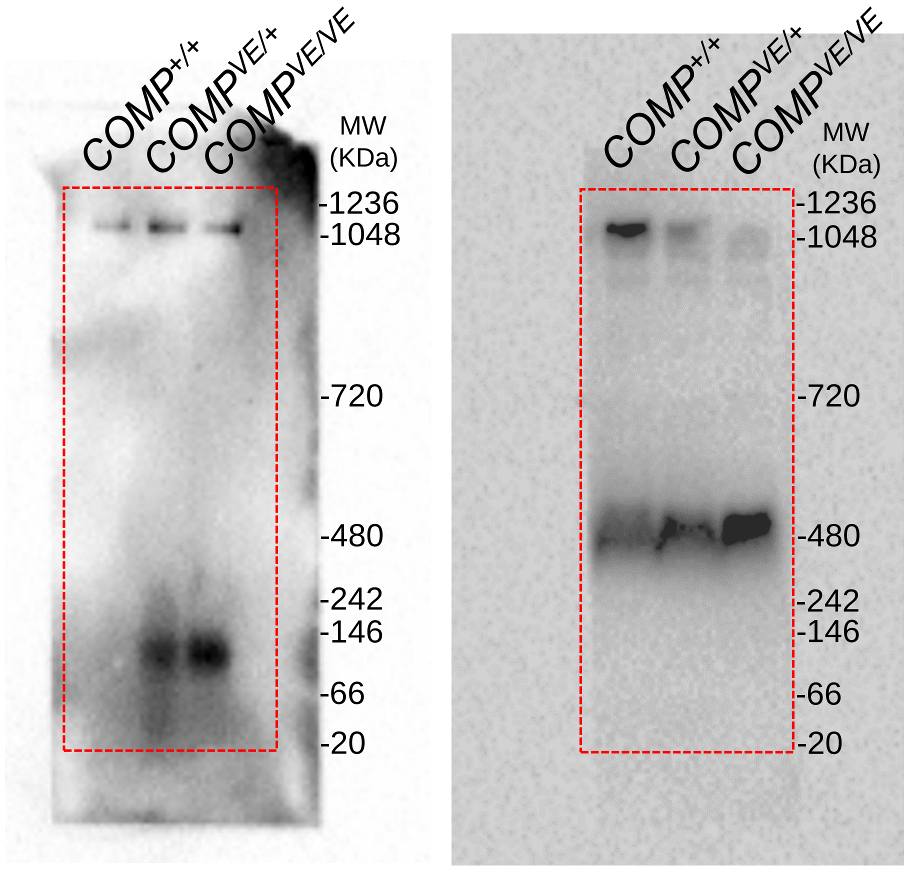

Primary tendon cells

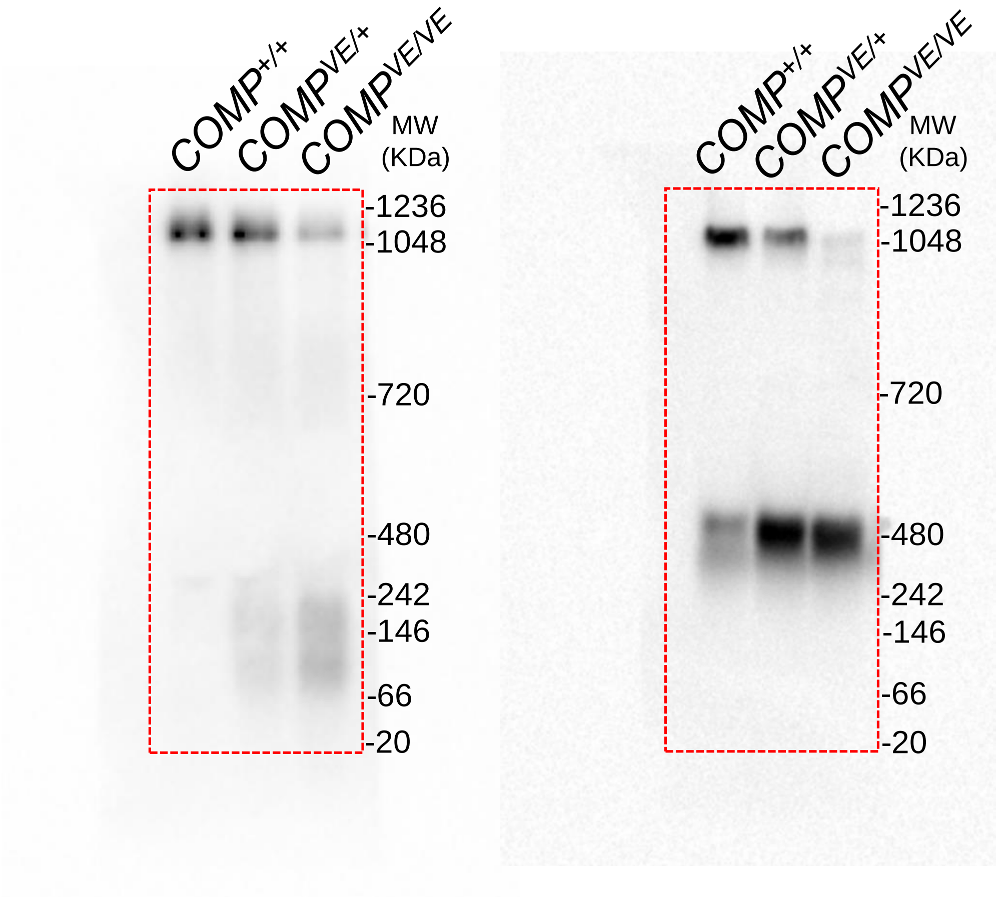

Primary chondrocytes

Figure.5c

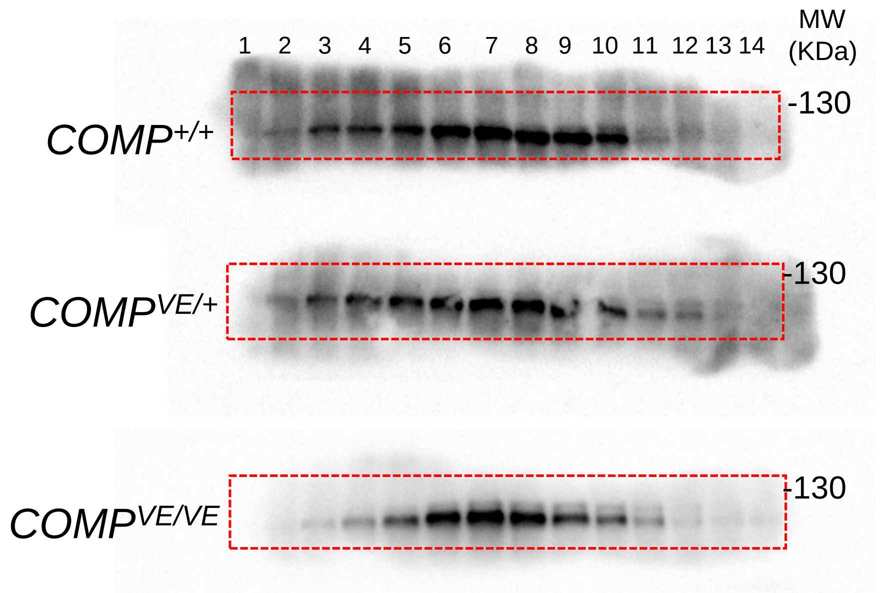

Primary tendon cells

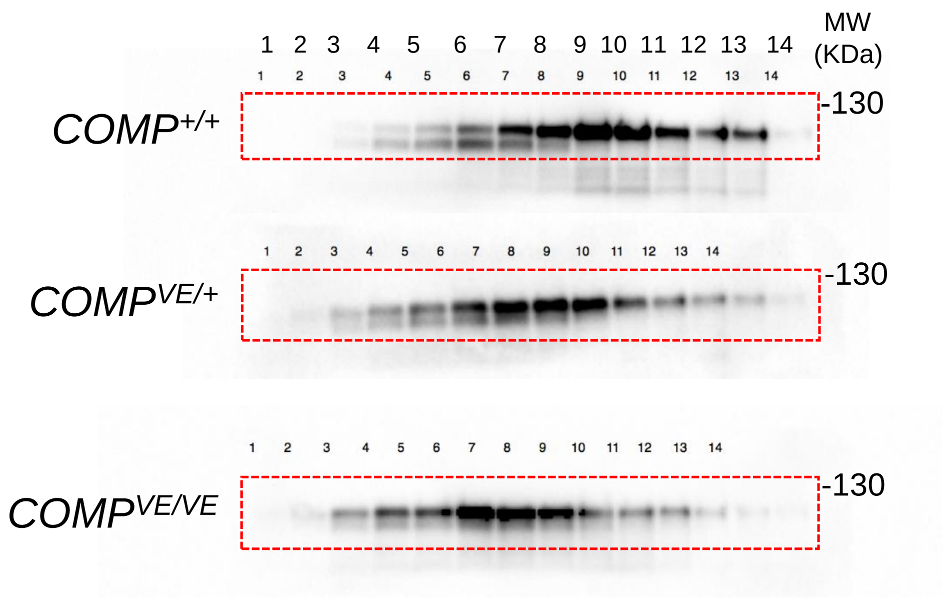

Primary chondrocytes

Figure.5d

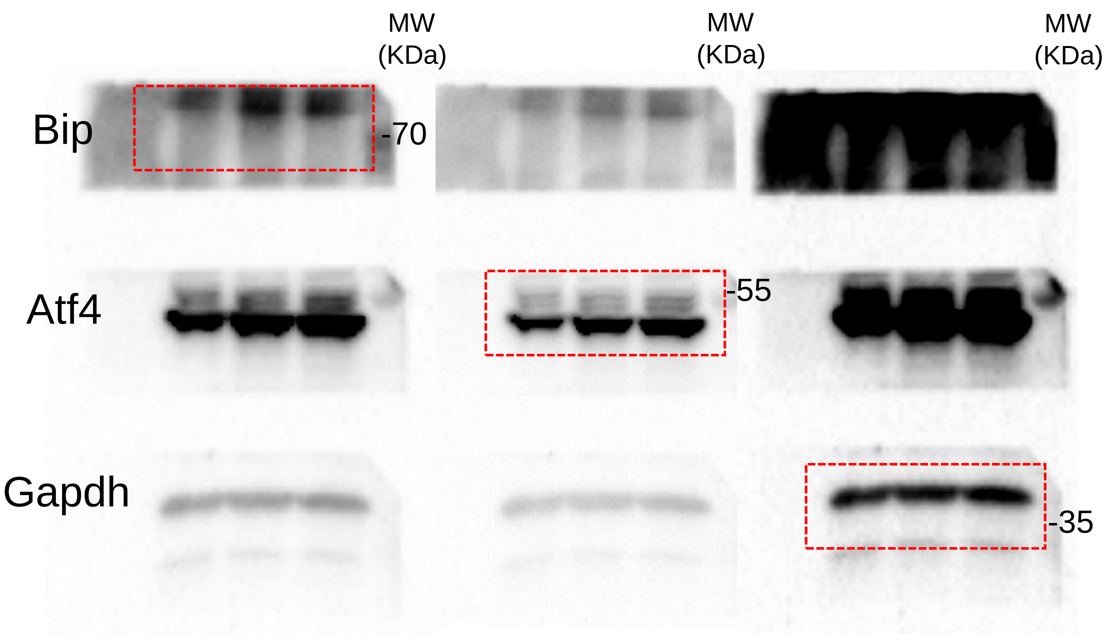

Primary tendon cells

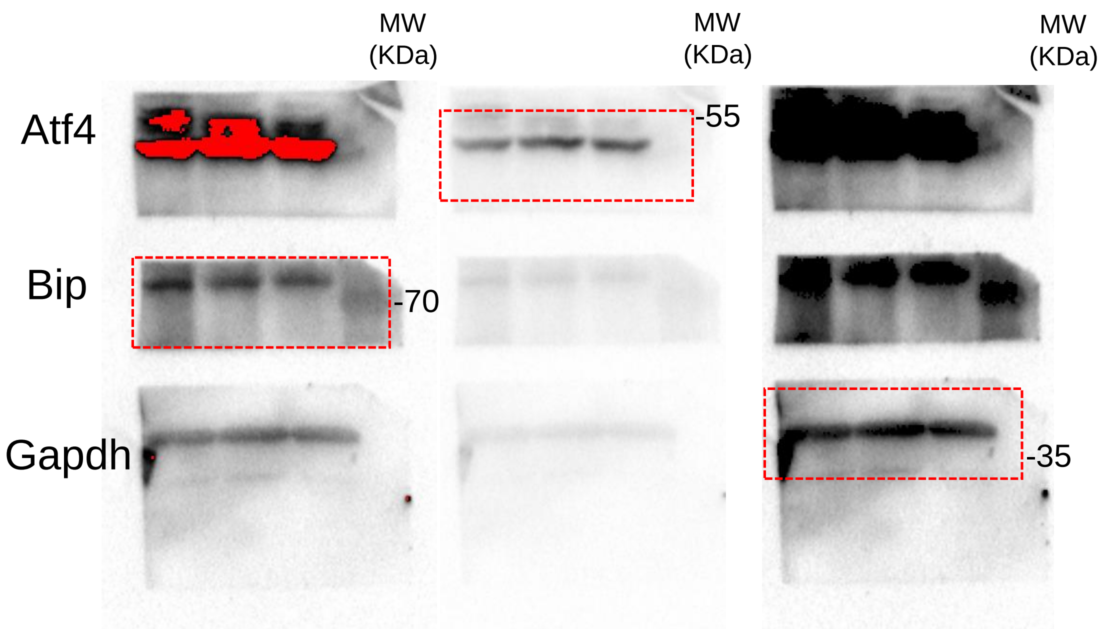

Primary chondrocytes

Uncropped blots of Supplementary Figure 8a, b, c and e

Supplementary Fig.8a

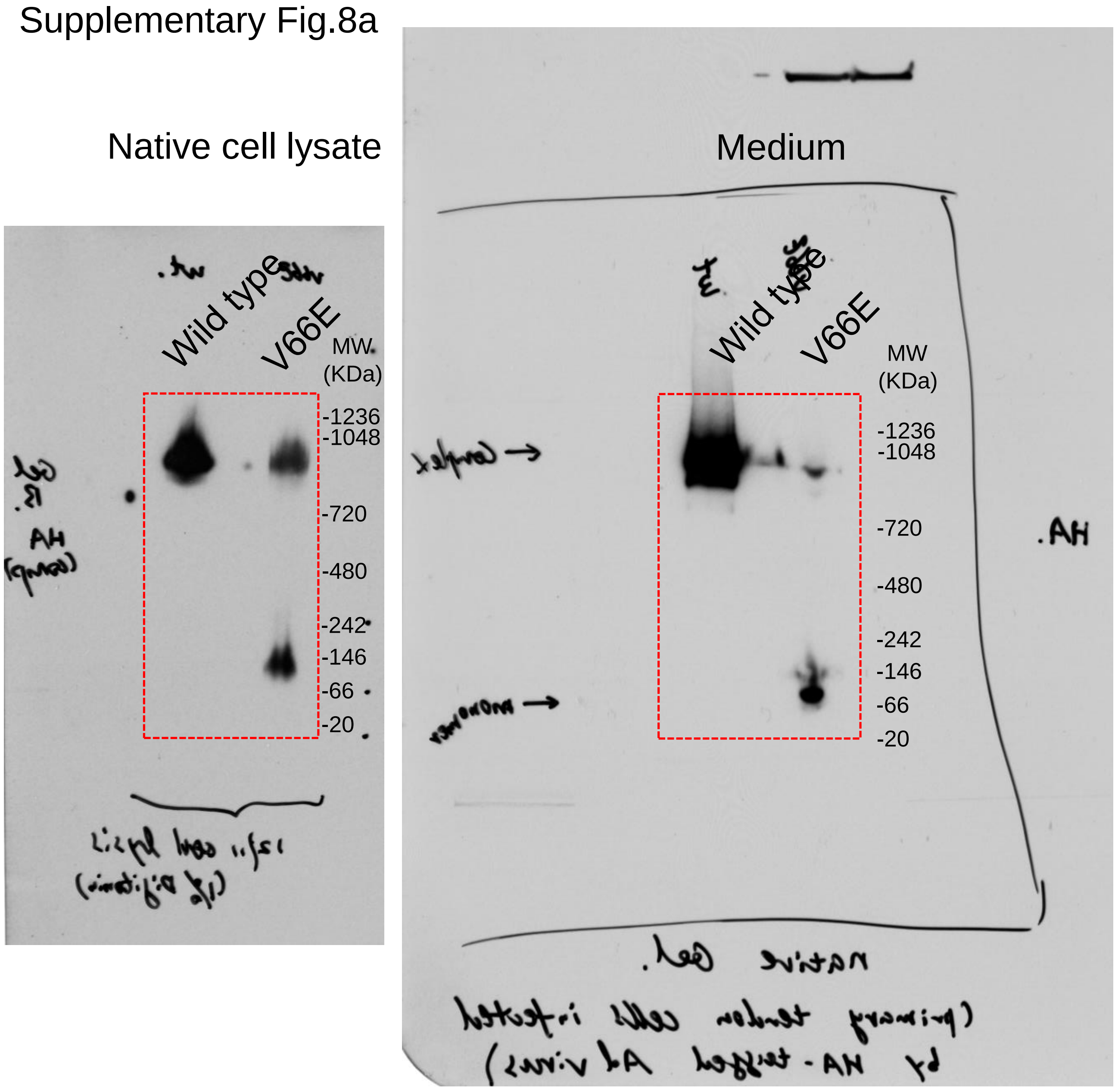

Supplementary Fig.8b

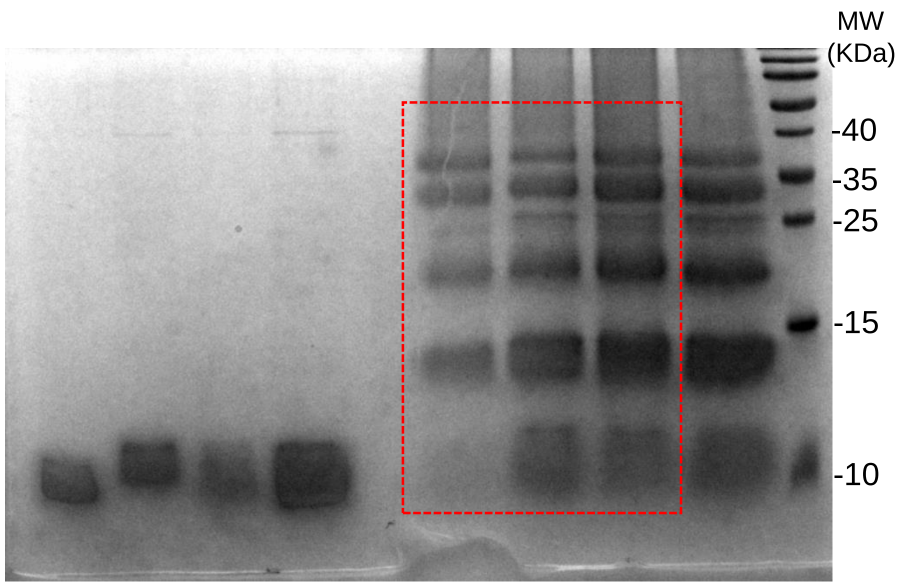

Supplementary Fig.8c

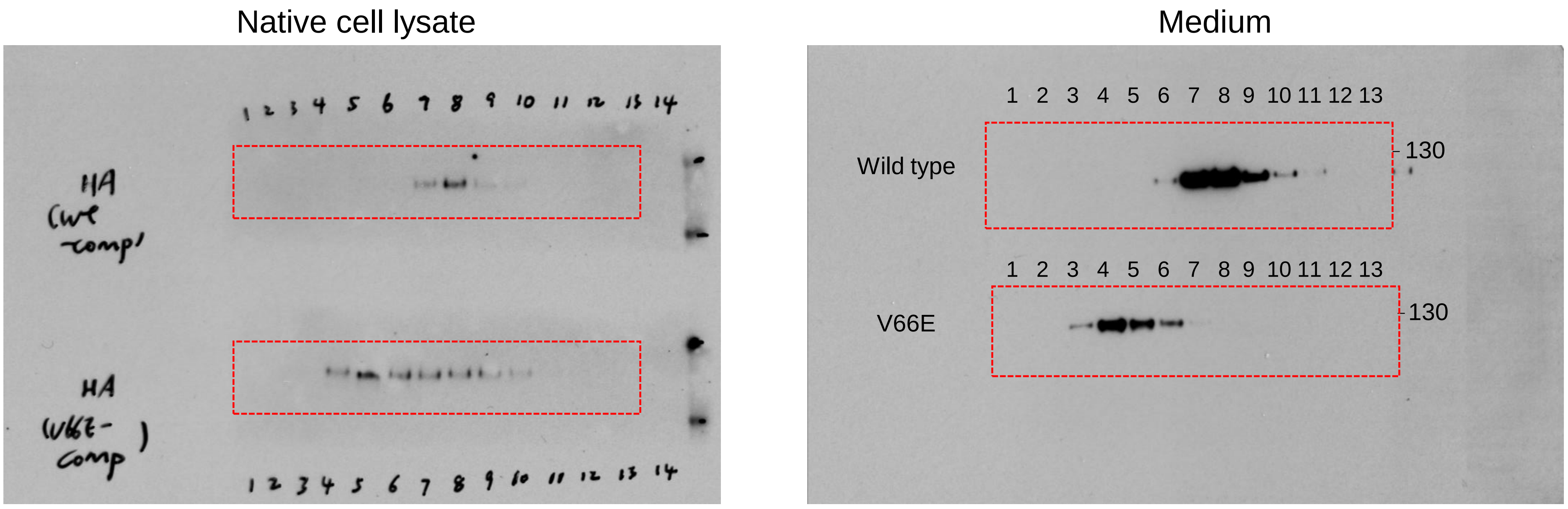

Supplementary Fig.8e

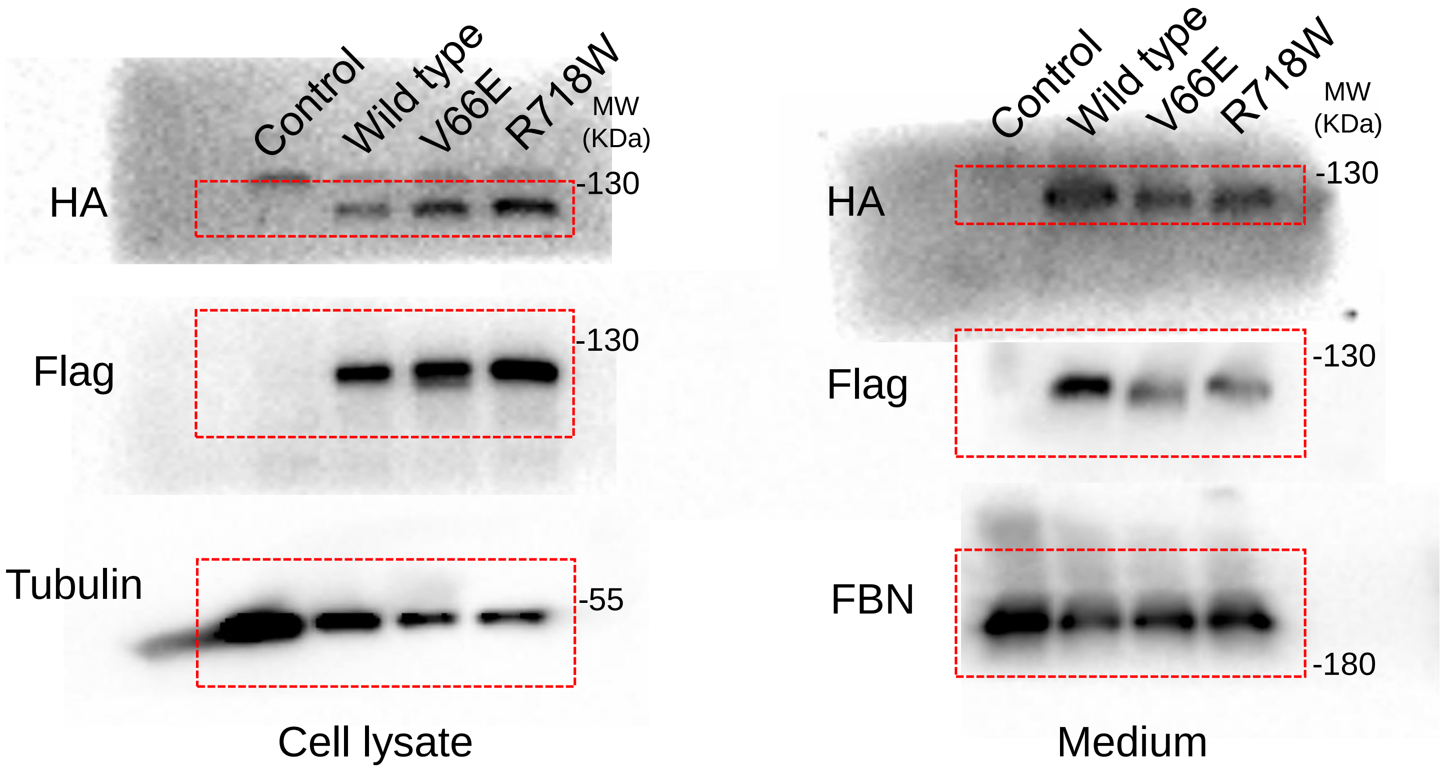

Uncropped blots of Figure 10c, 11c, 12c and 16c

Supplementary Fig.10c

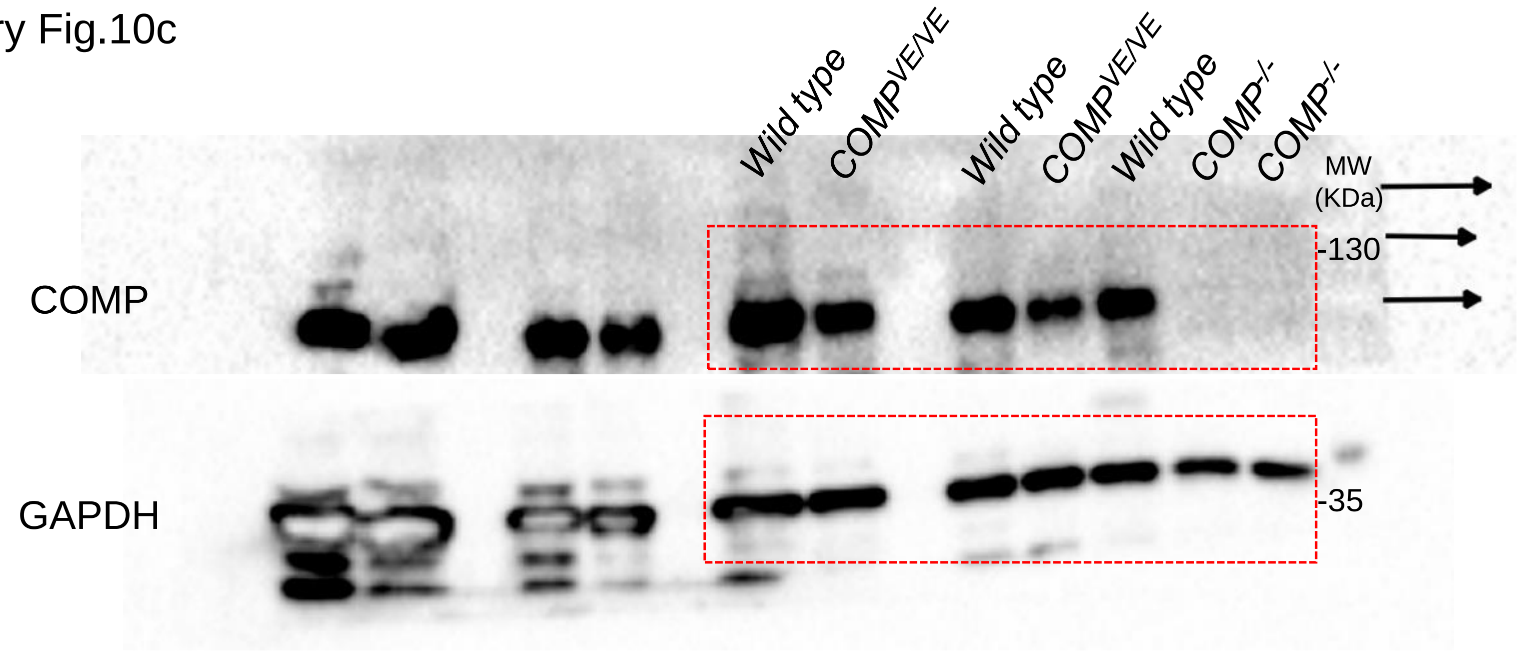

Supplementary Fig.11c

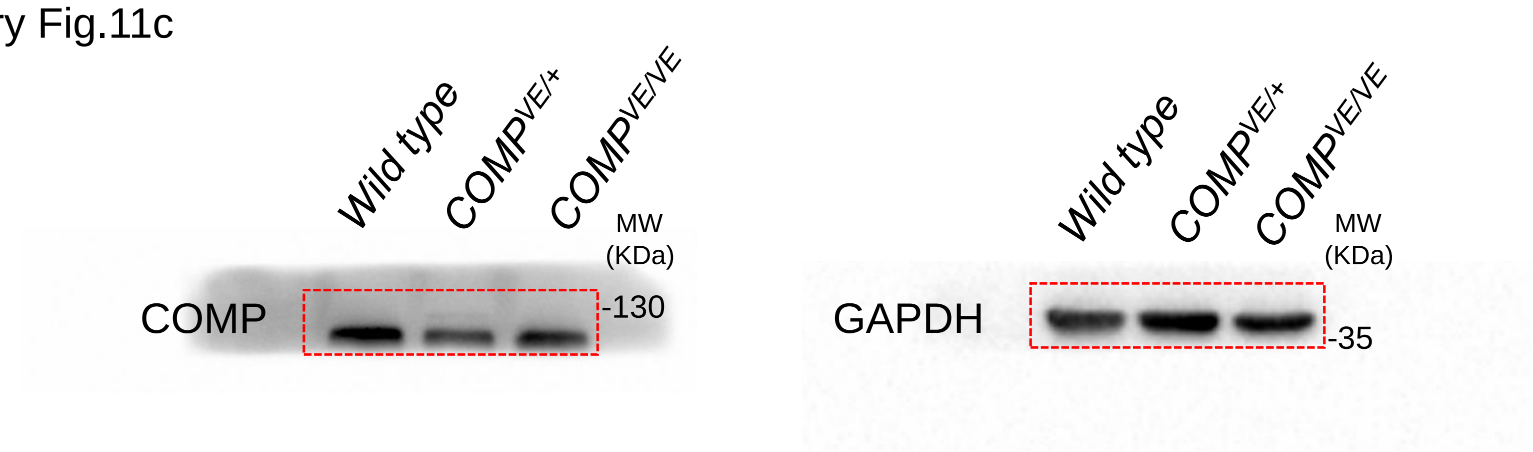

Supplementary Fig.12c

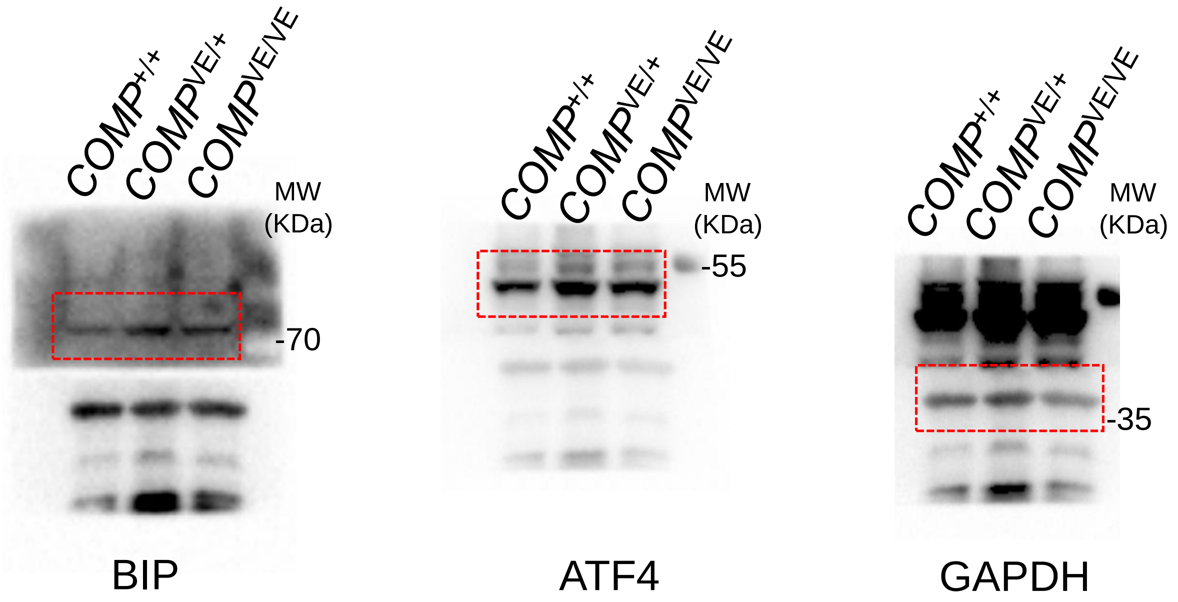

Supplementary Fig.16c

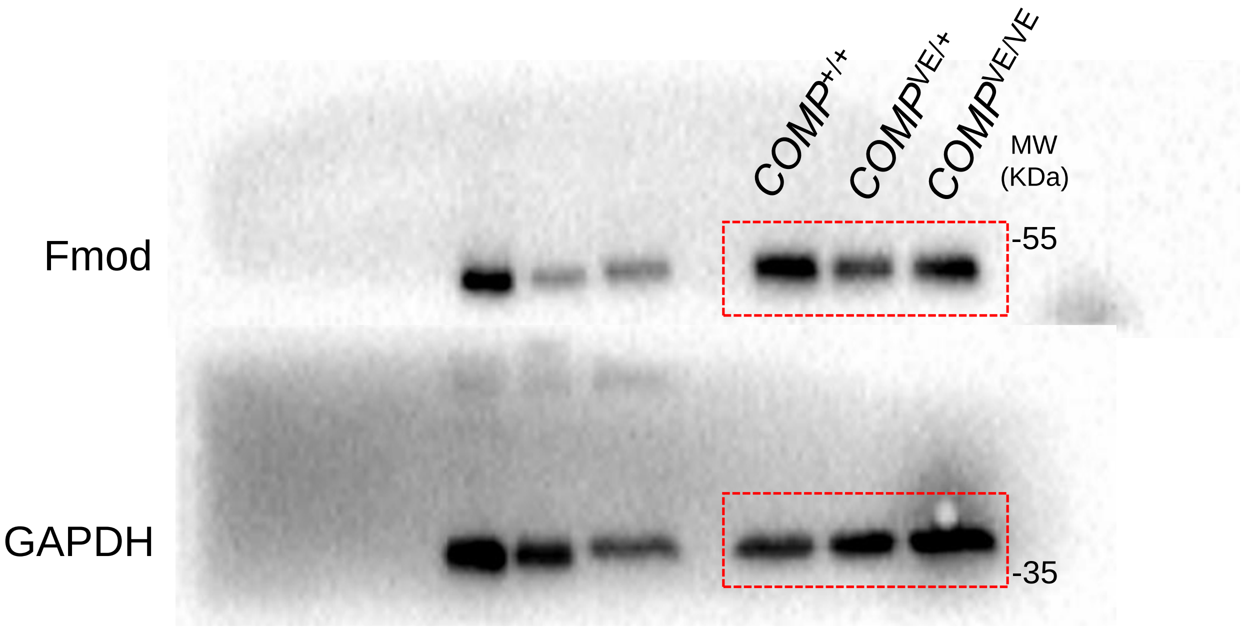

Supplement: Supplementary file 1 — Supplementary Information [file 41467_2020_17378_MOESM1_ESM.pdf]
