# Supplementary material for: Mutations in COMP cause familial carpal tunnel syndrome
Source: Nat Commun. 2020 Jul 20;11:3642. doi: 10.1038/s41467-020-17378-z (PMC7371736; doi:10.1038/s41467-020-17378-z)
Supplement: Supplementary file 2 — Reporting Summary [file 41467_2020_17378_MOESM2_ESM.pdf]

nature research

Corresponding author(s): Bo Gao, Yingzi Yang and Shusen Cui

Last updated by author(s): Jun 5, 2020

Reporting Summary

Nature Research wishes to improve the reproducibility of the work that we publish. This form provides structure for consistency and transparency in reporting. For further information on Nature Research policies, see Authors & Referees and the Editorial Policy Checklist .

Statistics

For all statistical analyses, confirm that the following items are present in the figure legend, table legend, main text, or Methods section.

n/a	Confirmed	
☐	☒	The exact sample size (*n*) for each experimental group/condition, given as a discrete number and unit of measurement
☐	☒	A statement on whether measurements were taken from distinct samples or whether the same sample was measured repeatedly
☐	☒	The statistical test(s) used AND whether they are one- or two-sided<br>*Only common tests should be described solely by name; describe more complex techniques in the Methods section.*
☒	☐	A description of all covariates tested
☐	☒	A description of any assumptions or corrections, such as tests of normality and adjustment for multiple comparisons
☐	☒	A full description of the statistical parameters including central tendency (e.g. means) or other basic estimates (e.g. regression coefficient) AND variation (e.g. standard deviation) or associated estimates of uncertainty (e.g. confidence intervals)
☐	☒	For null hypothesis testing, the test statistic (e.g. *F*, *t*, *r*) with confidence intervals, effect sizes, degrees of freedom and *P* value noted<br>*Give P values as exact values whenever suitable.*
☒	☐	For Bayesian analysis, information on the choice of priors and Markov chain Monte Carlo settings
☒	☐	For hierarchical and complex designs, identification of the appropriate level for tests and full reporting of outcomes
☒	☐	Estimates of effect sizes (e.g. Cohen's *d*, Pearson's *r*), indicating how they were calculated

Our web collection on statistics for biologists contains articles on many of the points above.

Software and code

Policy information about availability of computer code

Data collection	No software was used for data collection.
Data analysis	The following software tools are publicly available and used as described in METHODS:<br>• Linkage analysis: Superlink-SNP 1.1, FASTLINK 4.1P<br>• Sequence data analysis tools: MPG (Most Probable Genotype) and Varsifter (https://research.nhgri.nih.gov/software/VarSifter/)<br>• MRI was performed on a 3.0T MRI scanner (Siemens Skyra VE11) and analyzed by 3D MPR (Multiplanar reconstruction)<br>• MR scanner platform software (SIEMENS-VE11) and RadiAnt DICOM Viewer (SIEMENS-VE40B)<br>• Mouse X-Ray was performed in a Varian linear accelerator 6 MV X-ray machine<br>• TEM sections were scanned by a Philips CM100 transmission electron microscope<br>• TEM firbils distribution was plotted by Prism 6.0<br>• Images were acquired by a Nikon fluorescent microscope or a Zeiss LSM710 confocal microscope<br>• Quantification of images was performed by ImageJ software (version:2.0.0-rc-43/1.50e)<br>• Prism version 6 was used for statistical analysis

For manuscripts utilizing custom algorithms or software that are central to the research but not yet described in published literature, software must be made available to editors/reviewers. We strongly encourage code deposition in a community repository (e.g. GitHub). See the Nature Research guidelines for submitting code & software for further information.

Data

Policy information about availability of data

All manuscripts must include a data availability statement. This statement should provide the following information, where applicable:

- Accession codes, unique identifiers, or web links for publicly available datasets
- A list of figures that have associated raw data
- A description of any restrictions on data availability

All data supporting the findings of this study are available within the paper and its supplementary information files. Raw data and original images of all figures and supplementary figures are included in the Source Data file. All relevant data are available from the authors on reasonable request.

Field-specific reporting

Please select the one below that is the best fit for your research. If you are not sure, read the appropriate sections before making your selection.

- [x] Life sciences
- [ ] Behavioural & social sciences
- [ ] Ecological, evolutionary & environmental sciences

For a reference copy of the document with all sections, see nature.com/documents/nr-reporting-summary-flat.pdf

Life sciences study design

All studies must disclose on these points even when the disclosure is negative.

Sample size	Sample size was as large as possible with at least three independent replicates in critical experiments and the number is sufficient to support the statistical analyses performed in this manuscripts. Only few experiments which are not critical were conducted twice. For mouse studies, we achieved ≥3 mice per genotype per experiment and/or condition. This was based on Mendelian segregation and not any imposed cutoffs. All available human samples from patient families were used, and the sample size from families was not pre-determined. For human tissue samples, we achieved from 2 controls and 3 patients.
Data exclusions	No data were excluded.
Replication	Experimental findings were reliably reproduced. The number of independent biologic replicates is indicated in each Figure Legend.
Randomization	In vitro samples were allocated based on experimental conditions. Mouse samples were allocated based on genotype. All available human samples from patient families were all used for each experiment, and the sample size from families was not pre-determined. Due to the difficulty to obtain age-matched fresh samples from controls, we used all two available samples for each experiment.
Blinding	The investigators were blinded to group allocation during data collection and analysis.

Reporting for specific materials, systems and methods

We require information from authors about some types of materials, experimental systems and methods used in many studies. Here, indicate whether each material, system or method listed is relevant to your study. If you are not sure if a list item applies to your research, read the appropriate section before selecting a response.

Materials & experimental systems

n/a	Involved in the study	
[ ]	[x]	Antibodies
[ ]	[x]	Eukaryotic cell lines
[x]	[ ]	Palaeontology
[ ]	[x]	Animals and other organisms
[ ]	[x]	Human research participants
[x]	[ ]	Clinical data

Methods

n/a	Involved in the study	
[x]	[ ]	ChIP-seq
[x]	[ ]	Flow cytometry
[x]	[ ]	MRI-based neuroimaging

Antibodies

Antibodies used

HA (1:2000 WB, 1:500 IF, Roche, 11867423001)
Flag (1:5000, Sigma, F1804)
COMP (1:2000 WB, 1:500 IHC/IF, 1:10 immuno-gold, GeneTex, GTX14515)
Fibronectin (1:2000 WB, Abcam, Ab2413)
Fibromodulin (1:2000 WB, 1:300 IHC, Provided by Larry Fisher, NIH, LF150)
alpha-Tubulin (1:10000, Abcam, ab7291)
GAPDH (1:2000, Sigma, G8795)

alpha-SMA (1:200, Abcam, ab5694)
alpha-SMA (1:200, Sigma, C6198)
CD11b (1:3000, Abcam, ab133357)
CD68 (1:400, Abcam, ab955)
neutrophil elastase (1:1000, Abcam, ab68672) 9
BIP (1:100 IHC, 1:1000 WB, Enzo Life Sciences, ADI-SPA-826)
ATF4 (1:100 IHC, Sigma, SAB2108508)
ATF4 (1:200 WB, Santa Cruz, sc-200)
CHOP (1:50, Santa Cruz, sc-575)
cleaved caspase 3 (1:100, Cell Signaling, 9961L)
KDEL (1:200, Abcam, ab12223)
Perilipin (1:300, Cell Signaling, 9349S)
CD34 (1:200, Abcam, ab81289)
Tenomodulin, tnmd (1:100, Abcam, ab203676)
type I Collagen (1:10 immuno-gold, Abcam, ab34710)
type III Collagen (1:500 IHC, 1:10 immuno-gold, Abcam, ab7778)
12/101 (1:50, DSHB)
FSP1 (1:200, Abcam, ab41532)
HRP-conjugated mouse (1:10000, GE Healthcare, NA9310)
HRP-conjugated mouse (1:200, Dako, P0161)
Biotinylated anti-Rabbit (1:50, Dako, E0432)
HRP-conjugated rabbit (1:10000, GE Healthcare, NA9340)
HRP-conjugated rabbit (1:200, Dako, P0260)
HRP-conjugated rat (1:10000, Sigma, AP136P)
Alexa Fluor 488 donkey anti rabbit IgG (1:500, Invitrogen, A32790)
Alexa Fluor 568 donkey anti rabbit IgG (1:500, Invitrogen, A10042)
Alexa Fluor 568 donkey anti mouse IgG (1:500, Invitrogen, A10037)

Validation

HA (Roche, 11867423001), validation reference PMID: 21911455
Flag (Sigma, F1804), validation reference PMID: 28242625
COMP (GeneTex, GTX14515), validation reference PMID: 23956175
Fibronectin (Abcam, Ab2413), validation reference PMID: 27857162
Fibromodulin (Provided by Larry Fisher, NIH, LF150), validation reference PMID: 21294898
alpha-Tubulin (Abcam, ab7291), validation reference PMID: 29456190
GAPDH (Sigma, G8795), validation reference PMID: 21841012
alpha-SMA (Abcam, ab5694), validation reference PMID: 27964754
alpha-SMA (Sigma, C6198), validation reference PMID: 18539926
CD11b (Abcam, ab133357), validation reference PMID: 27892938; 27194729
CD68 (Abcam, ab955), validation reference PMID: 28205524
neutrophil elastase (Abcam, ab68672), validation reference PMID: 27752109
BIP (Enzo Life Sciences, ADI-SPA-826) , validation reference PMID: 30024379
ATF4 (Sigma, SAB2108508), validation reference PMID: 30024379
ATF4 (Santa Cruz, sc-200), validation reference PMID: validated by WT using K-562 and Jurkat cells (SCBT website)
CHOP (Santa Cruz, sc-575), validation reference PMID: validated by IHC using human breast tumor (SCBT website)
cleaved caspase 3(Cell Signaling, 9961L), validated by IF using HT-29 cells (CST website)
KDEL (Abcam, ab12223), validation reference PMID: 24465392; 29416295
Perilipin (Cell Signaling, 9349S), validated by IF using mouse brown adipose tissue and 3T3-L1 cells (CST website)
CD34 (Abcam, ab81289), validation reference PMID: 28769083
Tenomodulin (Abcam, ab203676), validation reference PMID: 27832770; 29286087
type I Collagen (Abcam, ab34710), validation reference PMID: 26136428; 29605718
type III Collagen (Abcam, ab7778), validation reference PMID: 28638180
12/101 (DSHB), validation reference PMID: 22954963
FSP1 (Abcam, ab41532), validation reference PMID: 21889495
HRP-conjugated mouse (GE Healthcare, NA9310), validation based on manufacturer’s data sheet
HRP-conjugated mouse (Dako, P0161), validation based on manufacturer’s data sheet
Biotinylated anti-Rabbit (Dako, E0432), validation based on manufacturer’s data sheet
HRP-conjugated rabbit (GE Healthcare, NA9340), validation based on manufacturer’s data sheet
HRP-conjugated rabbit (Dako, P0260), validation based on manufacturer’s data sheet
HRP-conjugated rat (Sigma, AP136P), validation based on manufacturer’s data sheet
Alexa Fluor 488 donkey anti rabbit IgG (Invitrogen, A32790), validation based on manufacturer’s data sheet
Alexa Fluor 568 donkey anti rabbit IgG (Invitrogen, A10042), validation based on manufacturer’s data sheet
Alexa Fluor 568 donkey anti mouse IgG (Invitrogen, A10037), validation based on manufacturer’s data sheet

Eukaryotic cell lines

Policy information about cell lines

Cell line source(s)	Rat chondrosarcoma cell (B de Crombrugghe), HEK293 cell line (ATCC)
Authentication	None of the cell lines used were authenticated.
Mycoplasma contamination	Cells were routinely tested for mycoplasma contamination in the cell culture facility. All are negative.
Commonly misidentified lines (See ICLAC register)	No commonly misidentified cell lines were used.

Animals and other organisms

Policy information about studies involving animals; ARRIVE guidelines recommended for reporting animal research

Laboratory animals	Scx-GFP mouse, V65E-COMP mouse, and COMP null mouse lines were maintained in system water according to standard methods. Mice (Mus musculus) were housed in the Minimal Disease Area, Laboratory Animal Unit, Li Ka Shing Faculty of Medicine, The University of Hong Kong. They were housed in randomized groups of maximum five, at 22°C with a 12-hour light-dark cycle, and were fed a conventional diet. All strains were maintained on a mixed C57BL/6J background. Male and female were both included in random numbers per experiment.
Wild animals	No wild animals were used in the study.
Field-collected samples	No field collected samples were used in the study.
Ethics oversight	Mice were maintained according to the approved protocol of Committee on the Use of Live Animals in Teaching and Research (CULATR), The University of Hong Kong

Note that full information on the approval of the study protocol must also be provided in the manuscript.

Human research participants

Policy information about studies involving human research participants

Population characteristics	Initial CTS symptoms in most affected Family 1 members occurred at 20-30 years of age, while the age of onset of Family 2 is between 30s and 50s. Patients and controls were recruited from the China-Japan Union Hospital of Jilin University, China.
Recruitment	All CTS patients were diagnosed by experienced hand surgeons, some of the patients and controls were further examined by both hand surgeons and orthopedists. Patient diagnosis was conducted based on medical history, physical examination, and nerve conduction tests. Clinical characteristics of patients were collected. Part of patients and controls were further examined by MRI. MRI images were obtained from patients and controls (unaffected family members and irrelevant normal human). Solid tissues (transverse carpal ligaments, digital flexor tendons, and subsynovial connective tissues) were obtained from three Family 1 patients and two controls (amputees without relevant CTS symptoms). Specimens were sectioned and stored at room temperature. Informed consents were obtained from all the participants.
Ethics oversight	Ethical oversight was provided by the Institutional Review Board (IRB) of the China-Japan Union Hospital of Jilin University, China, the National Human Genome Research Institute, National Institutes of Health (NIH), U.S.A, and the University of Hong Kong, Hong Kong SAR, China.

Note that full information on the approval of the study protocol must also be provided in the manuscript.
