# Supplementary material for: Mutations in COMP cause familial carpal tunnel syndrome
Source: Nat Commun. 2020 Jul 20;11:3642. doi: 10.1038/s41467-020-17378-z (PMC7371736; doi:10.1038/s41467-020-17378-z)

Fig.1b

Control

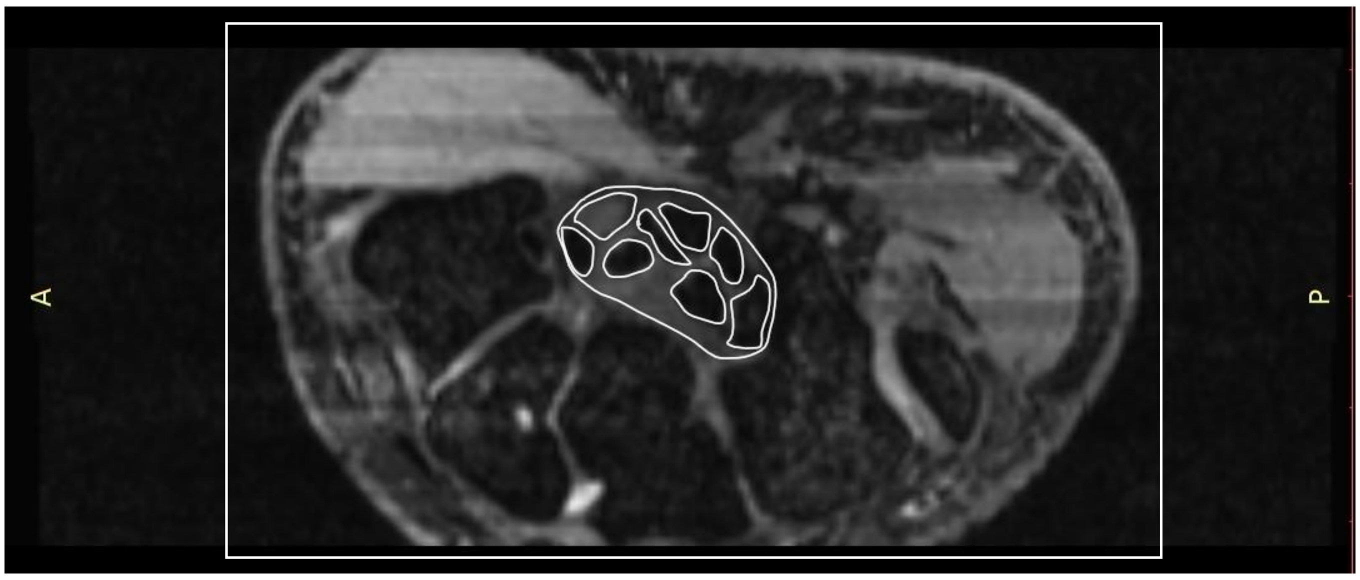

CTS patient

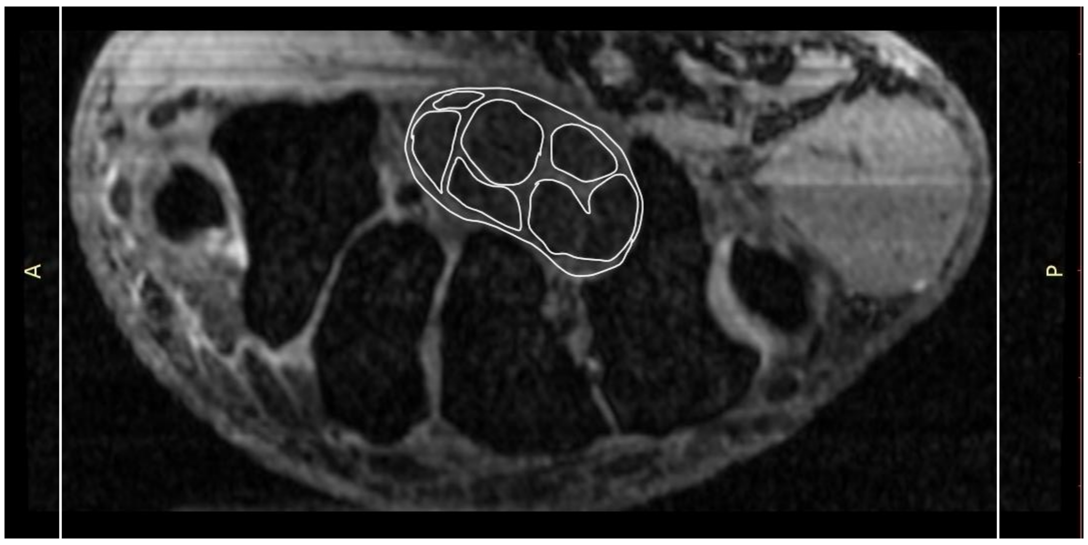

Fig.1c

Control

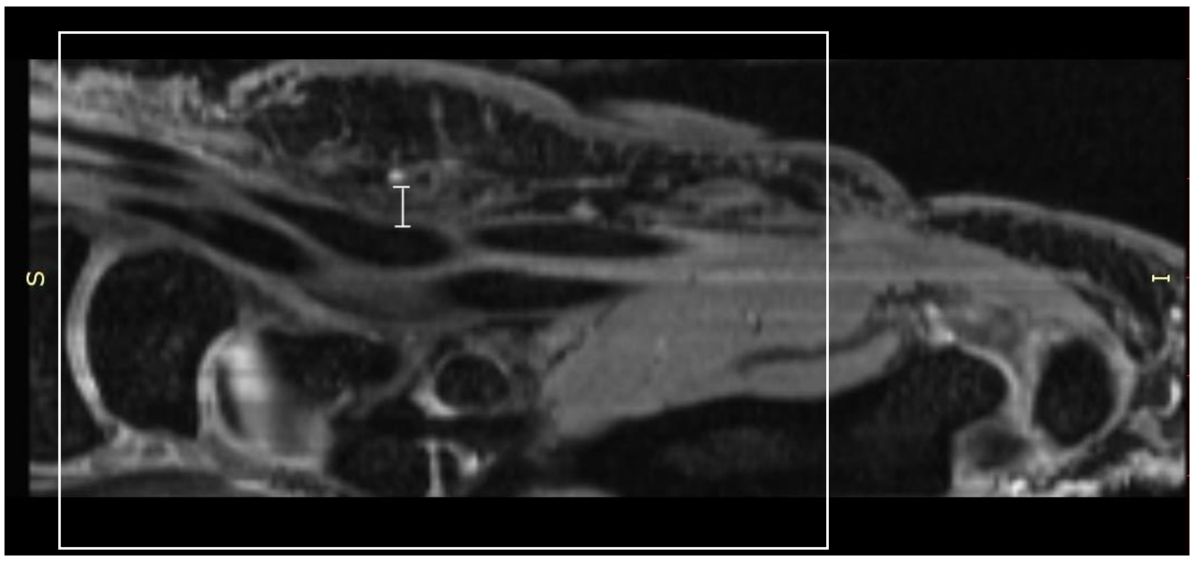

CTS patient

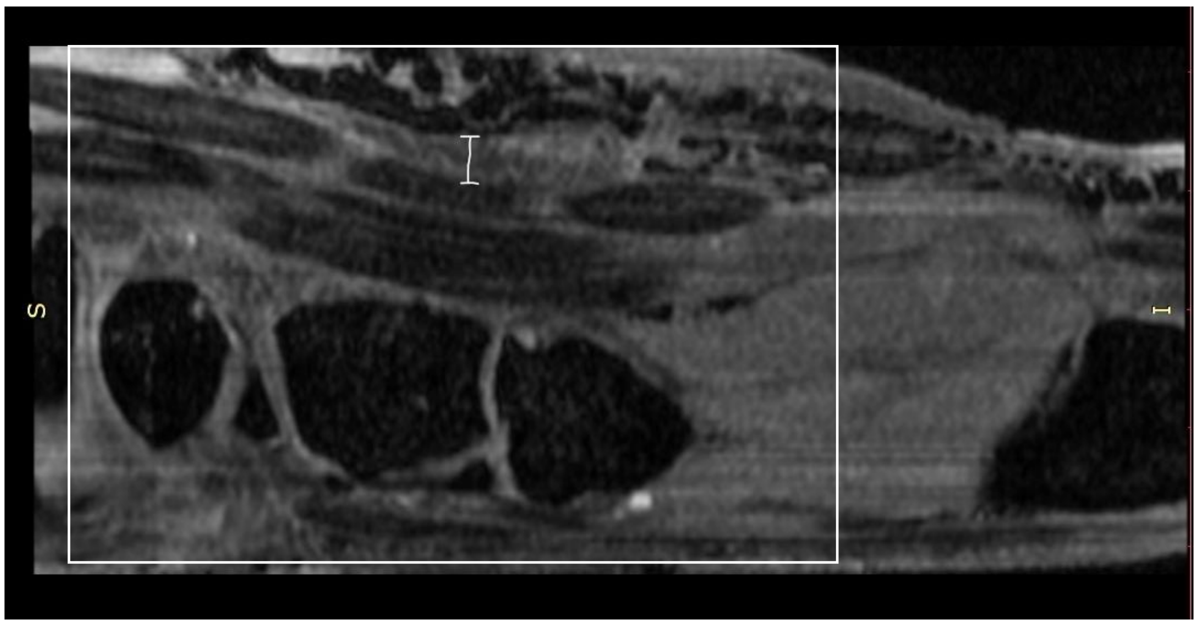

Fig.2a

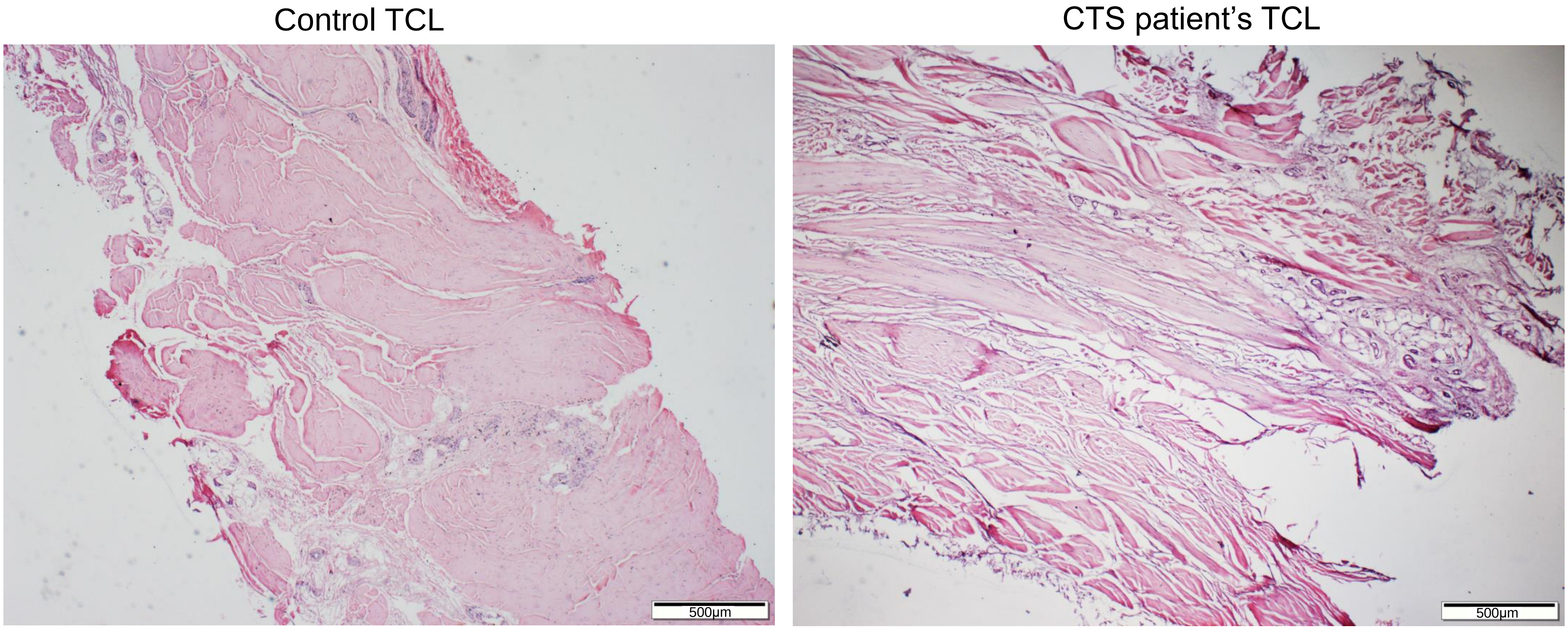

Fig.2b-d

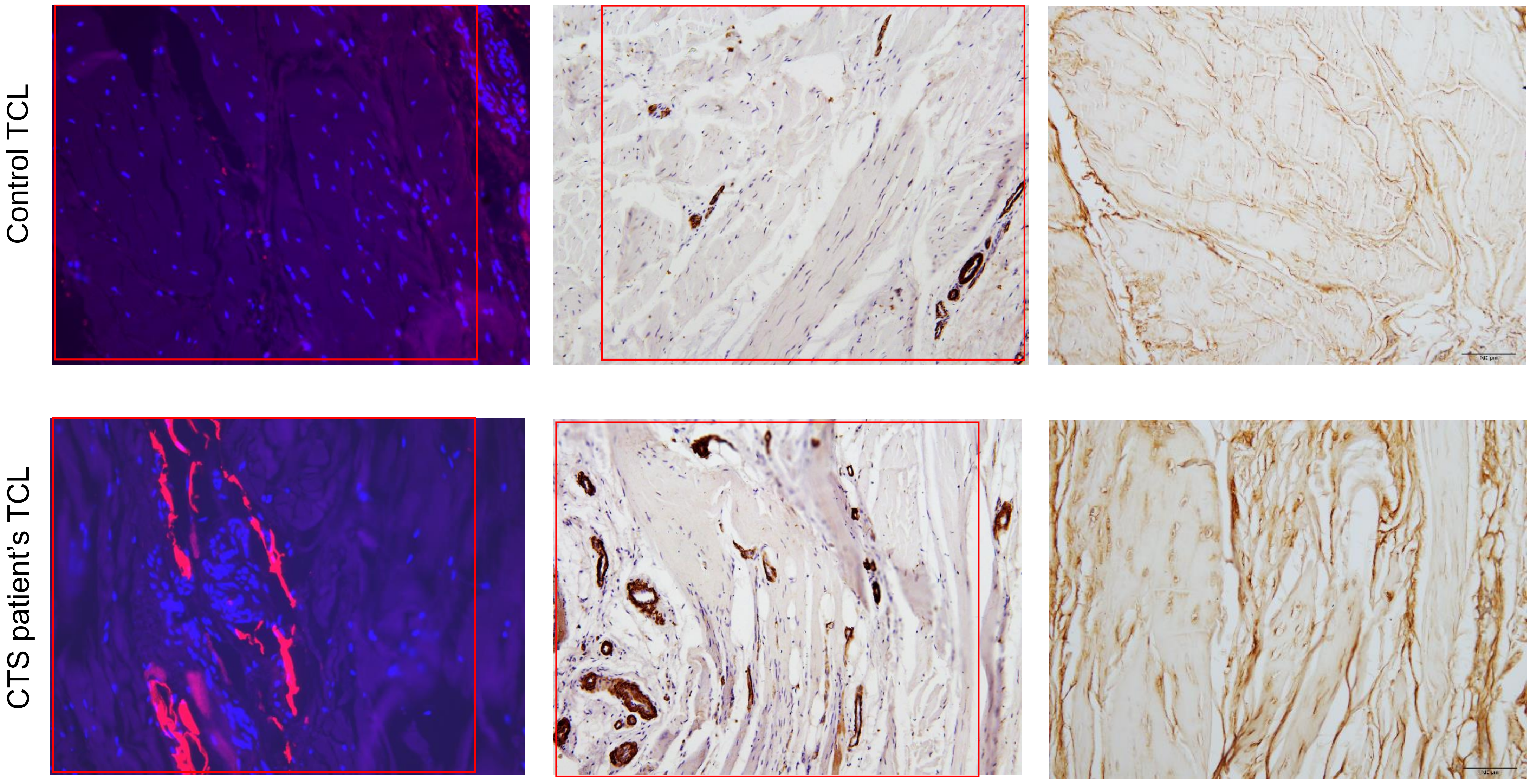

Fig.2e

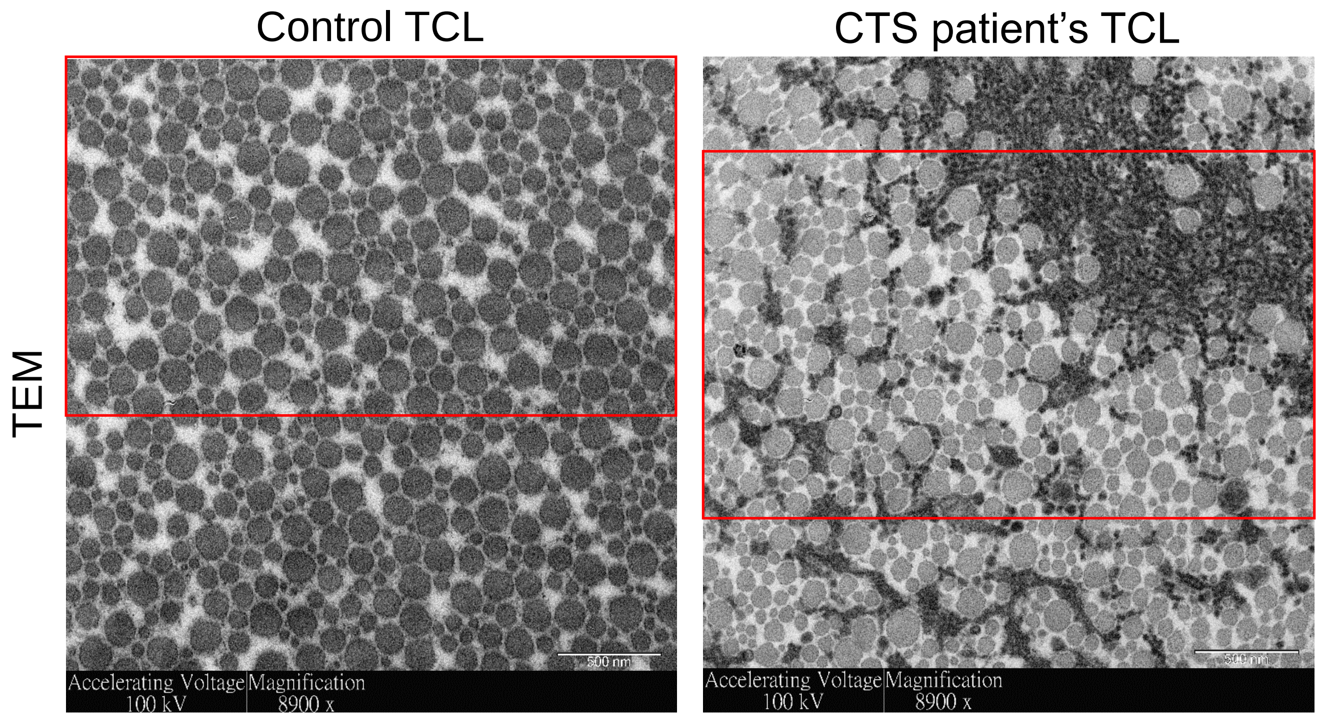

Fig.4b

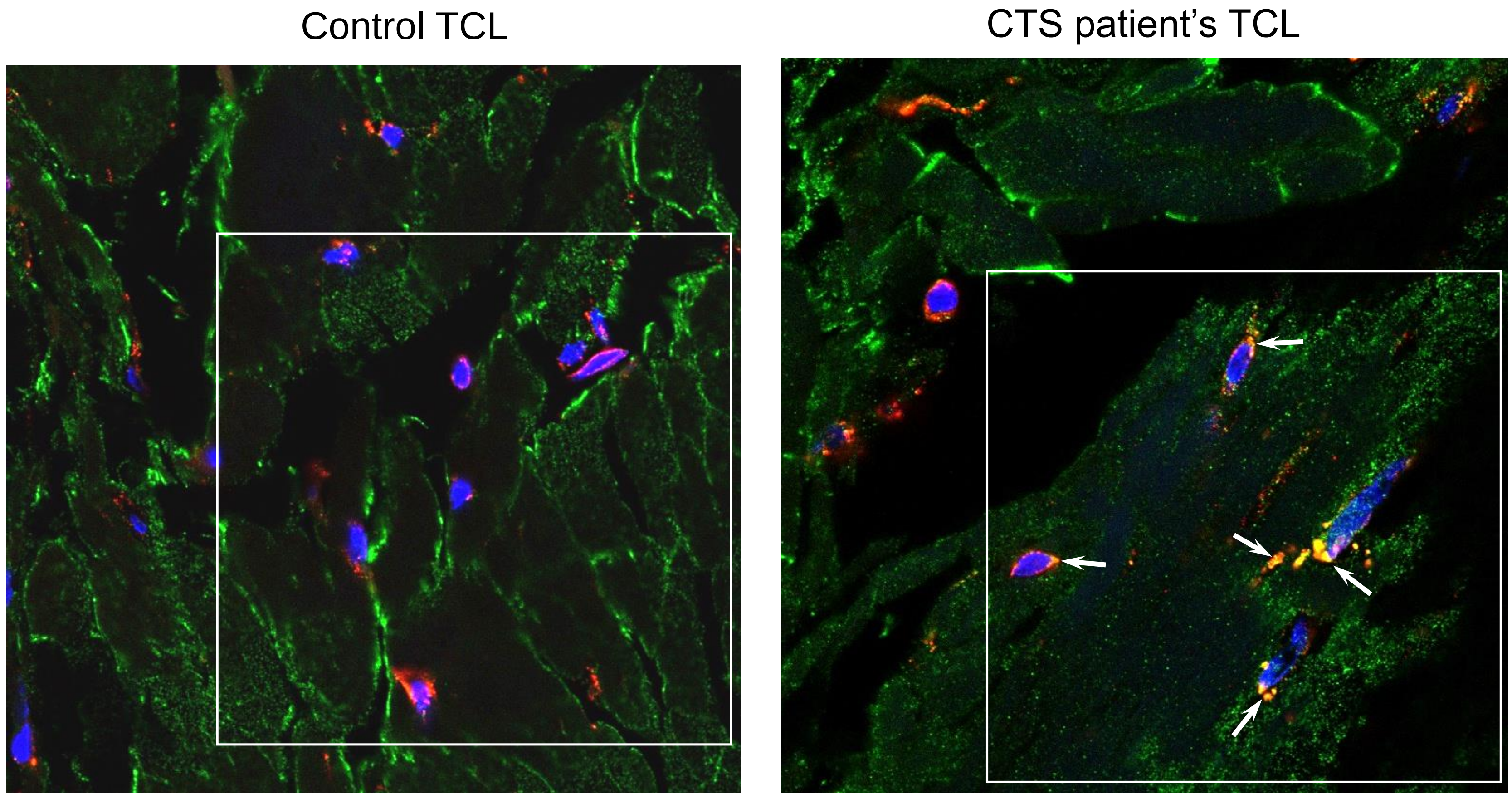

Fig.4c

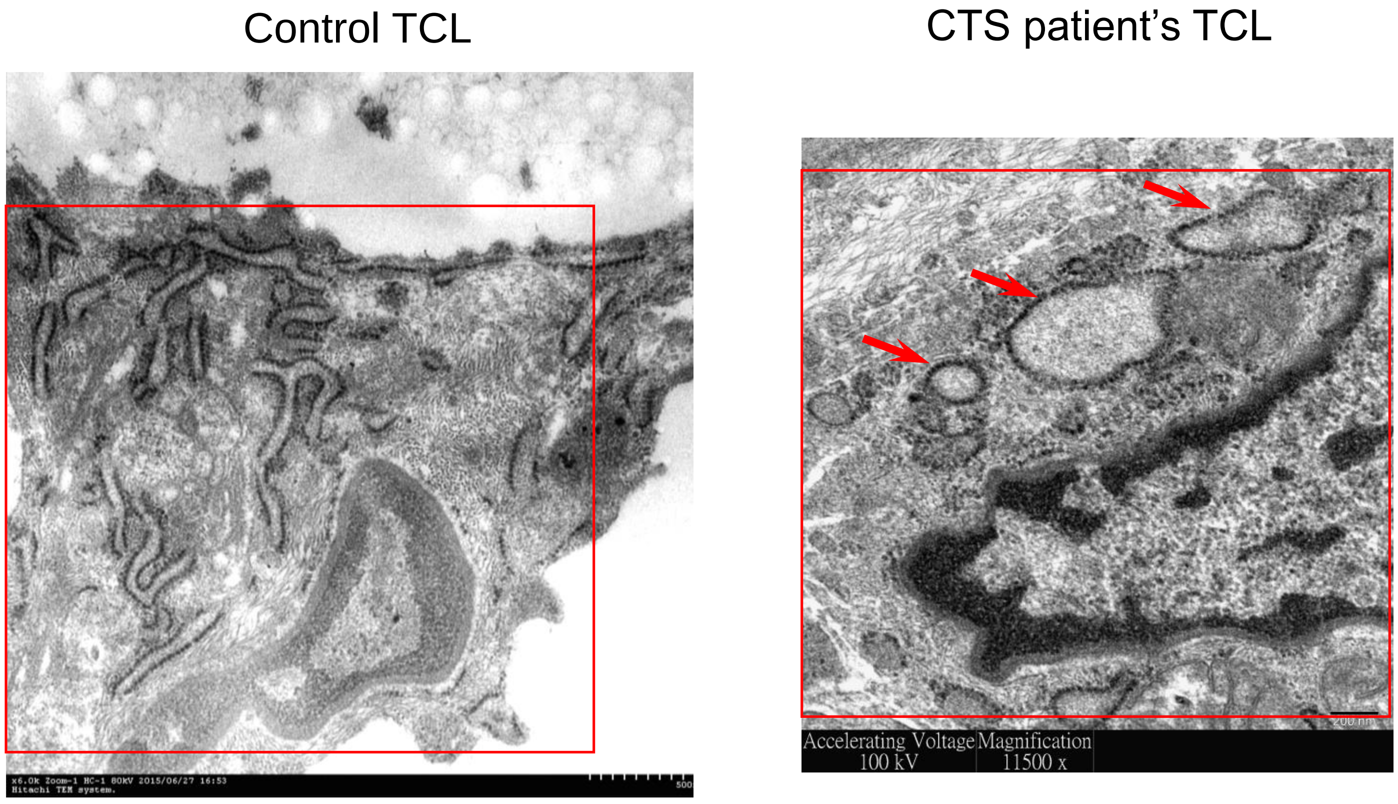

Fig.4d

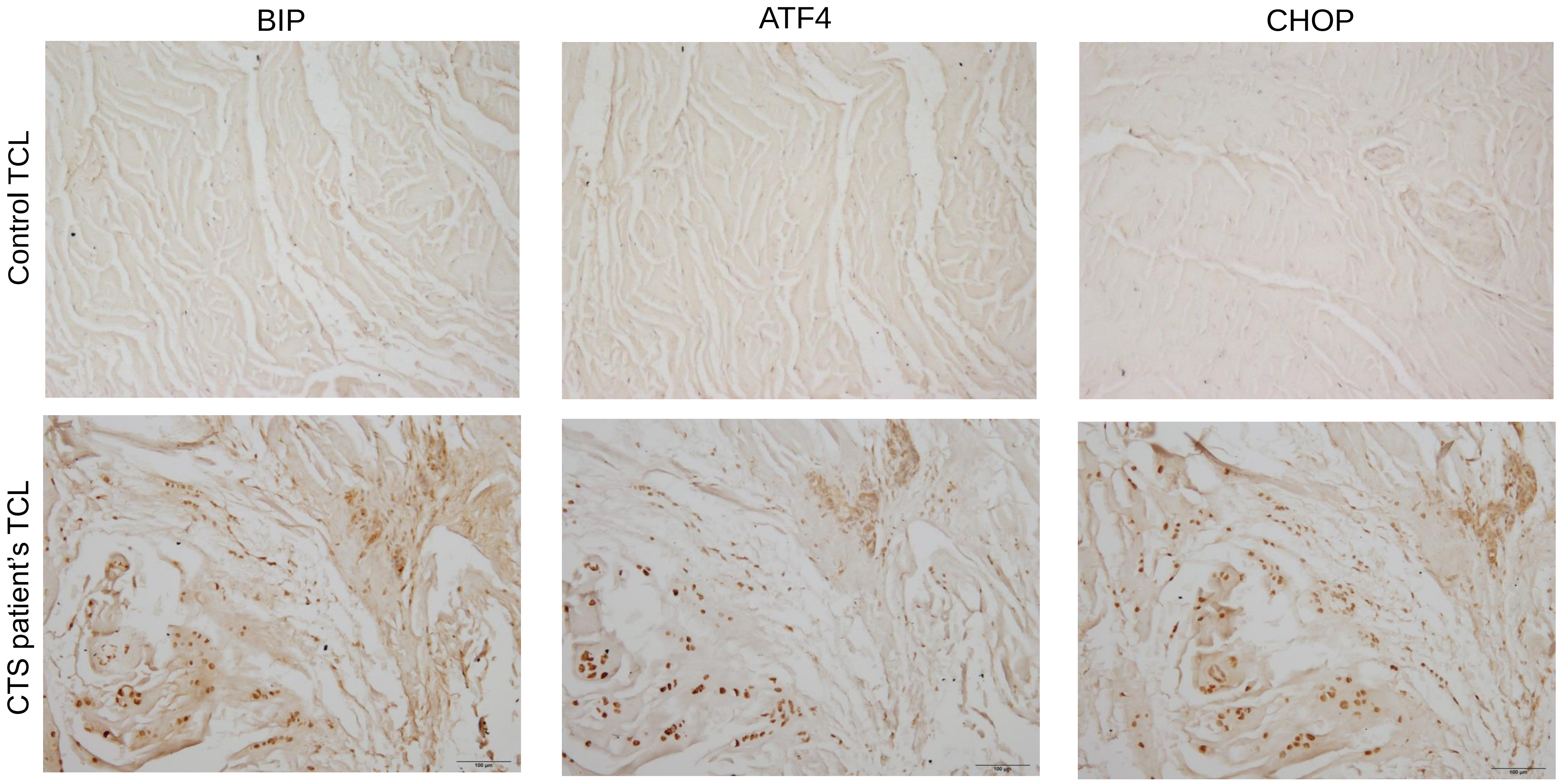

Fig.6a

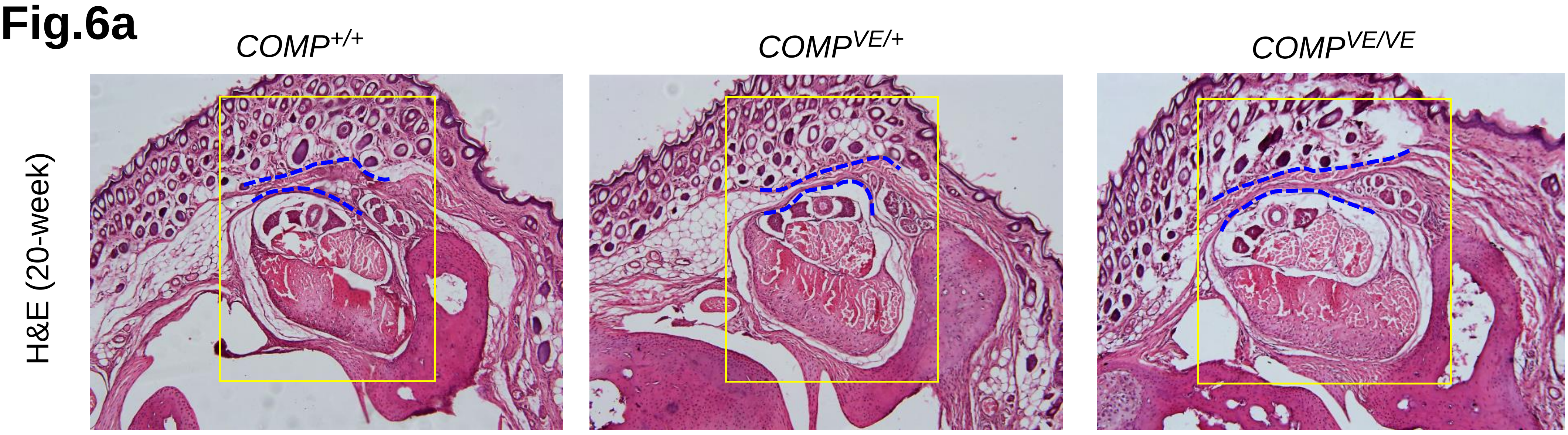

Fig.6c

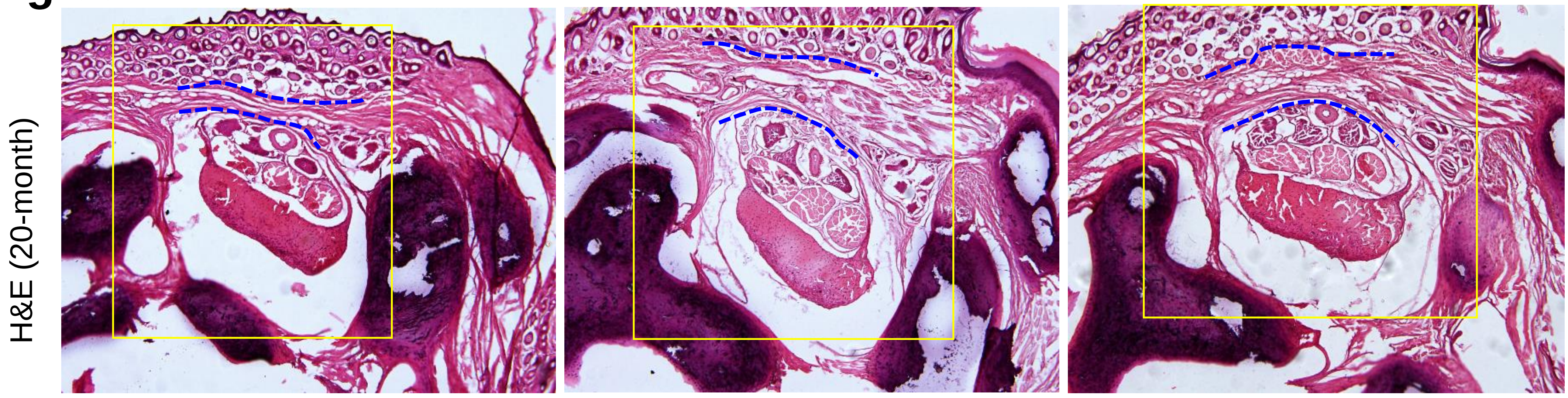

Fig.6d

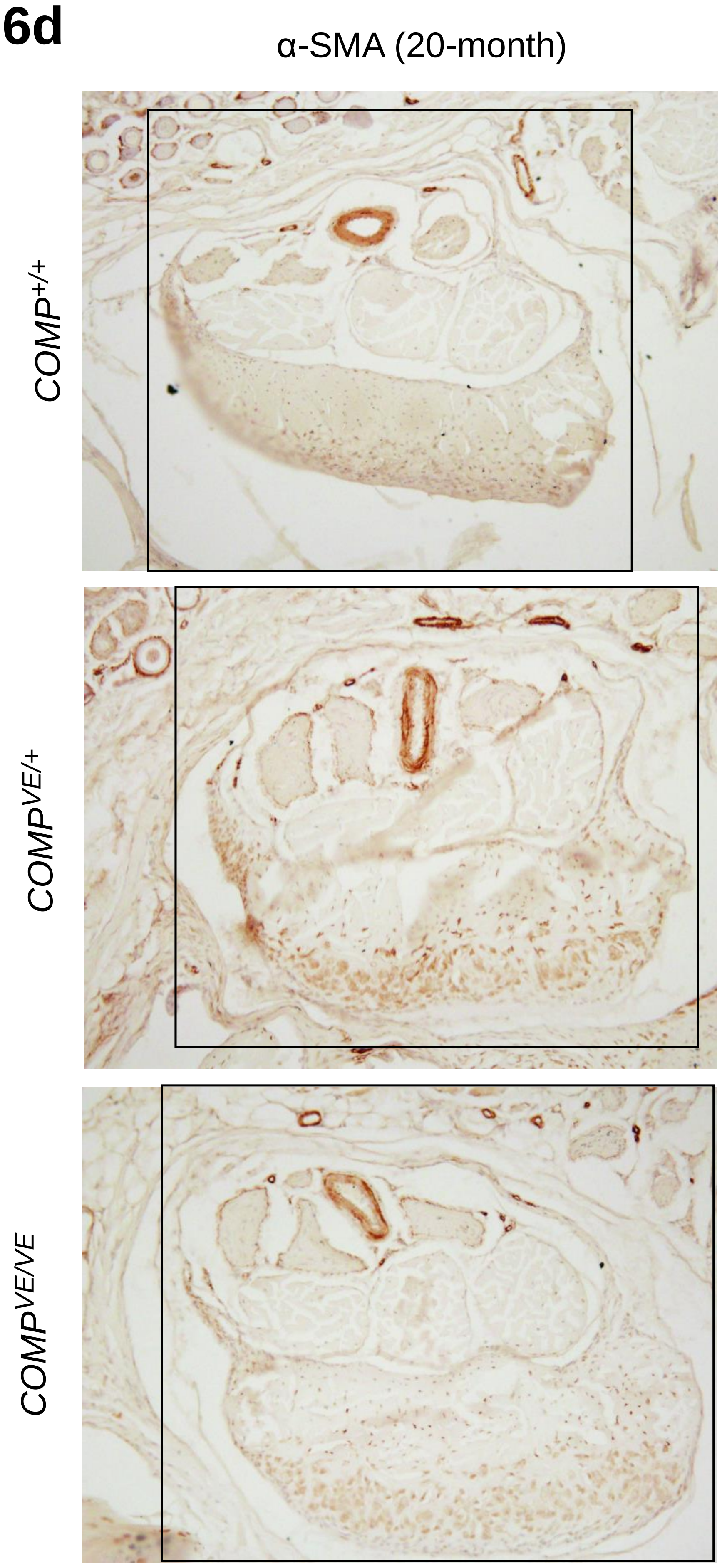

Fig.6e

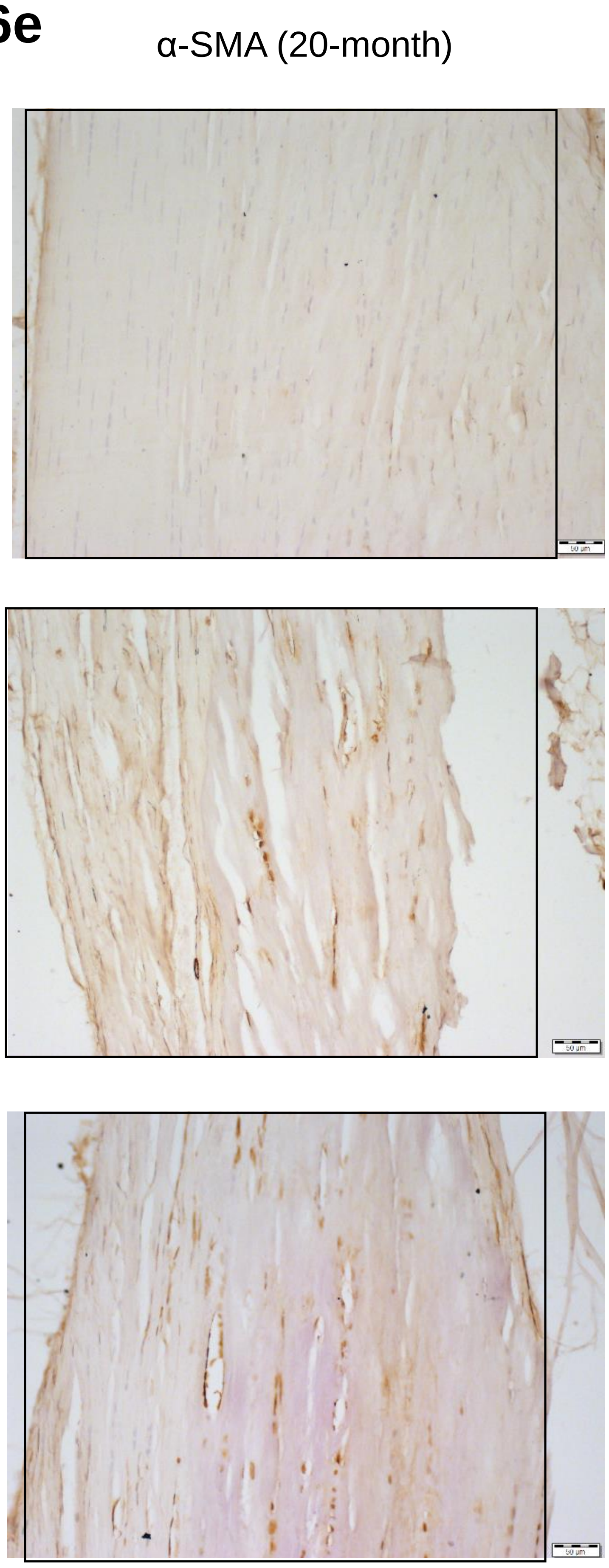

Fig.7a

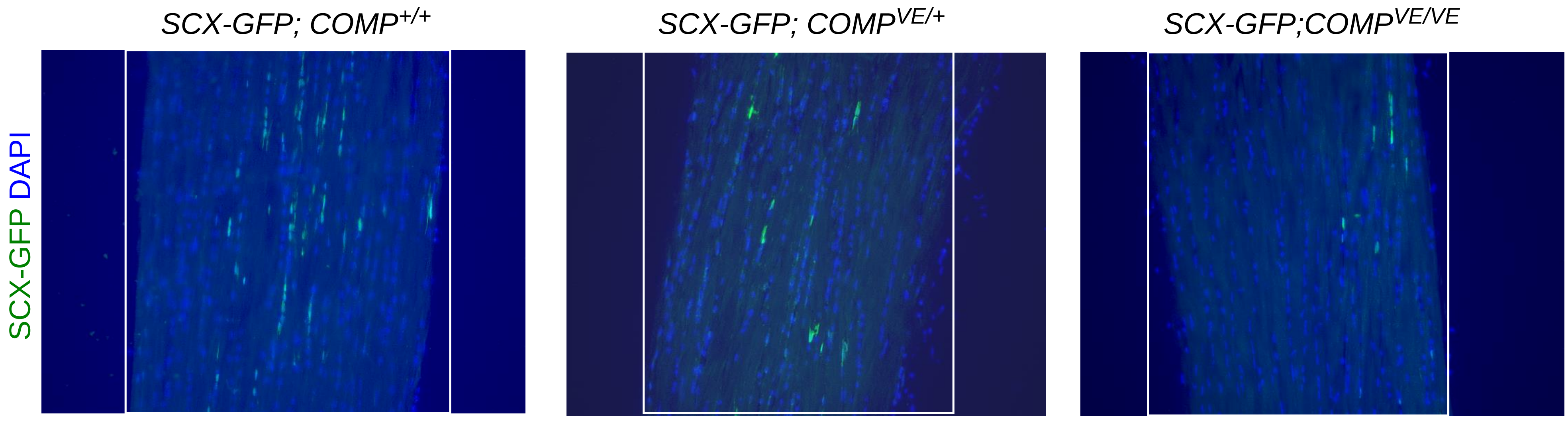

Fig.7c

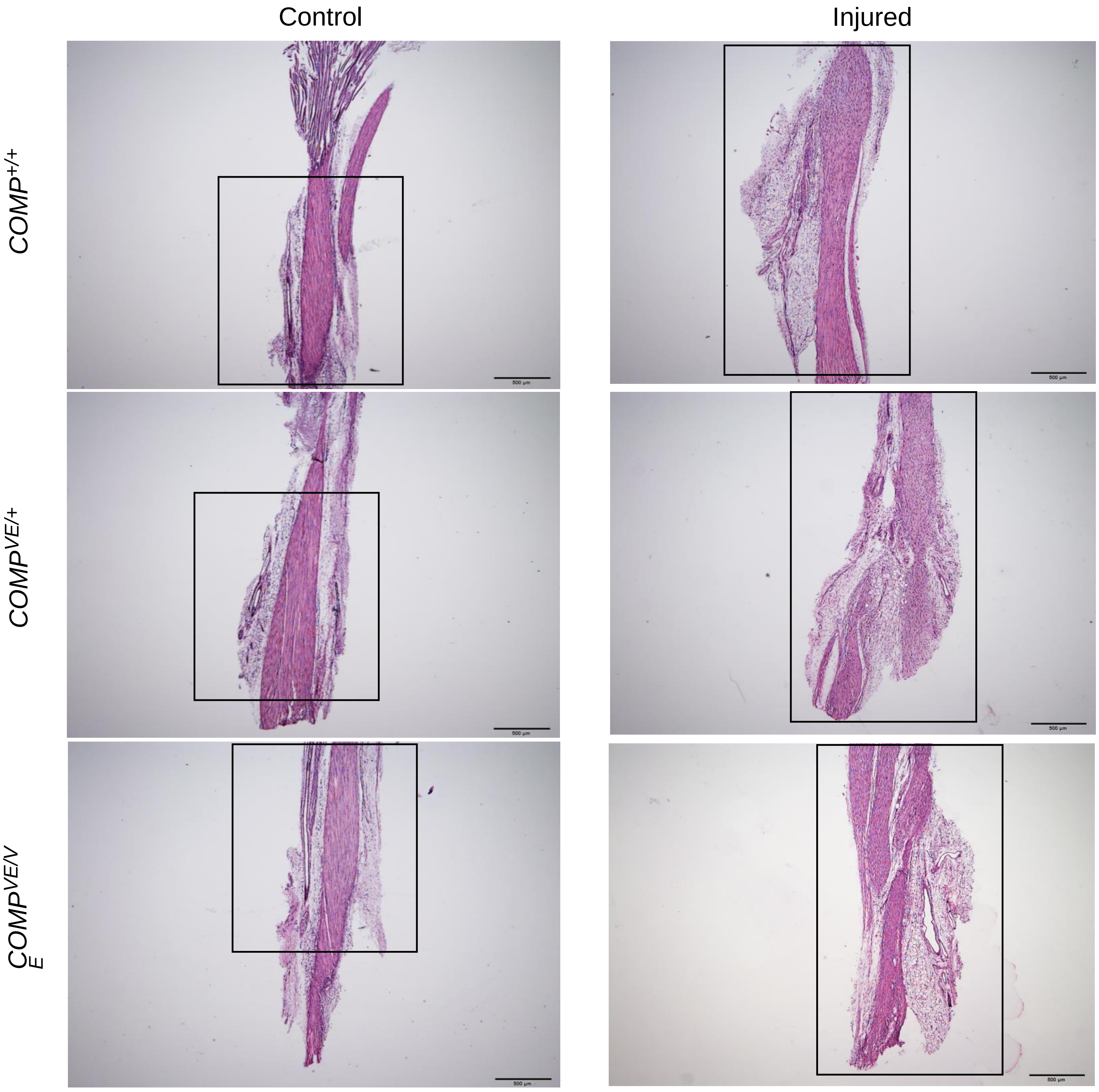

Fig.7d

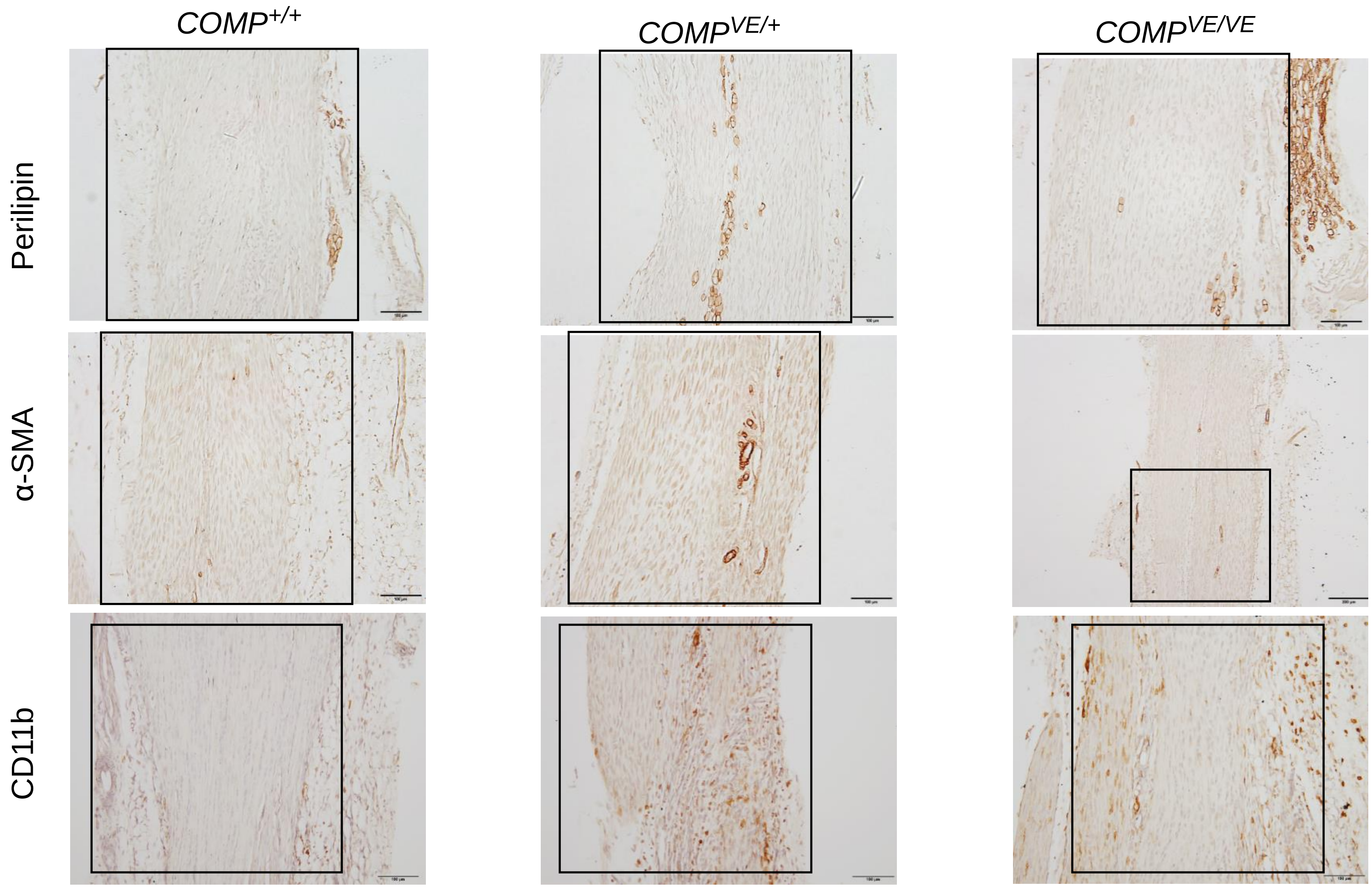

Supplementary Fig.2b

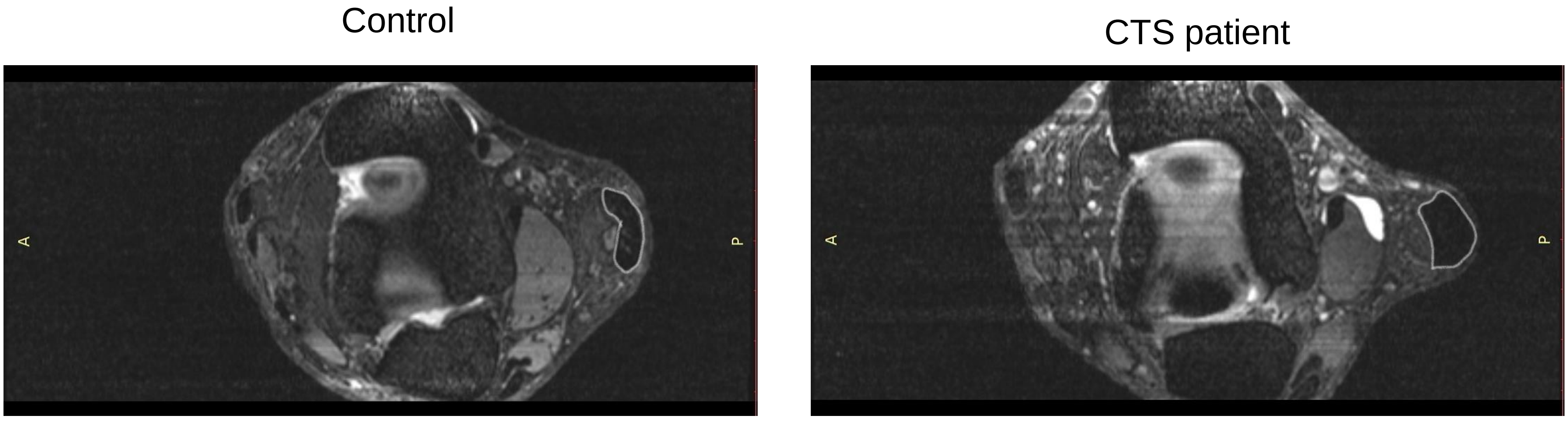

Supplementary Fig.4a

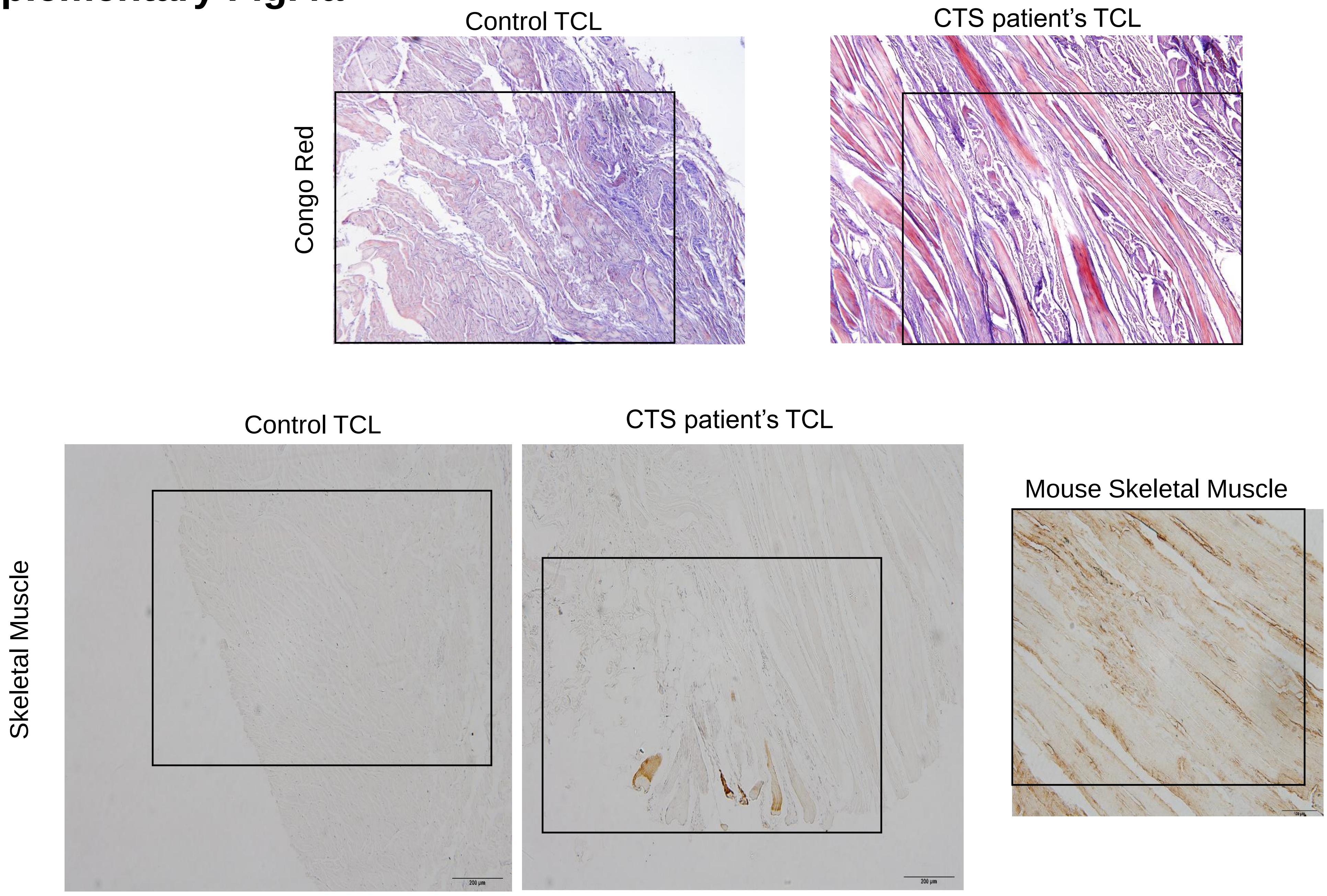

Supplementary Fig.4c

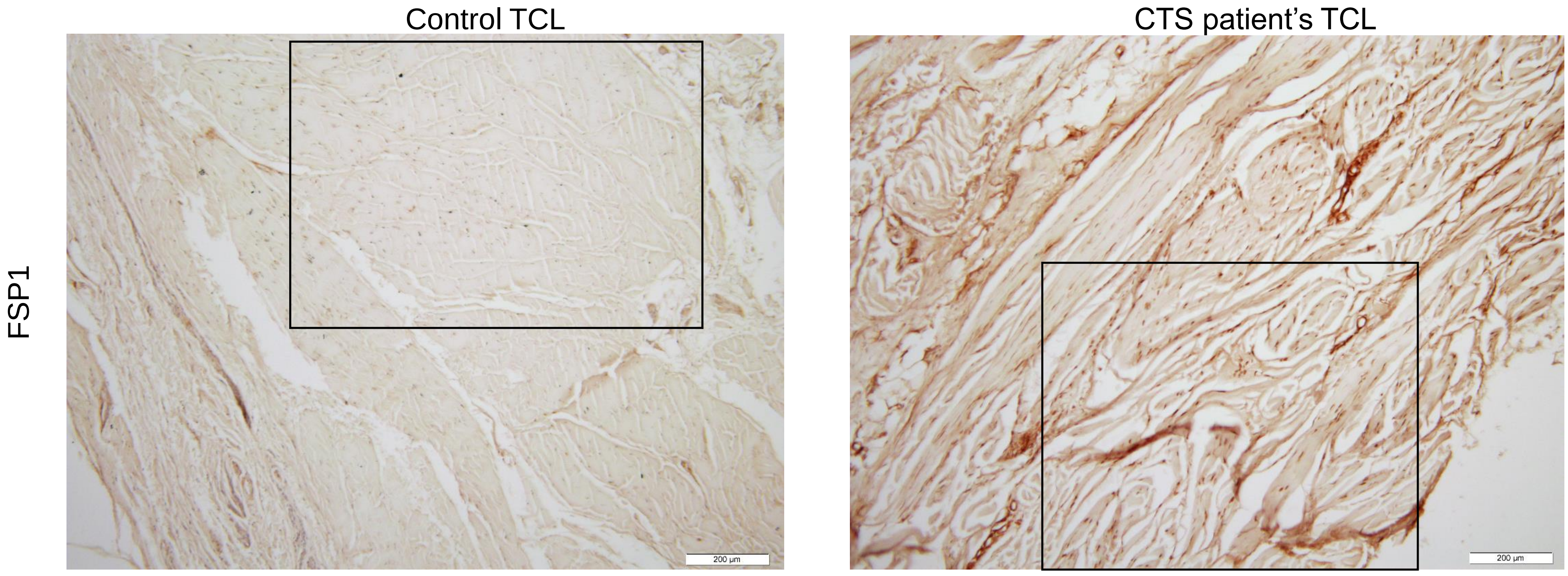

Supplementary Fig.5

Type I Collagen

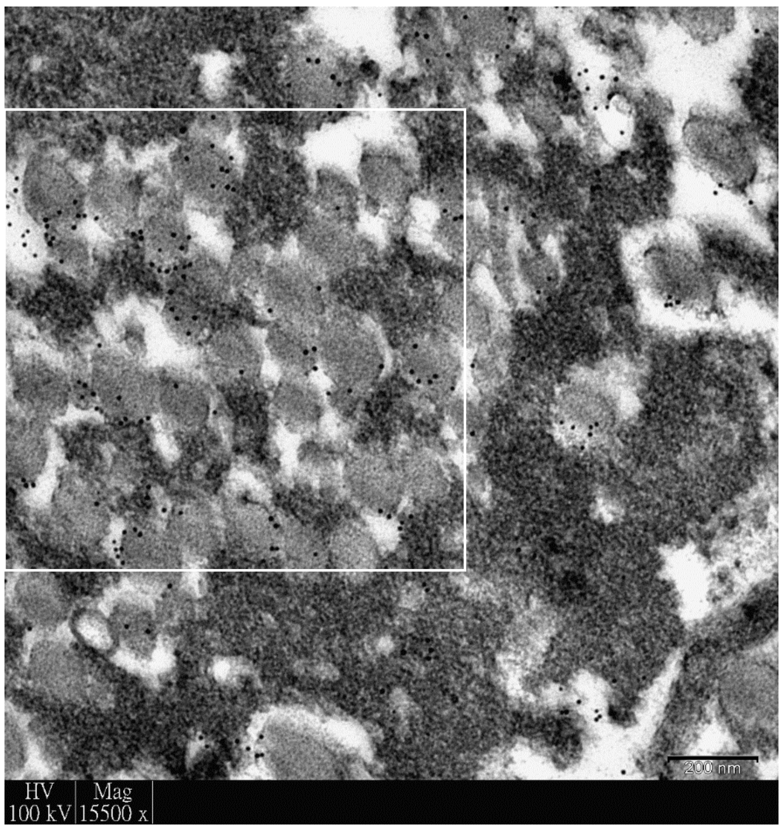

Type III Collagen

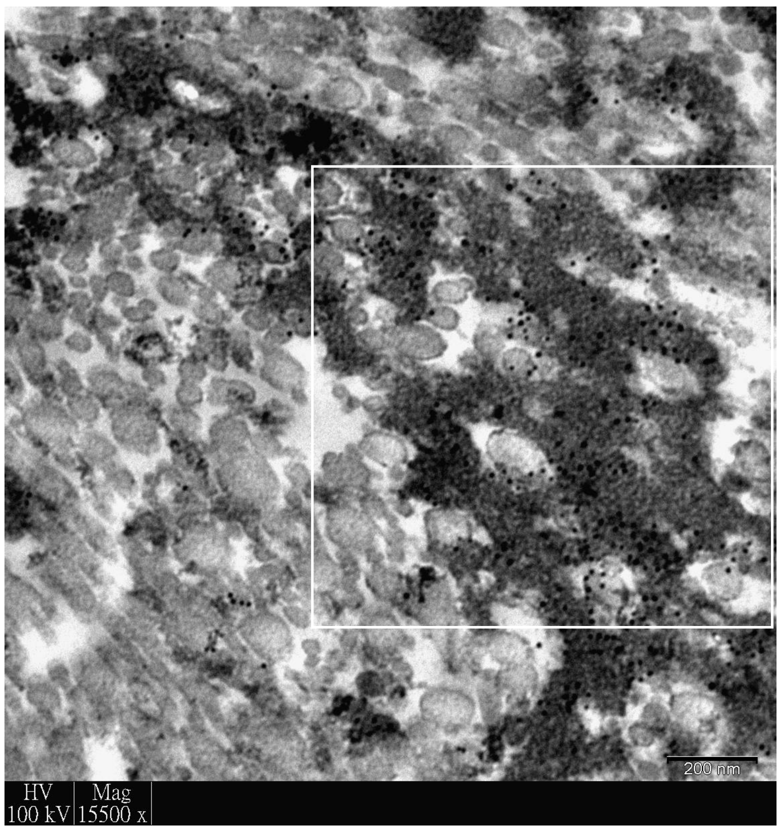

COMP

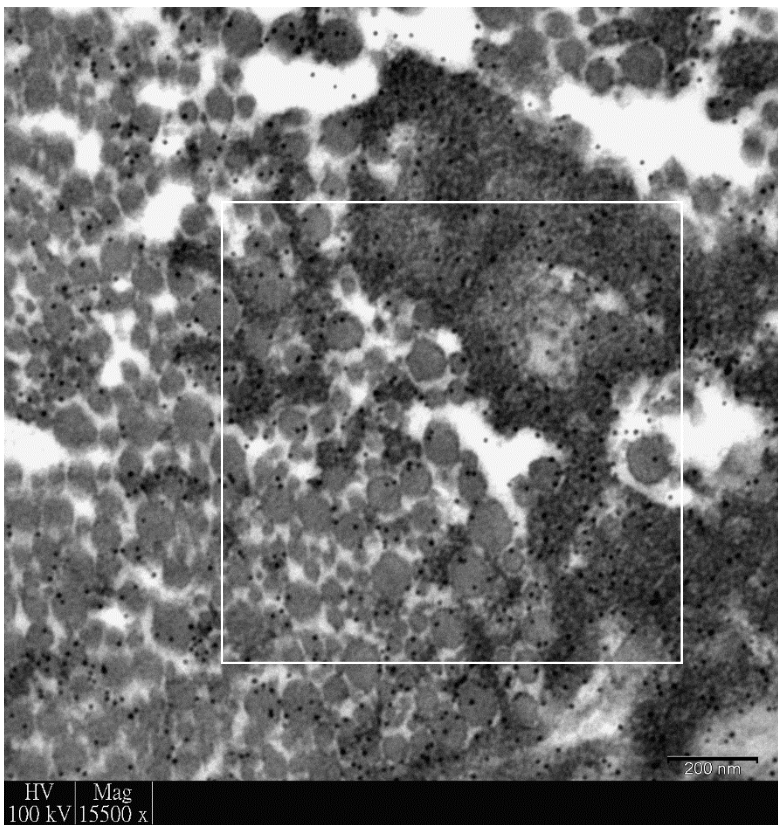

COMP

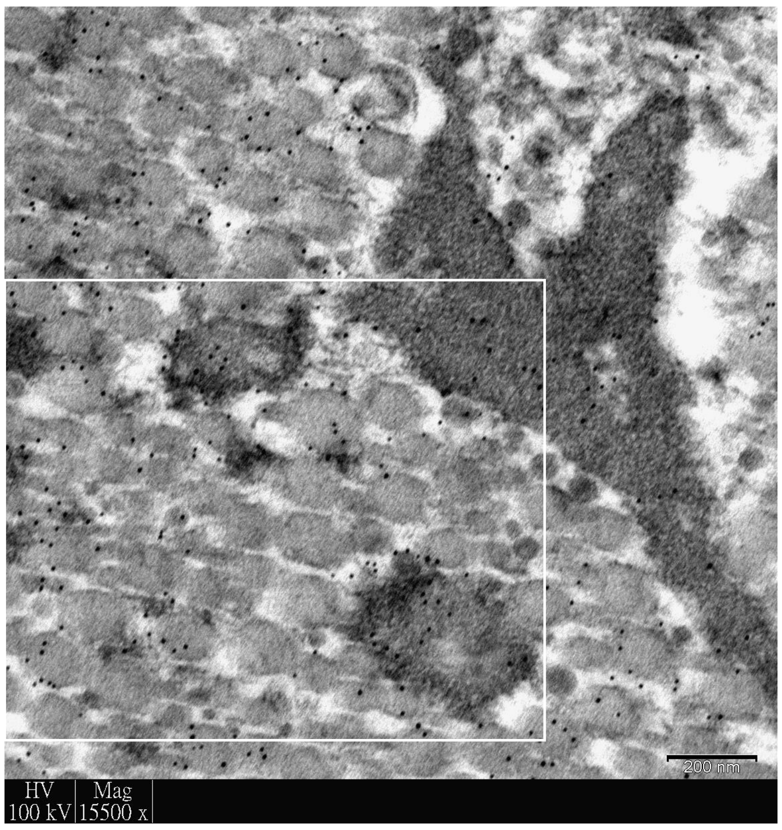

Supplementary Fig.6a

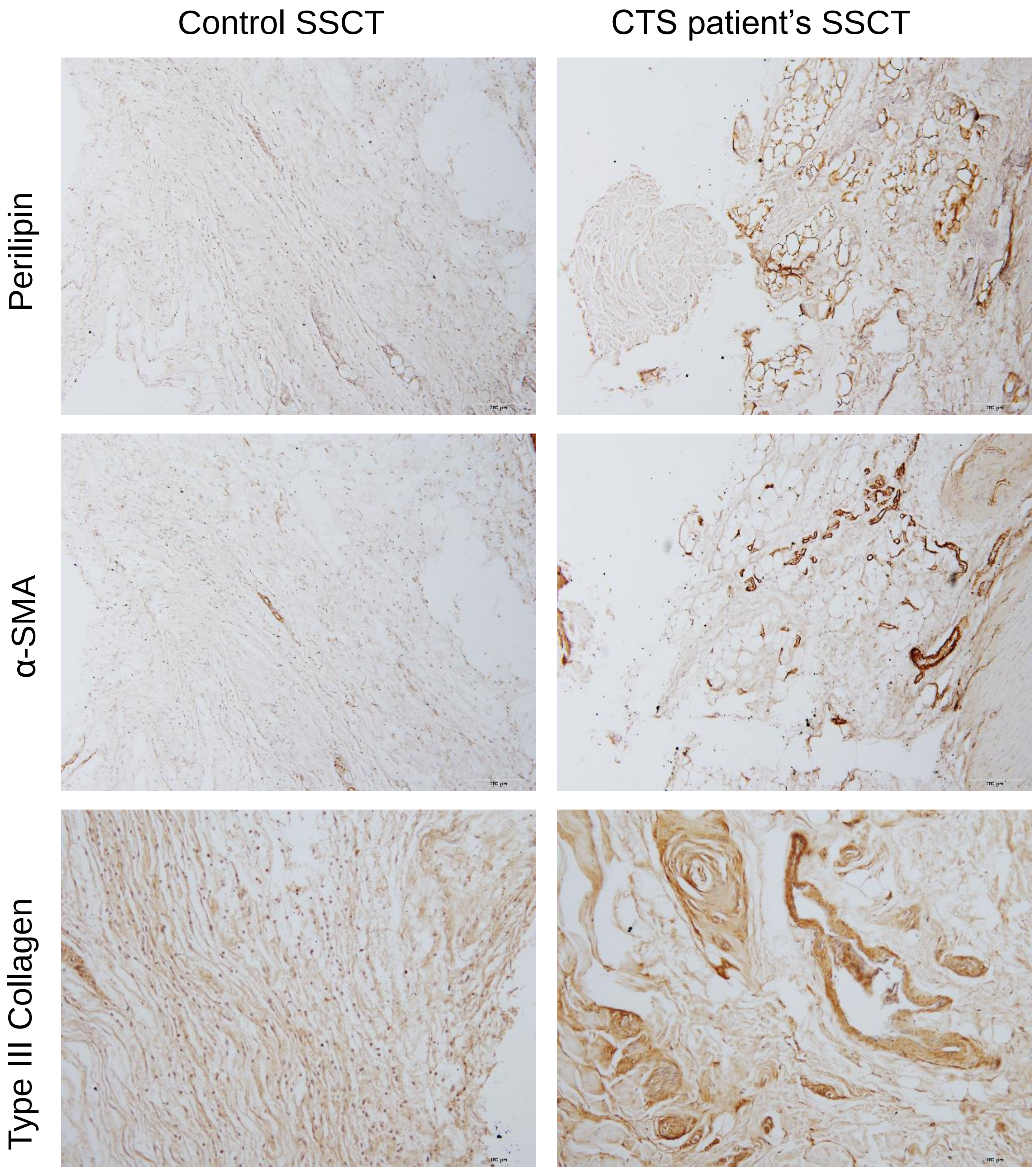

Supplementary Fig.6b

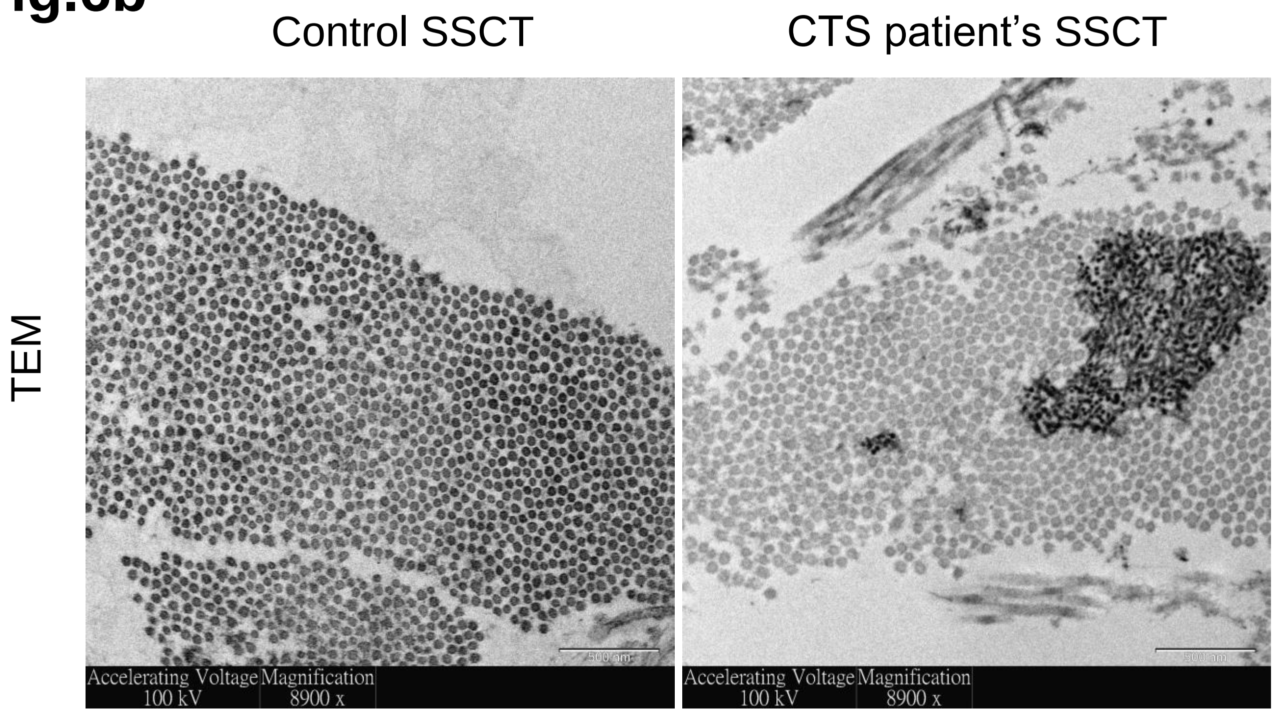

Supplementary Fig.6c

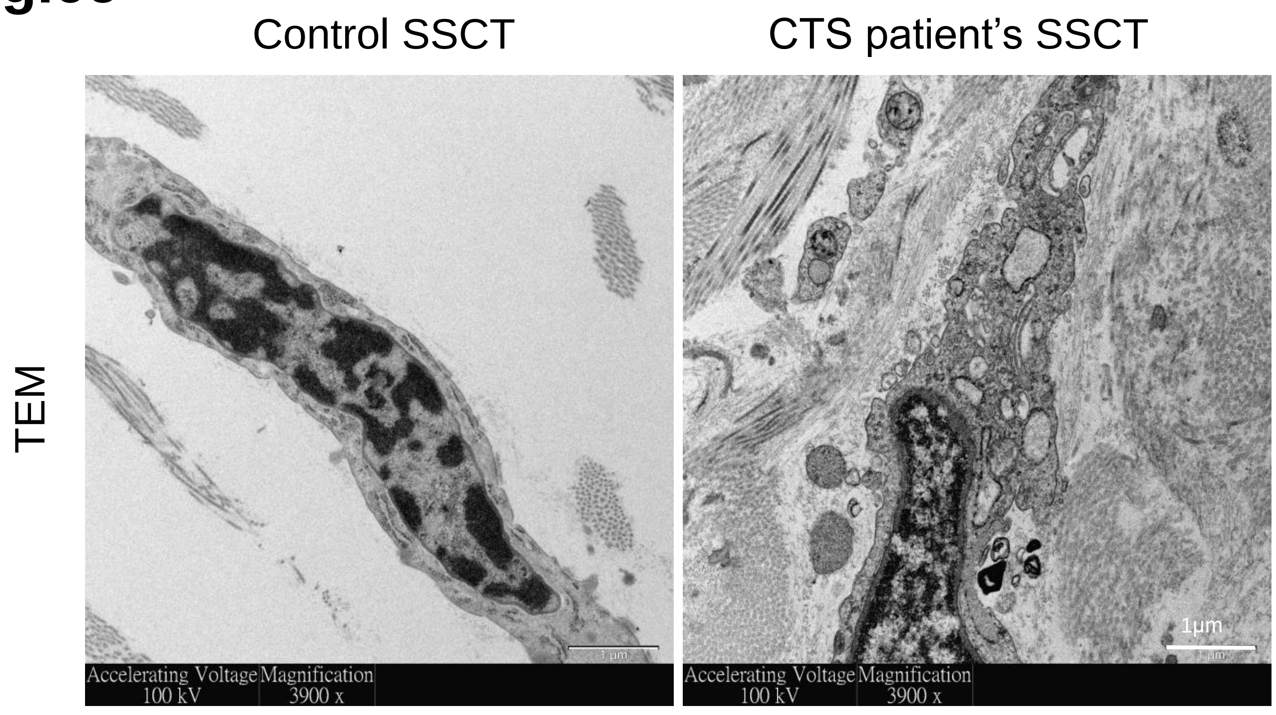

Supplementary Fig.7a

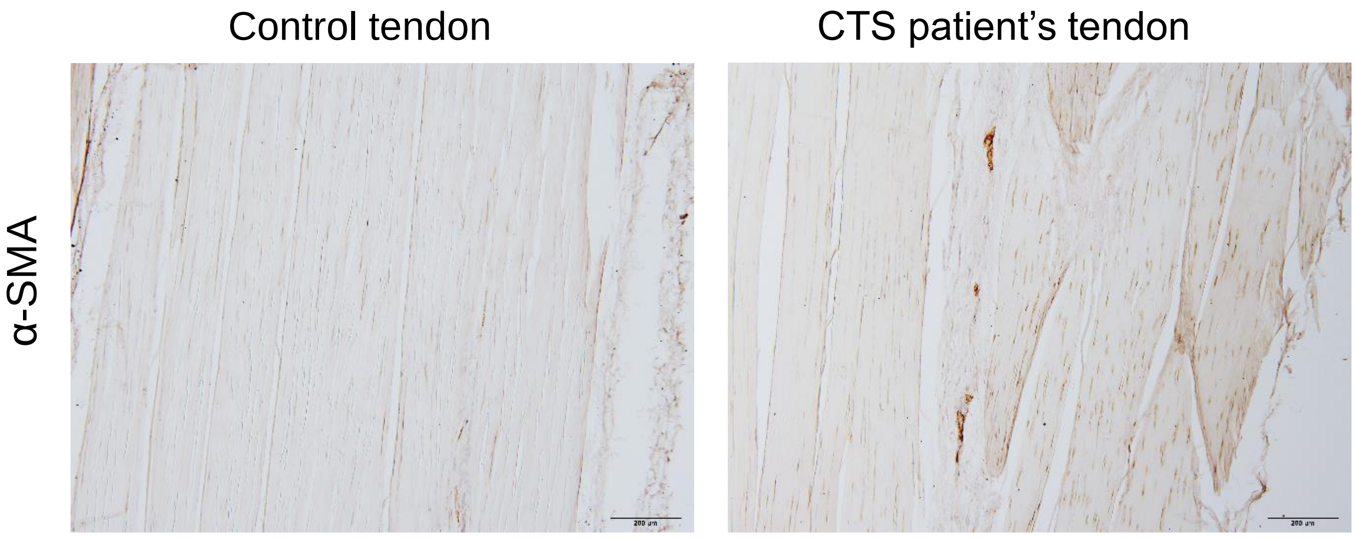

Supplementary Fig.7b

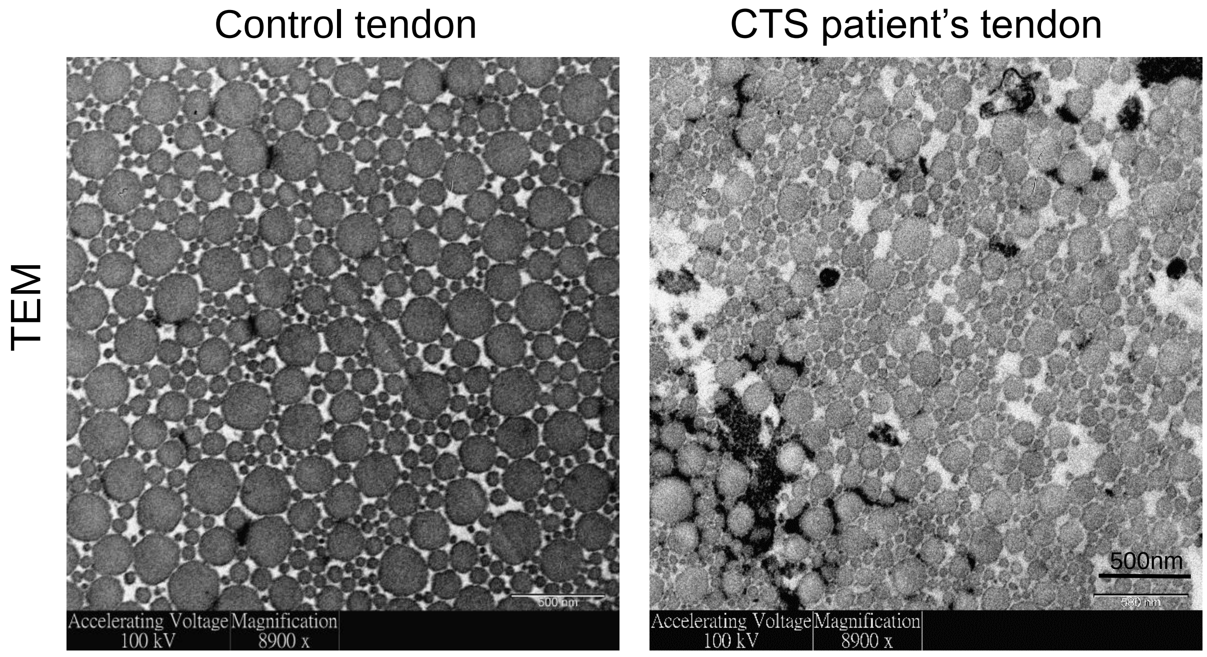

Supplementary Fig.7c

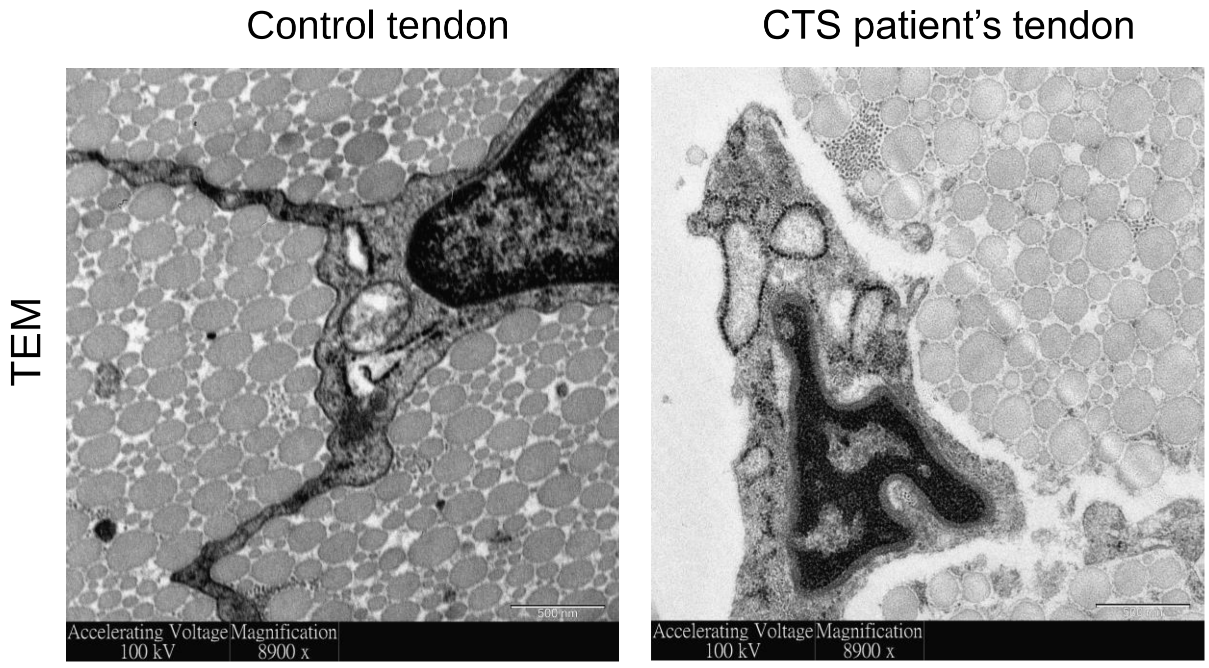

Supplementary Fig.7d

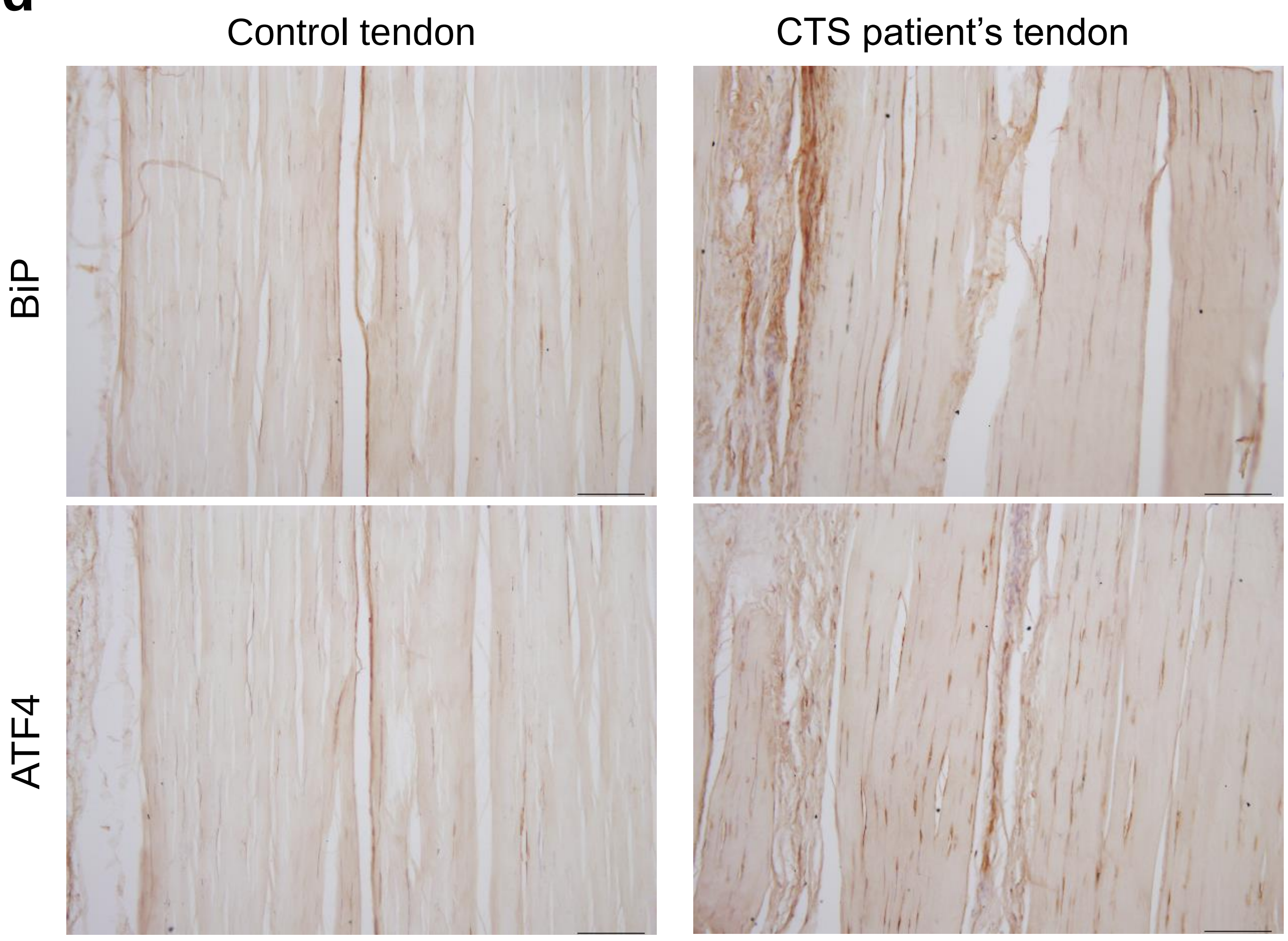

Supplementary Fig.9a

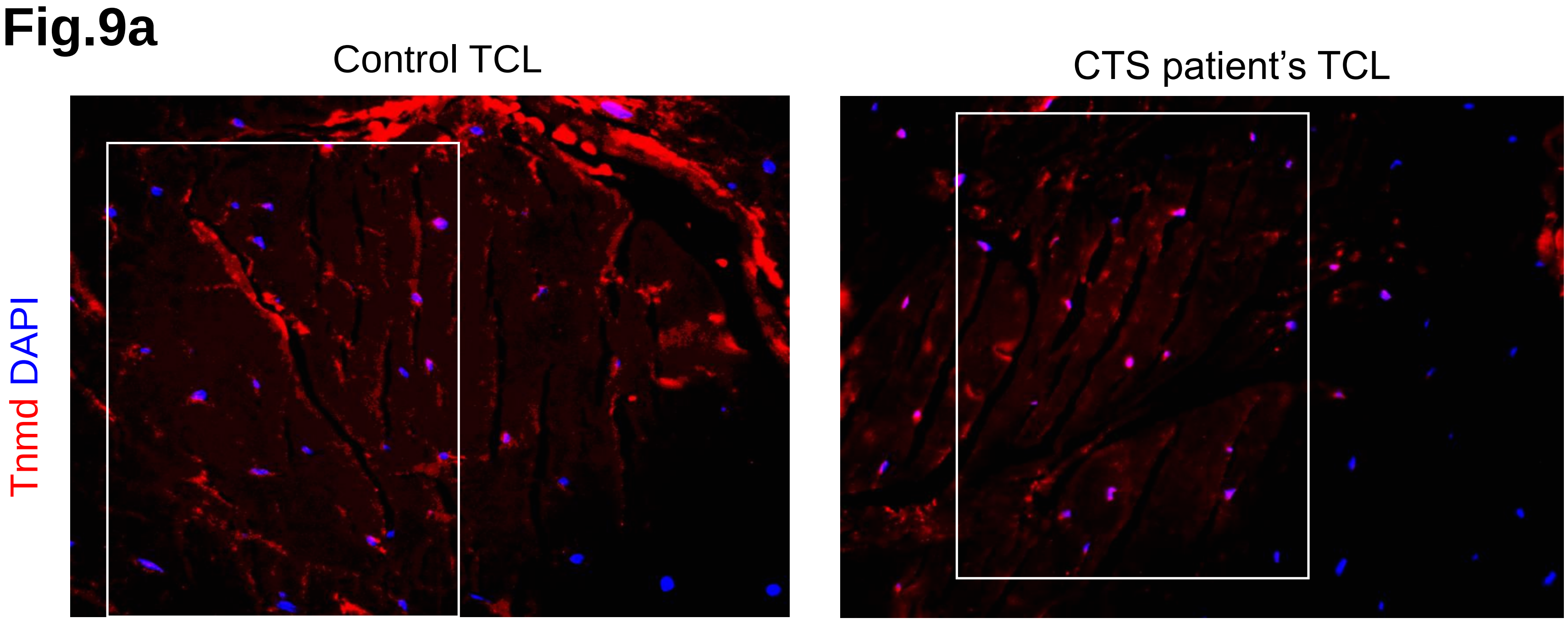

Supplementary Fig.9b

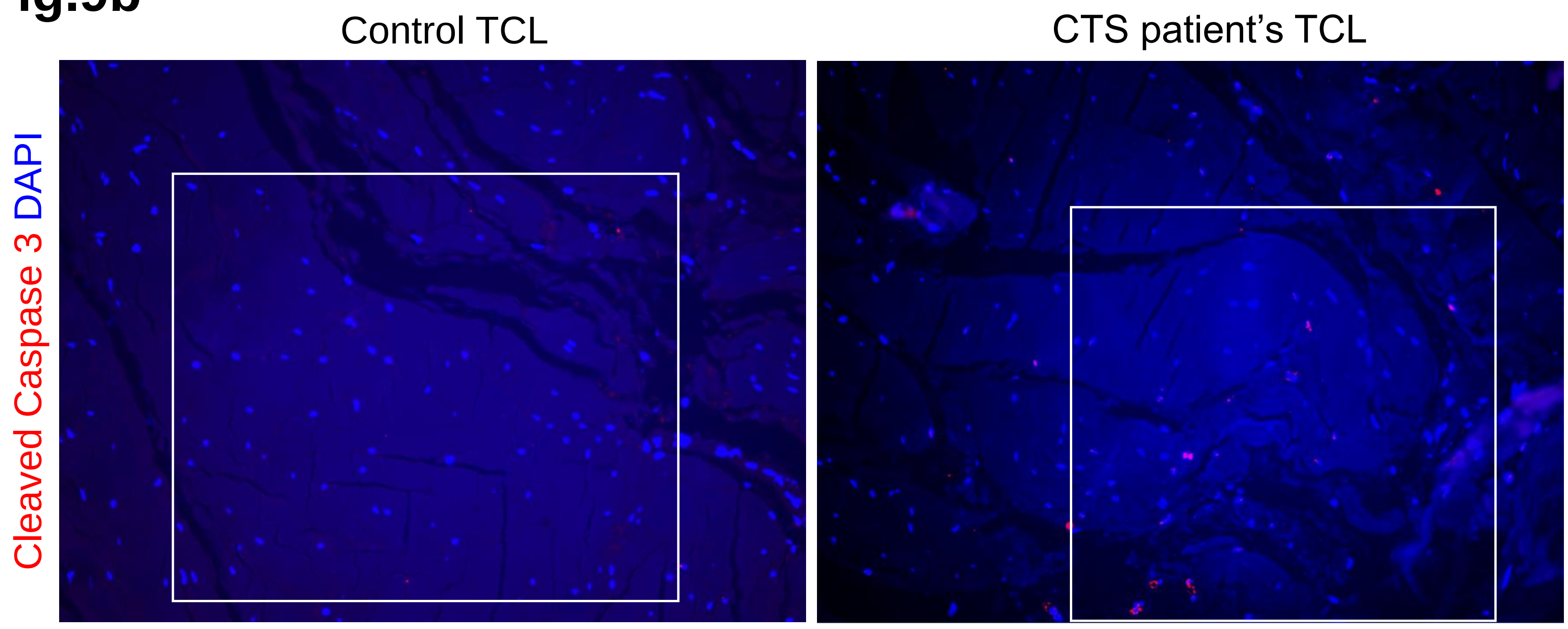

Supplementary Fig.9c

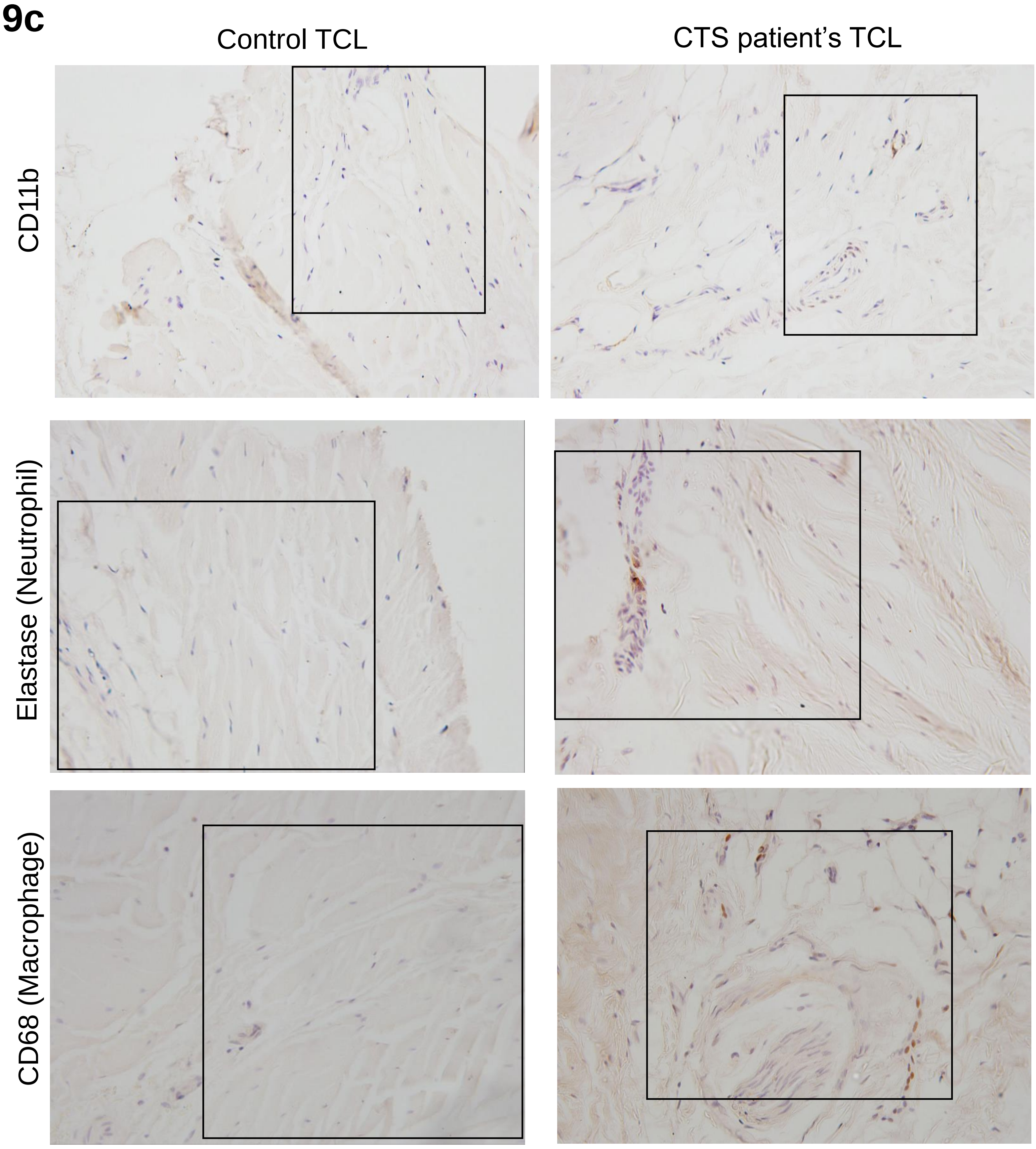

Supplementary Fig.11a

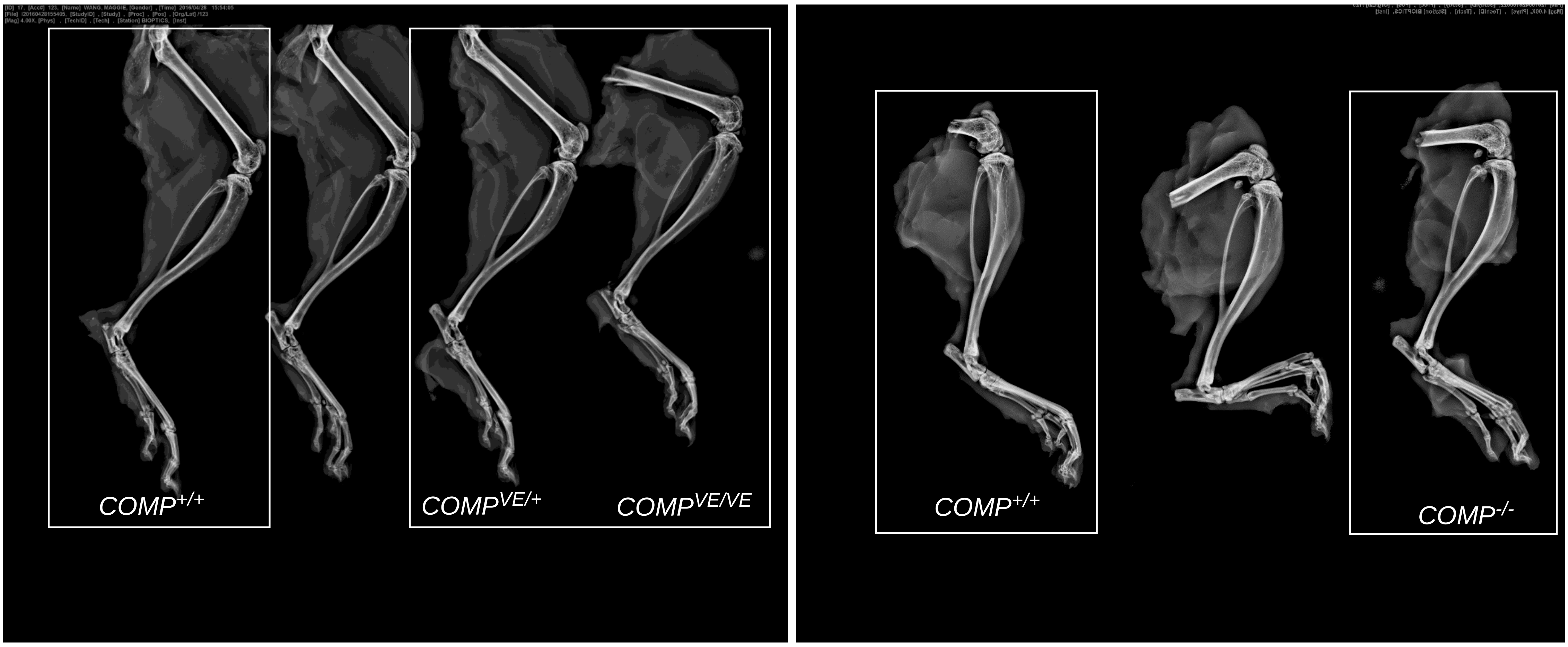

Supplementary Fig.11b

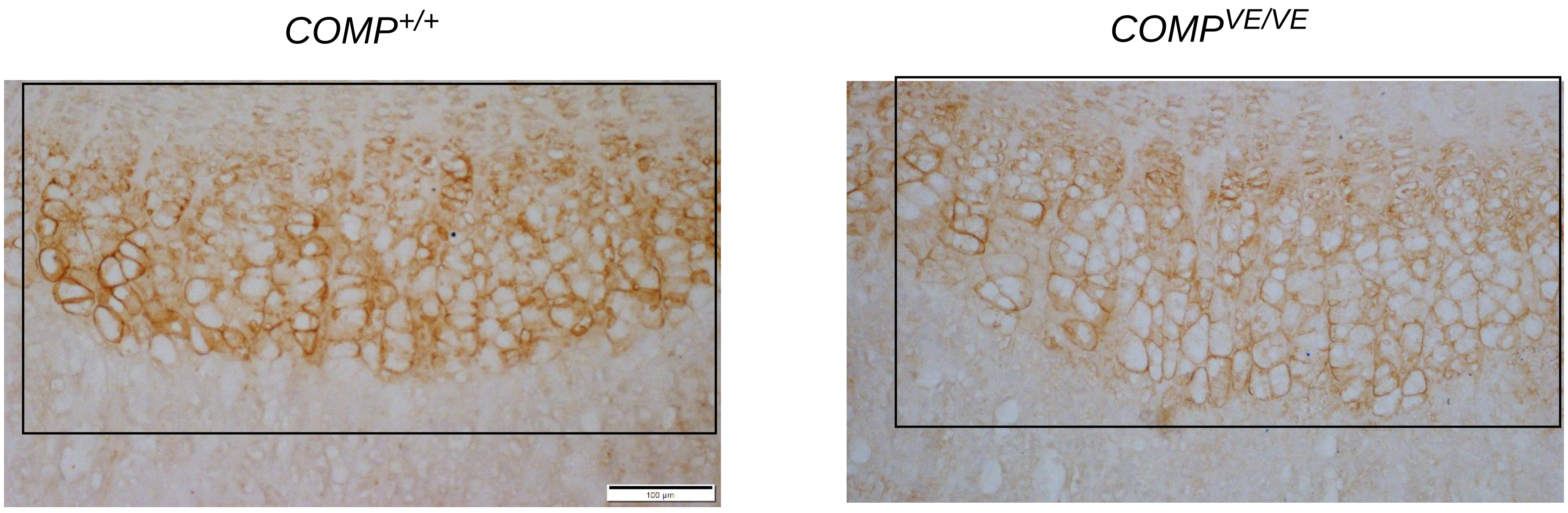

Supplementary Fig.12a

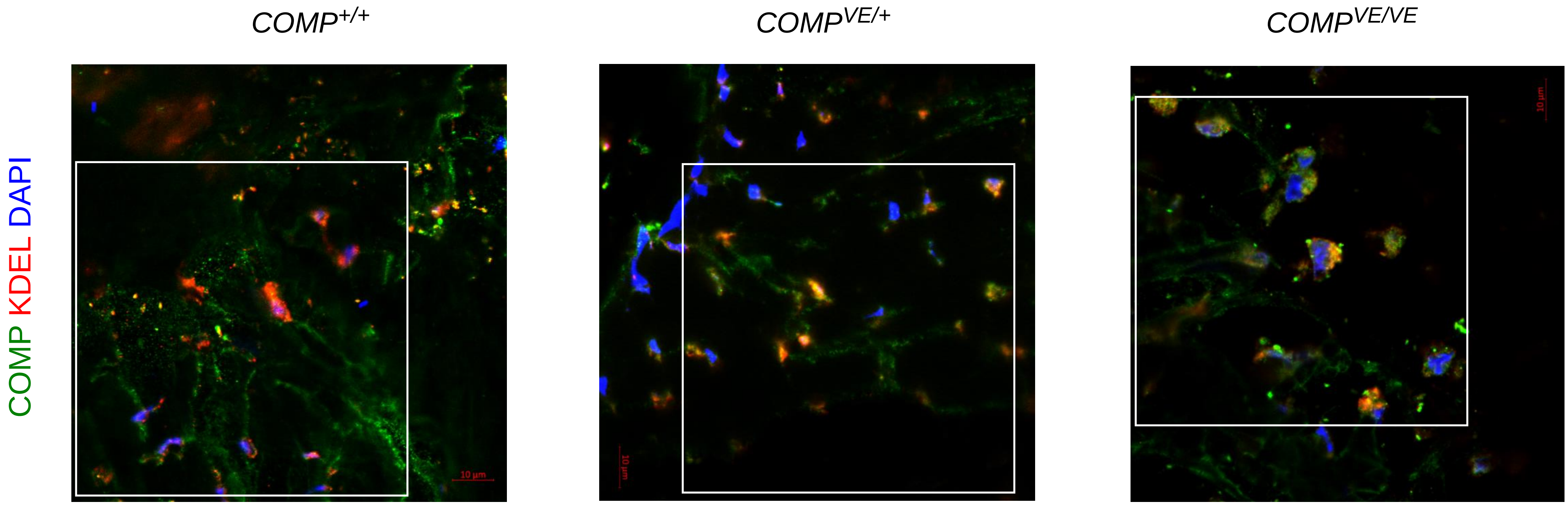

Supplementary Fig.12b (Upper Panel)

Bip

COMP$^{+/+}$

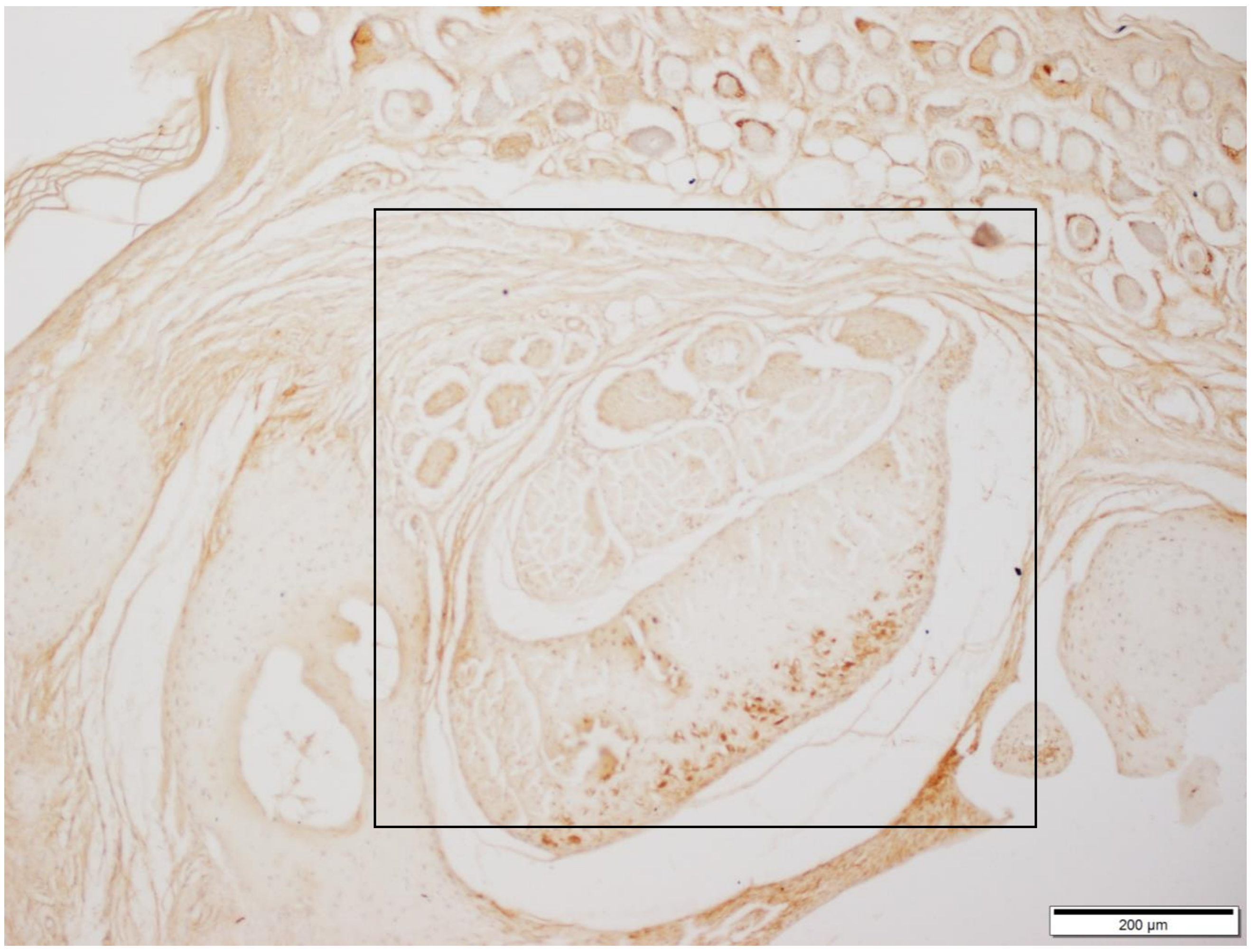

COMP$^{VE/+}$

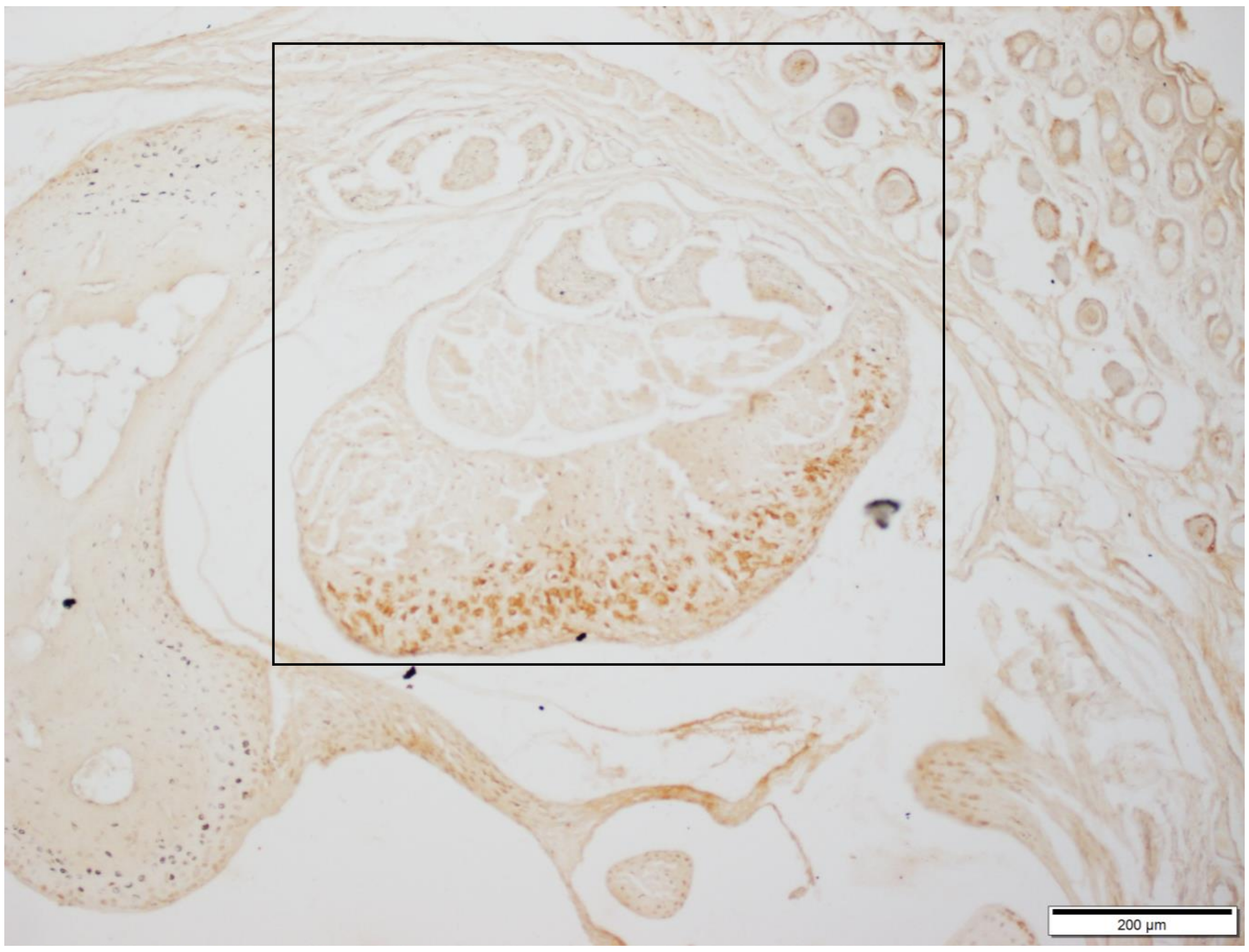

COMP$^{VE/VE}$

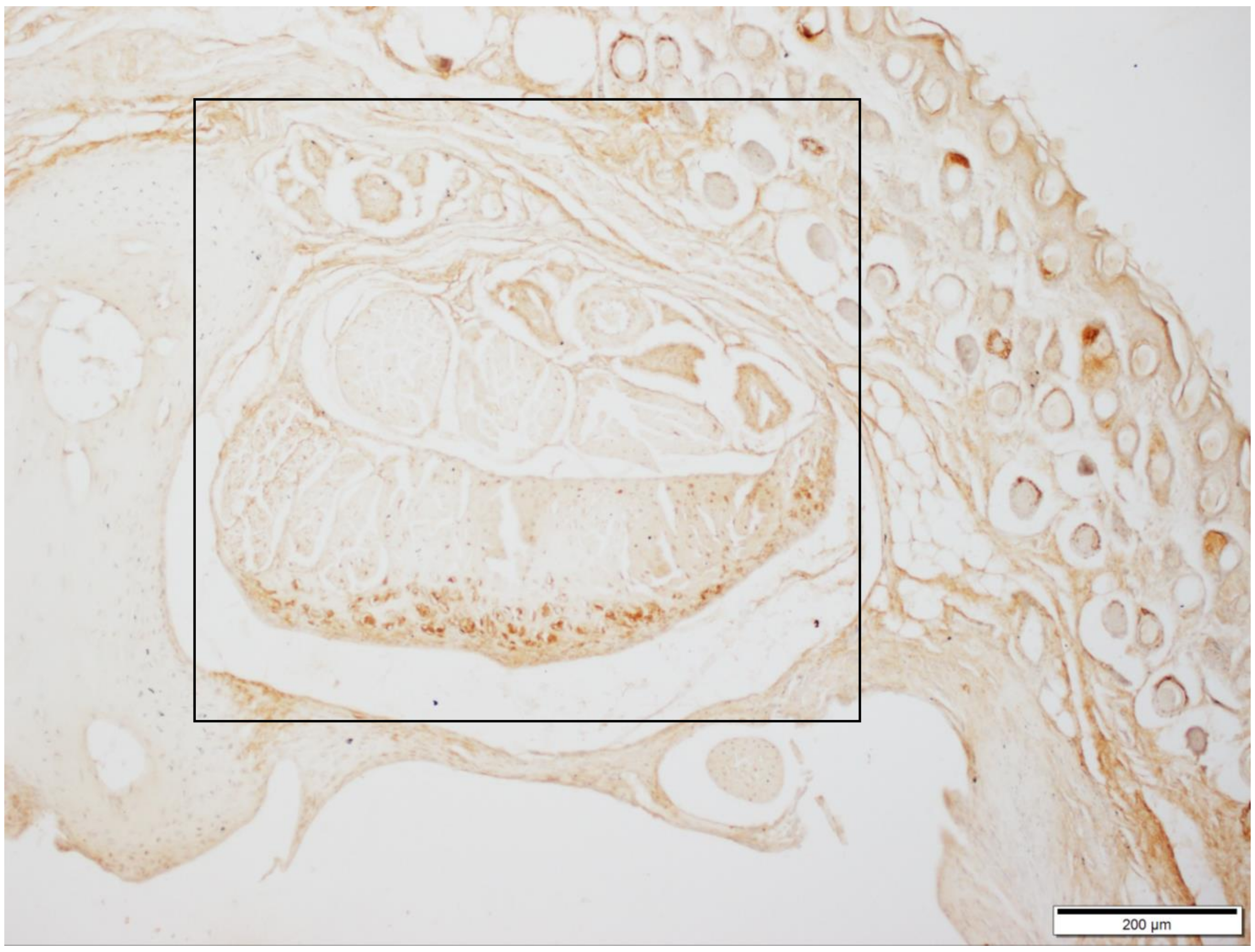

Supplementary Fig.12b (Lower Panel)

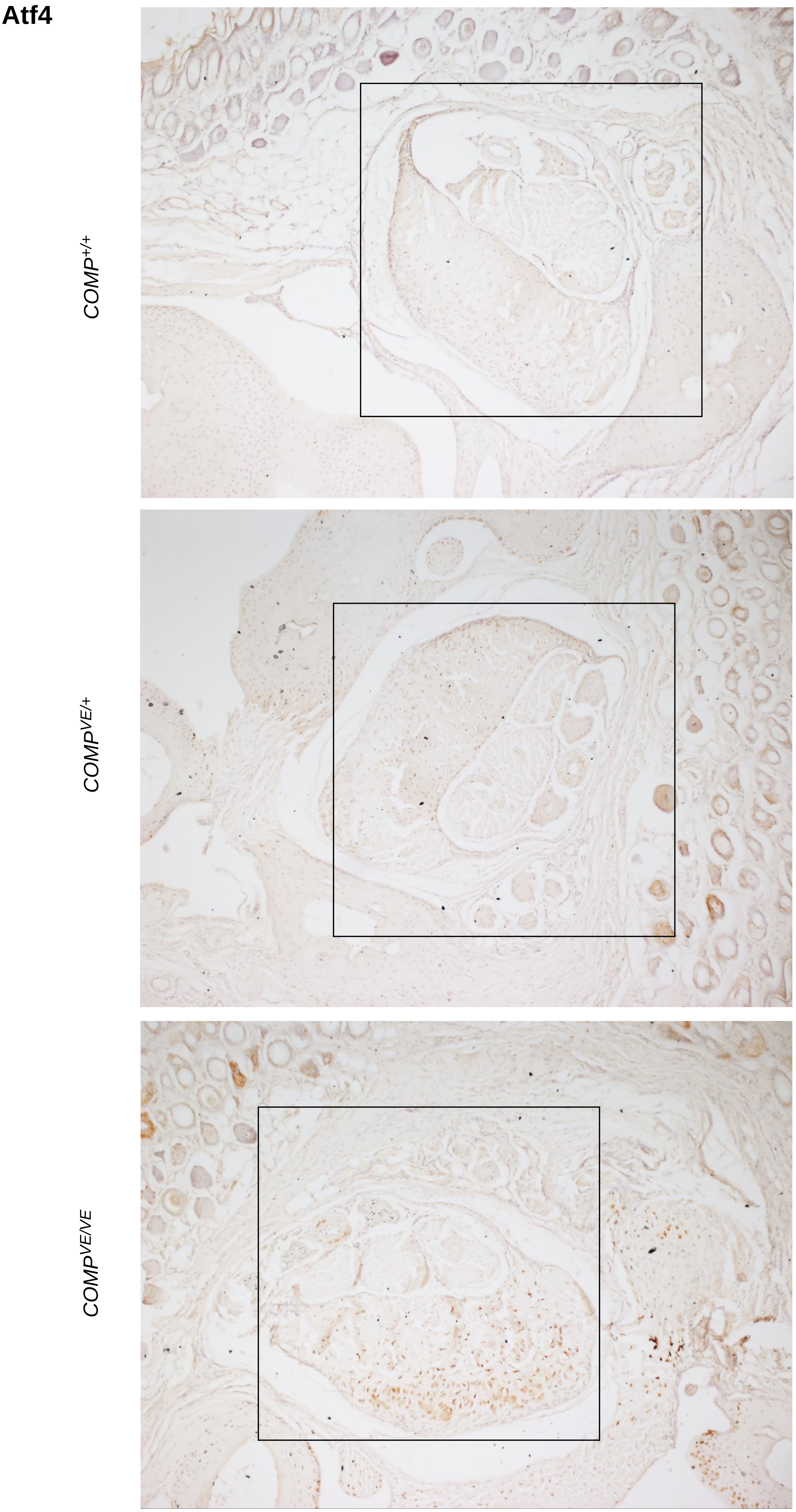

Supplementary Fig.13a

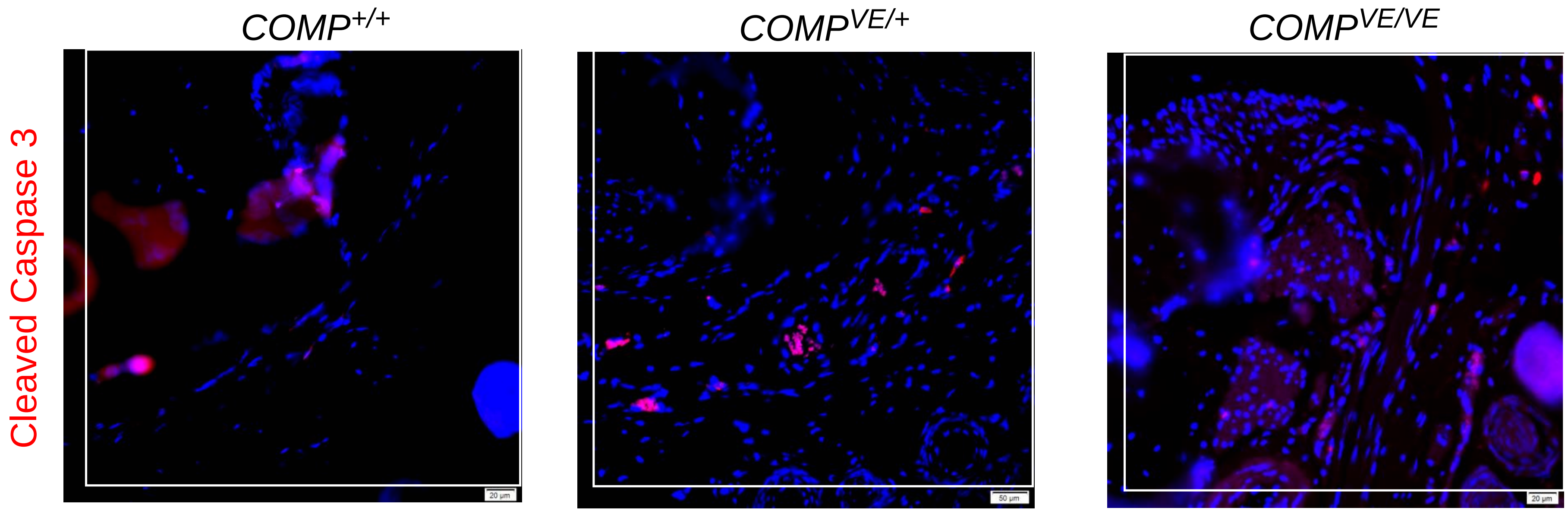

Supplementary Fig.13b

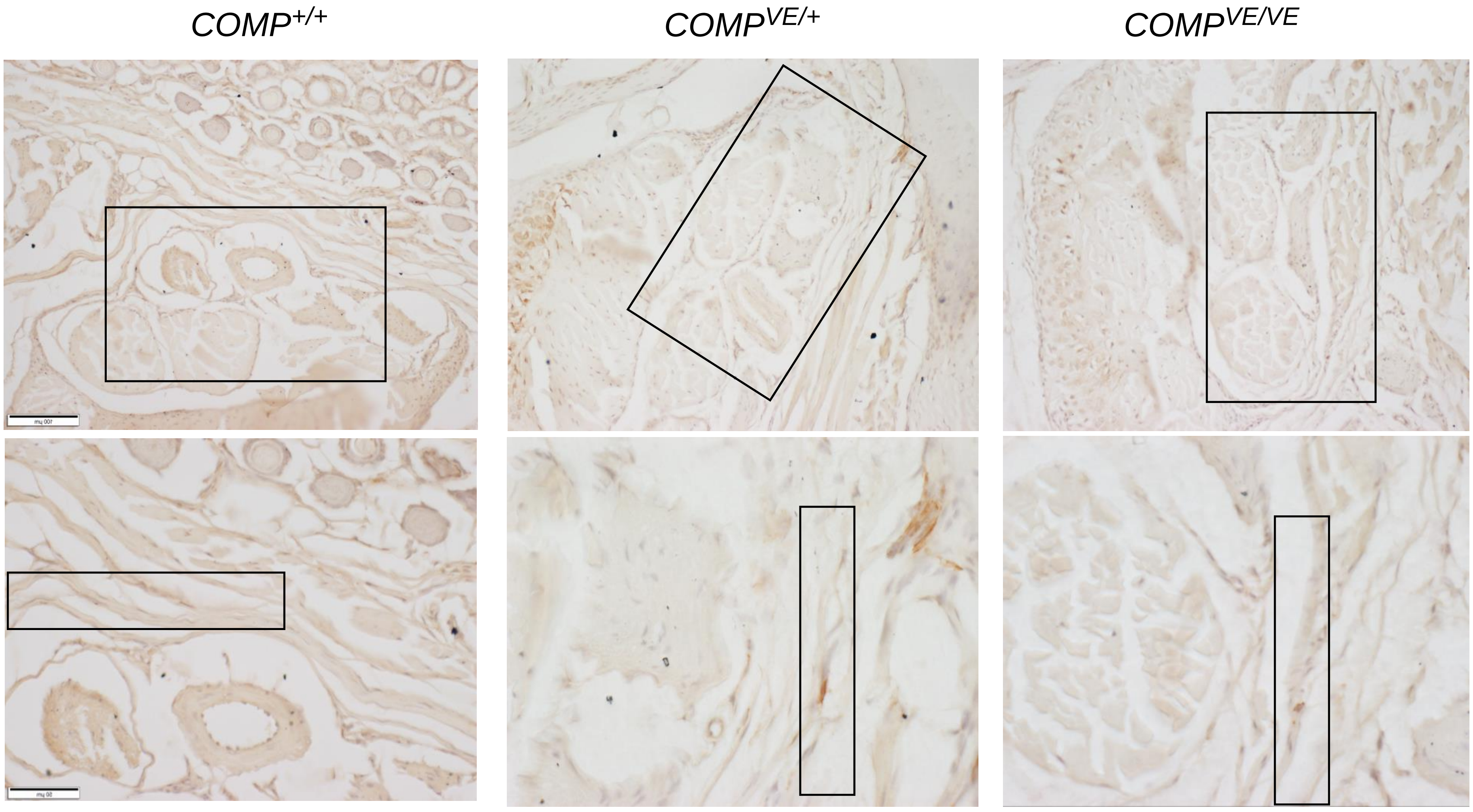

CD11b

Supplementary Fig.13c

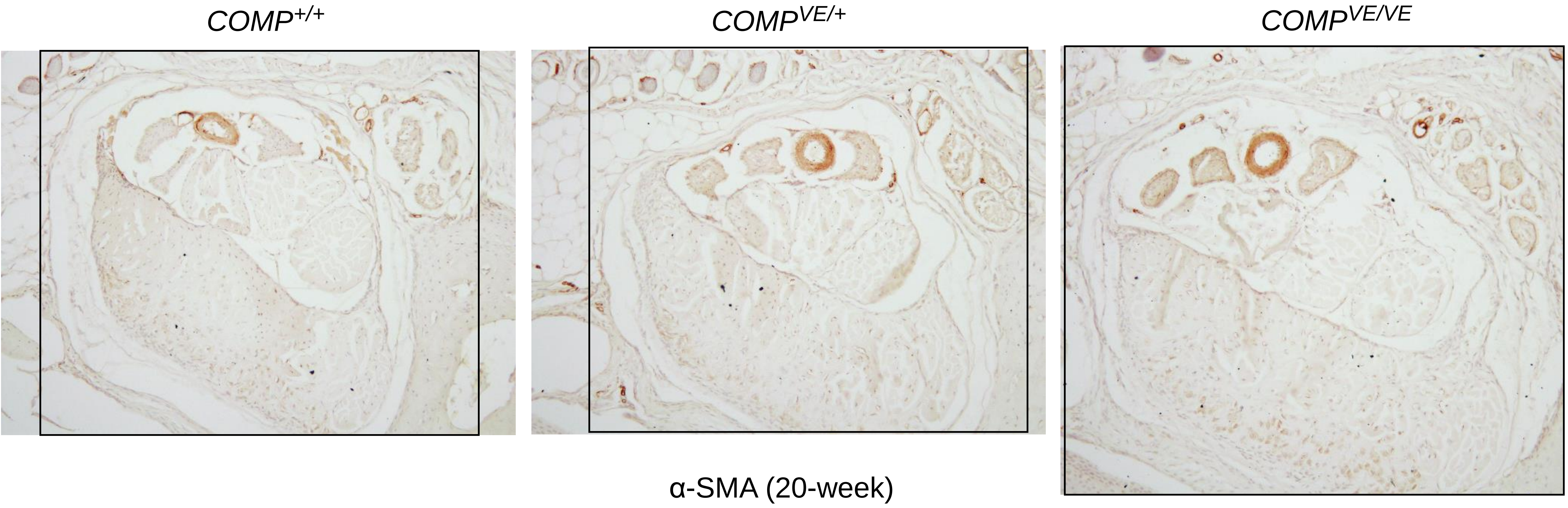

α-SMA (20-week)

Supplementary Fig.14a

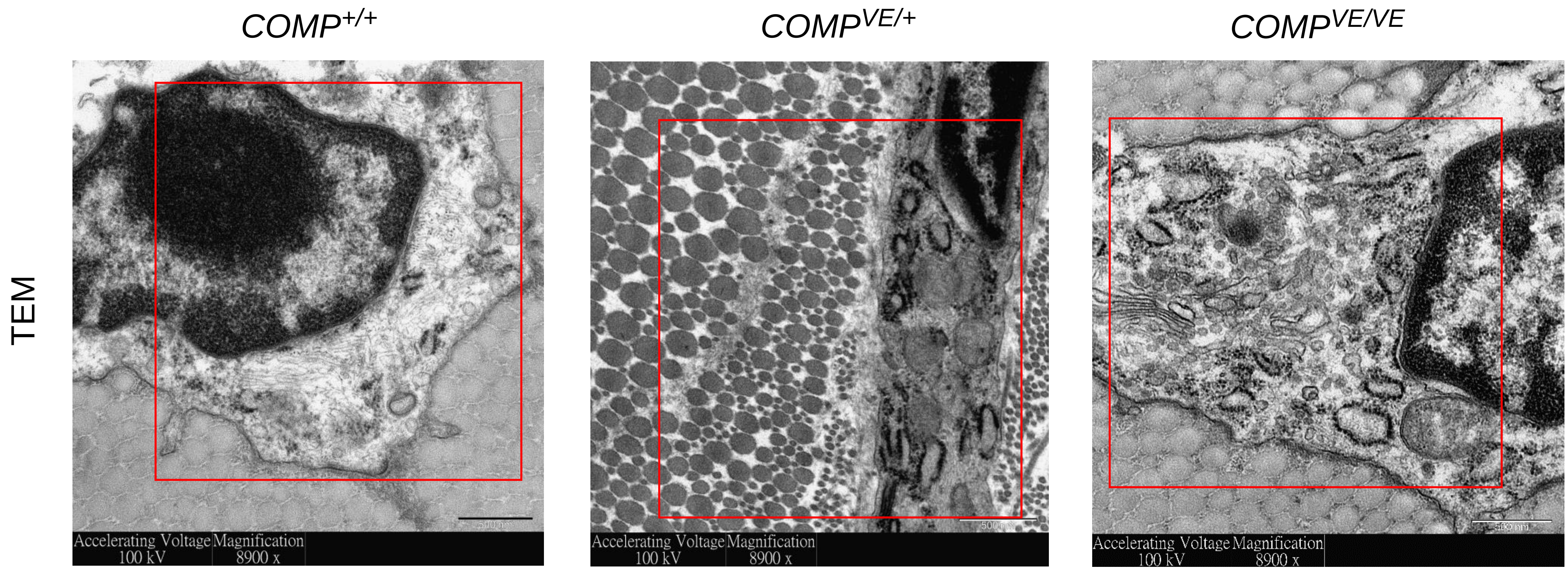

Supplementary Fig.14b

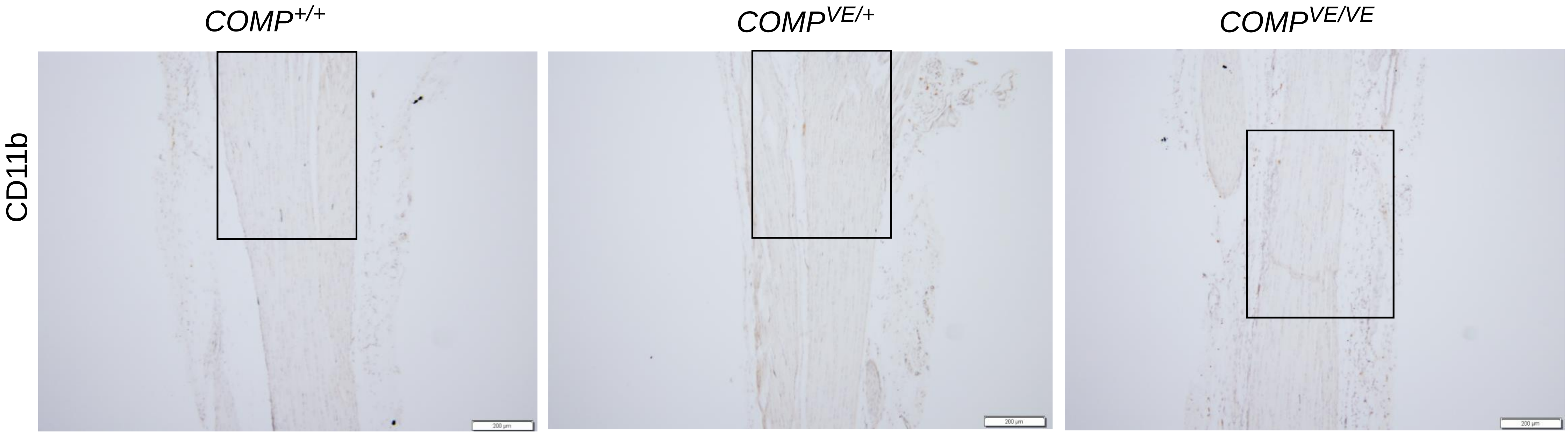

Supplementary Fig.14c

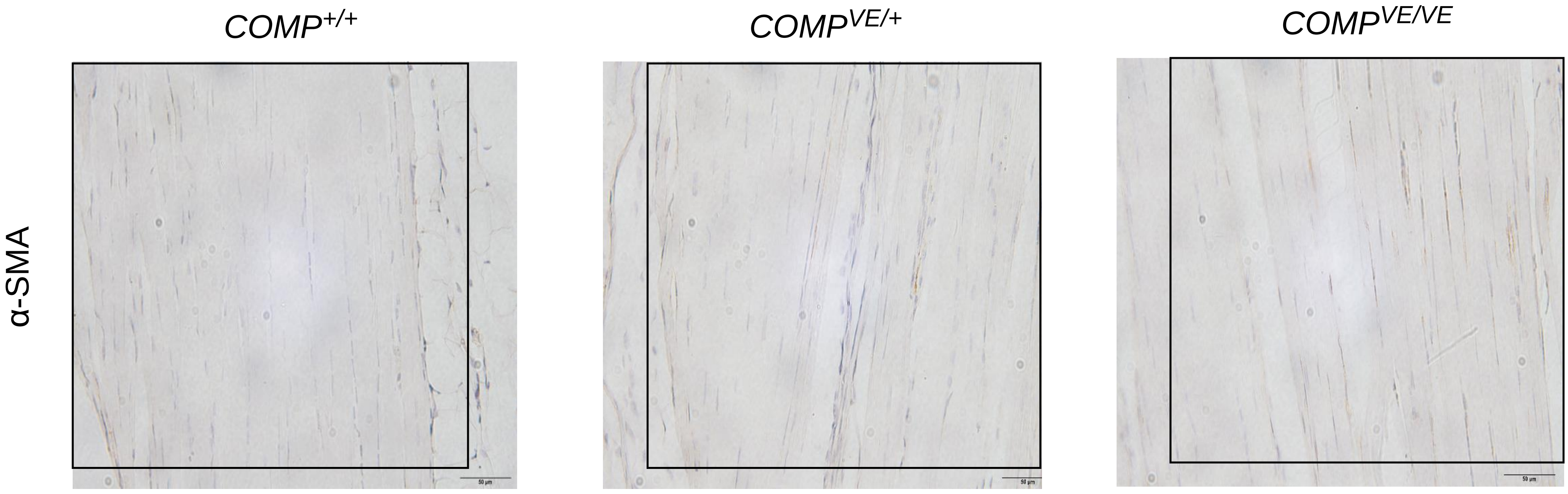

Supplementary Fig.15

COMP$^{+/+}$ *COMP*$^{VE/+}$ *COMP*$^{VE/VE}$

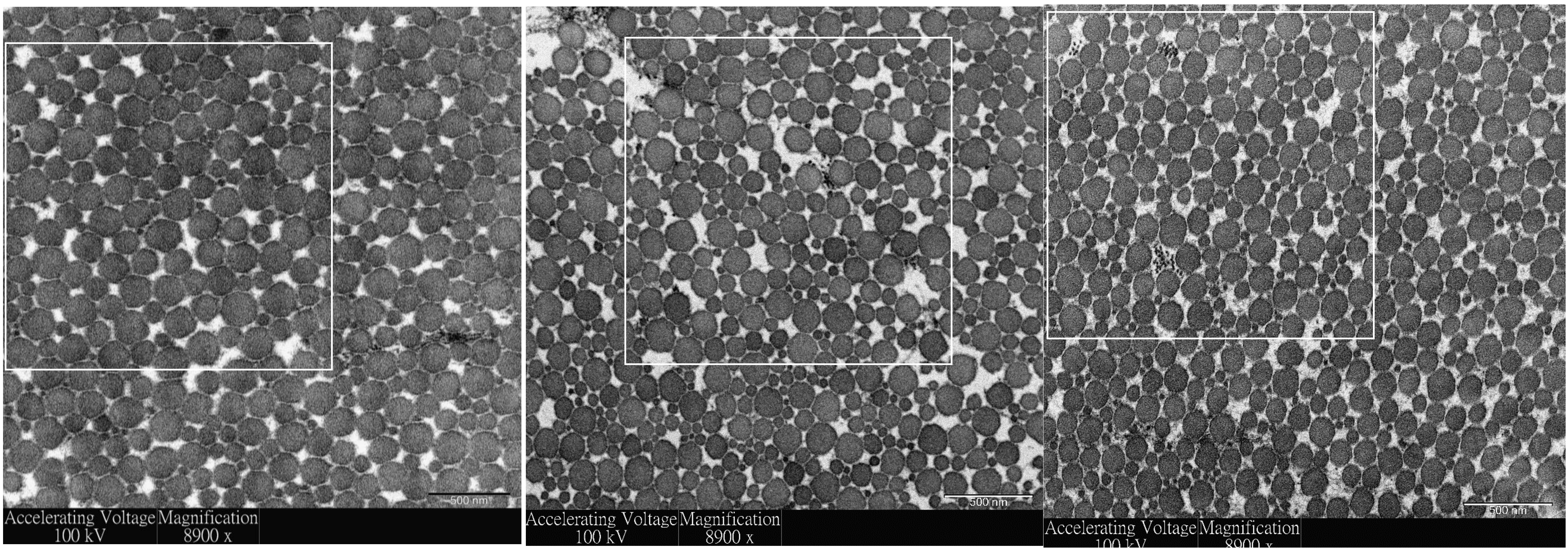

20-week

COMP$^{+/+}$ *COMP*$^{VE/+}$ *COMP*$^{VE/VE}$

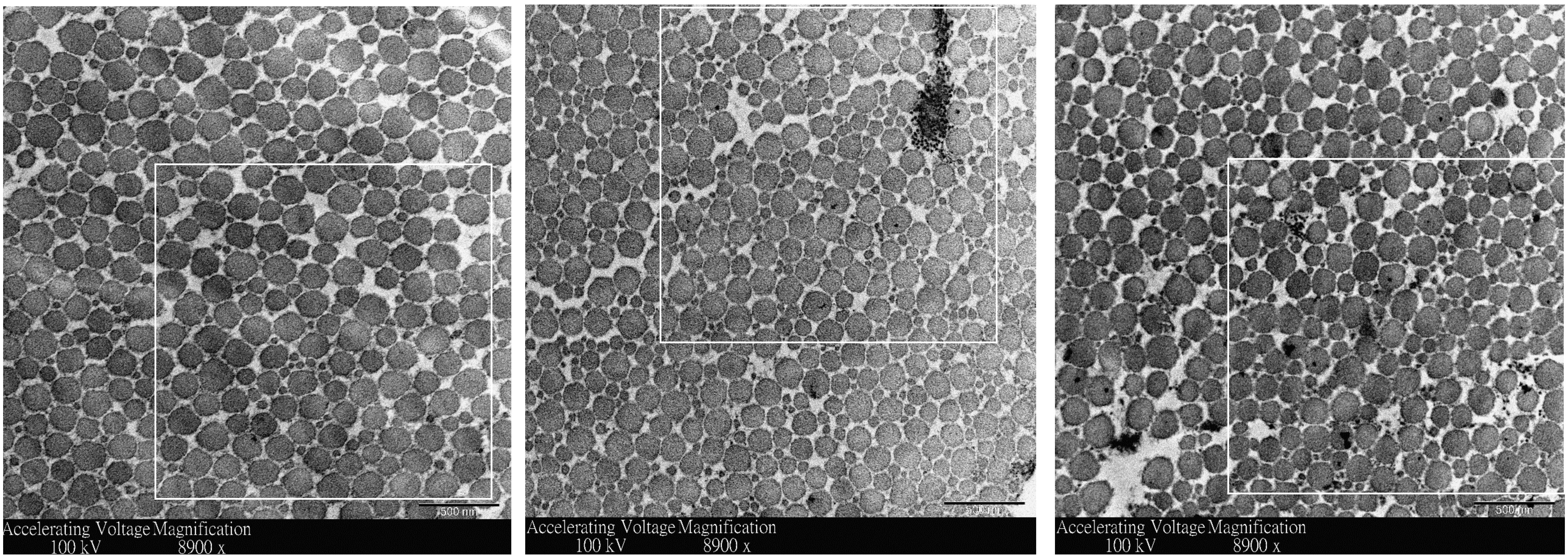

20-month

Supplementary Fig.16b

COMP$^{+/+}$ *COMP*$^{VE/+}$ *COMP*$^{VE/VE}$

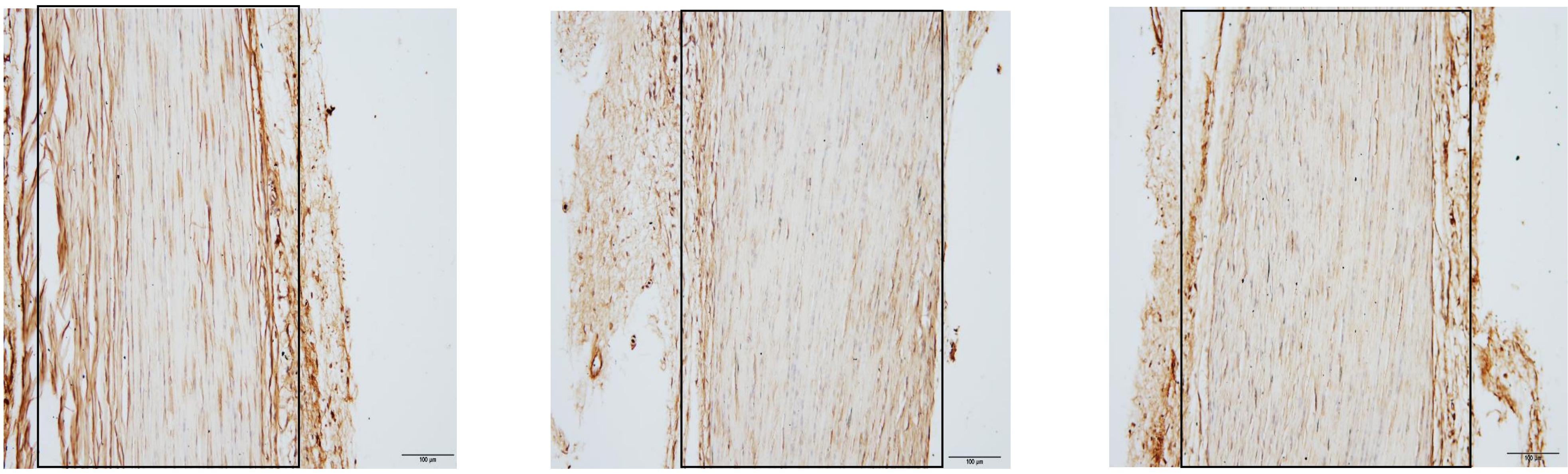

Fmod

Supplementary Fig.17b

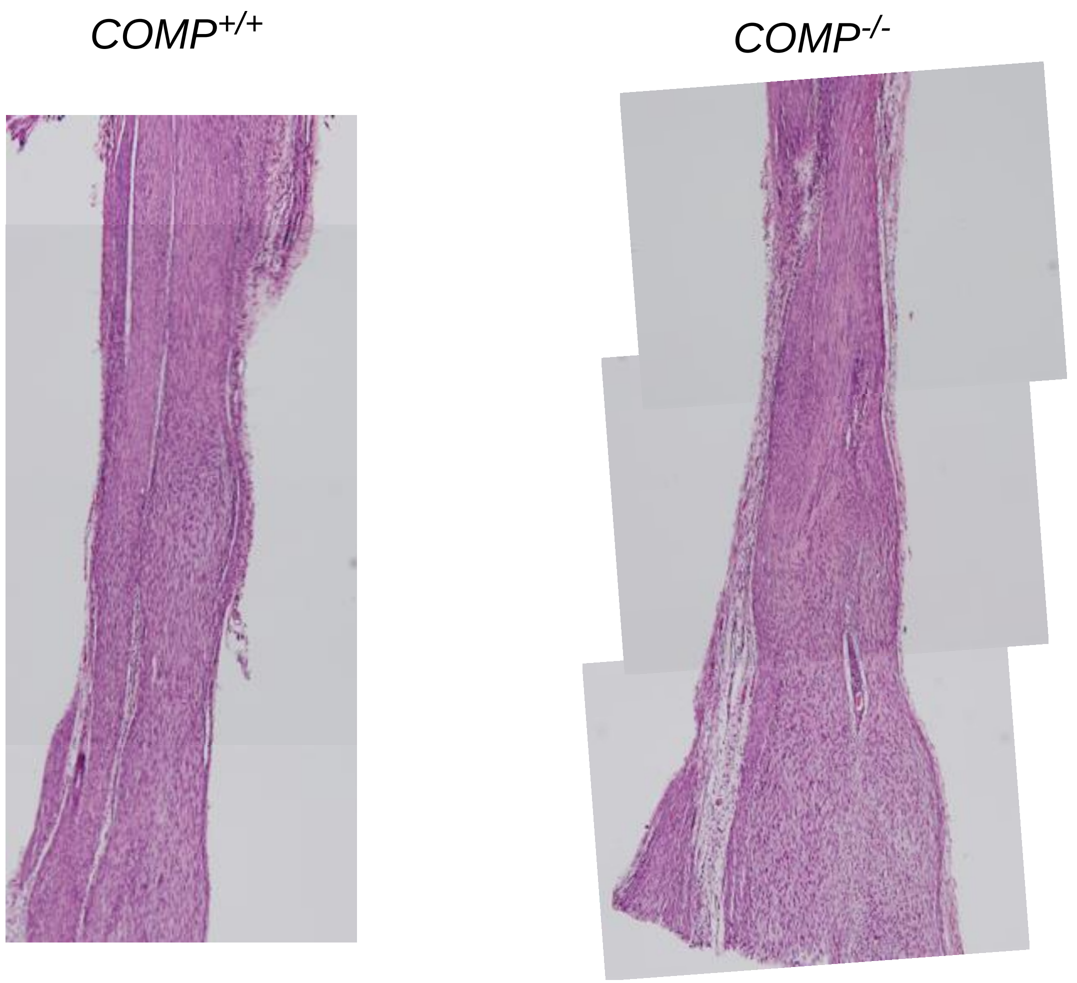

Supplementary Fig.17c

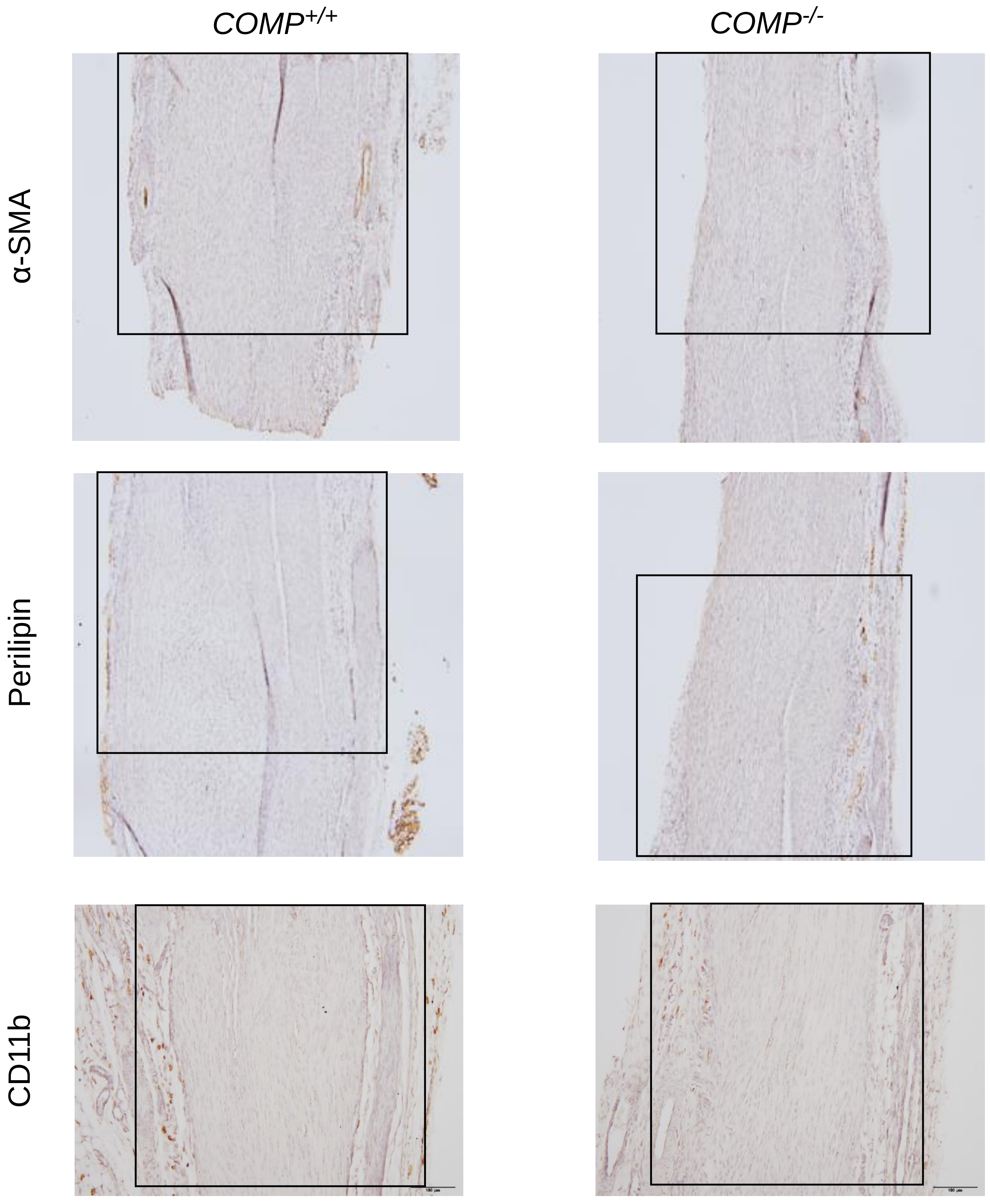

Supplement: Supplementary file 3 — Source Data [file 41467_2020_17378_MOESM3_ESM.zip › Source Data.pdf]
